# GONet: a molecular interaction-guided graph learning framework for multi-omics cancer classification

**DOI:** 10.1093/gigascience/giag097

**Published:** 2026-10-22

**Authors:** Yiyang Chen, Yunning Lu, Da Qi, Yong Bai, Guixue Hou, Siqi Liu

**Affiliations:** College of Life Sciences, University of Chinese Academy of Sciences, No. 1 Yanqihu East Rd, Huairou District,Beijing,100049, China; Department of Proteomics, BGI-Genomics, No. 9 Yunhua Road, Yantian District, Shenzhen, Guangdong, 518083, China; Department of Proteomics, BGI-Genomics, No. 9 Yunhua Road, Yantian District, Shenzhen, Guangdong, 518083, China; School of Biology and Biological Engineering, South China University of Technology, Waihuan Dong Rd, Panyu District, Guangzhou, Guangdong, 510006, China; Department of Proteomics, BGI-Genomics, No. 9 Yunhua Road, Yantian District, Shenzhen, Guangdong, 518083, China; BGI Research, 146 Beishan Road, Yantian District, Shenzhen, Guangdong, 518083, China; Department of Proteomics, BGI-Genomics, No. 9 Yunhua Road, Yantian District, Shenzhen, Guangdong, 518083, China; College of Life Sciences, University of Chinese Academy of Sciences, No. 1 Yanqihu East Rd, Huairou District,Beijing,100049, China; Department of Proteomics, BGI-Genomics, No. 9 Yunhua Road, Yantian District, Shenzhen, Guangdong, 518083, China

**Keywords:** multi-omics data integration, graph neural network, cancer classification, molecular interaction network

## Abstract

**Background:**

Integrating multi-omics profiles with molecular interaction networks enables biologically informed representation learning by combining molecular measurements with prior biological knowledge. Although graph neural networks (GNNs) are well suited for modeling molecular interaction networks, existing multi-omics methods often rely on shallow representation learning or incomplete fusion strategies, which fail to fully capture complementary information and complex dependencies across heterogeneous omics layers, resulting in suboptimal performance and limited generalizability in cancer classification tasks.

**Results:**

Here, we propose GONet, a hierarchical GNN framework that integrates omics expression profiles, including mRNA, miRNA, and protein data, with molecular interaction networks, where nodes represent omics features and edges represent molecular interactions. GONet learns global omics representations via an autoencoder, captures local structural dependencies using Deep Graph Infomax (DGI), and enhances omics-specific representations through an attention mechanism. A gated fusion module presentations through an attention mechanism. A further enables adaptive integration of heterogeneous features, improving cancer subtype and stage classification. GONet was systematically evaluated on 13 publicly available benchmark datasets used in prior studies, covering 10 cancer types, ensuring fair comparison across methods. Across mRNA and miRNA datasets, GONet achieved an average AUROC of 0.9262, outperforming representative GNN- and non-GNN-based methods by 13.77 and 6.35%, respectively. On an independent CPTAC cohort spanning 7 cancer types, it improved performance by 25.22 and 13.91%, respectively. Incorporating proteomics further improved cancer stage classification by 5.56%, demonstrating complementary predictive value. Ablation studies confirmed the effectiveness of the DGI-based module.

**Conclusions:**

Overall, GONet provides a unified framework for biologically informed multi-omics graph representation learning and cancer classification.

## Introduction

Cancer remains one of the leading causes of morbidity and mortality worldwide [1], driven by highly complex and heterogeneous molecular mechanisms. Elucidating these mechanisms is essential for understanding cancer initiation and progression, as well as for improving clinical diagnosis, treatment, and prevention [2, 3]. The advent of high-throughput omics technologies has enabled systematic characterization of cancer across multiple molecular layers [4, 5]. Genomics reveals the landscape of genetic alterations underlying cancer development [4, 6], while transcriptomics and proteomics provide gene expression patterns, including gene expression dynamics, post-translational modifications, and molecular interactions [4, 7, 8]. In essence, cancer initiation and development are cumulative results of genetic mutations [9]; therefore, the pronounced heterogeneity of cancers is likely associated with the interactions across networks involving genome, transcriptome, and proteome [4, 10]. Although single-omics data provide rich experimental evidences and bioinformatic insights at the molecular level, from the perspective of the central dogma [11], interactions across different molecular levels during the transmission of genetic information also influence biological phenotypes [12, 13]. Consequently, integrating multi-omics information is essential for a comprehensive characterization of cancer biology [5, 14, 15]. With continuous accumulation of large-scale resources, such as The Cancer Genome Atlas (TCGA) [3], and the Clinical Proteomic Tumor Analysis Consortium (CPTAC) [2], researchers have increasingly focused on multi-omics integration studies.

Benefiting from strong capabilities in nonlinear modeling and representation learning, deep learning has become a major methodology for multi-omics integration in cancer analysis [16–19]. Early deep learning approaches that primarily rely on non-graph neural network (GNN) architectures, such as autoencoders (AEs), multilayer perceptrons (MLPs), and attention-based models, transform different omics profiles into unified representations and alleviate problems of high dimensionality and noise. For example, MOCAT is an AE-based framework that integrates mRNA, miRNA, and DNA methylation data across multiple diseases, including Alzheimer’s disease and several cancer types, achieving improved classification performance in disease diagnosis, subtype, and stage prediction [20]. Attention-based models have been introduced to enhance multi-omics representation learning by dynamically weighting contributions from different omics features. Methods such as HTML and CLCLSA combine attention mechanisms with contrastive learning strategies and achieve well-improved performance in multi-cancer classification tasks [21, 22]. GNNs, another important class of deep learning models, are designed to capture relational dependencies in graph-structured data through message passing between nodes [23–25]. Early GNN-based multi-omics approaches are primarily constructed using sample-level similarity graphs for classification tasks, in which nodes represent individual samples and edges encode pairwise relationships derived from omics features. For instance, MOGONET employs graph convolutional networks (GCNs) [26] on sample similarity graphs constructed from mRNA, miRNA, and DNA methylation data for prediction of cancer subtypes/stages [27]. MOGCN constructs sample similarity graphs from CNV, mRNA, and RPPA data in TCGA breast cancer and was applied to subtype classification [28]. However, those approaches, either traditional non-GNN or sample-similarity-based GNN models, focus on learning global representations at the sample level from the gene expression matrices to integrate omics data. As a result, they cannot utilize the interaction information and are limited in capturing complex nonlinear dependencies across omics features, thereby leaving substantial room for improvement in representation learning for disease and cancer subtype prediction.

Cancer is not solely driven by single-gene aberrations but is regulated by complex molecular interaction networks. If interaction graphs are integrated into GNN-based multi-omics integration frameworks, with nodes representing gene features and edges characterizing the feature interactions, such a design is expected to enable GNNs to learn biologically local representations from molecular interaction topology and improve disease prediction [10, 16, 29]. Recently, SynOmics has extended GCN-based [26] architectures to intra- and inter-omic interaction graphs for cancer prognosis prediction [30], while Li et al. proposed a hybrid model to improve prediction performance for breast cancer subtype classification [31], in which MLPs encode global omics profiles and GCN or GAT [32] models molecular interaction networks. Nevertheless, there remains large room for improvement in current GNN-based models. First, feature extraction is insufficient across multiple levels. MLP-based encoders tend to compress high-dimensional omics data toward task-specific representations, potentially losing informative variations. Standard message-passing in GNNs is largely confined to local neighborhoods, and may therefore have limited ability to capture broader dependencies. In addition, most current methods assume homogeneous node representations, whereas they ignore intrinsic heterogeneity across omics types such as mRNA and proteins. Therefore, a novel feature extractor is required to be capable of representing both omics characteristics and interaction patterns. Second, representation fusion remains insufficiently coordinated. Since simple concatenation may lead to unstable hierarchical representations and weak discrimination power in downstream tasks, a new fusion mechanism that can resolve conflicts and inconsistencies among multiple representations is highly desirable. Third, with the growing proteomic evidence for cancer classification, a promising avenue is to introduce proteomics and the corresponding interaction networks into GNN-based models to enhance discriminative capacity [33–36].

In this study, the GONet is proposed, a novel multi-level GNN framework for cancer classification by integration of multi-omics profiles, mRNAs, miRNAs, and proteins, with interaction networks. GONet employs autoencoders to capture global representations, and applies Deep Graph Infomax (DGI) [37] to model local representations from molecular interaction networks. Considering the heterogeneous contribution across omics, GONet introduces an attention mechanism to generate omics representations that highlight differential regulation of gene expression. Furthermore, a gated fusion module is designed in GONet to dynamically reconcile global, local, and omics-specific representations, thereby alleviating potential conflicts and improving integration consistency. Results from benchmark tests and case studies suggest that GONet can improve predictive performance across several cancer datasets.

## Results

### GONet: a hierarchical graph neural network for multi-omics and molecular interaction integration

GONet is a modular, end-to-end framework that synergistically integrates matrix-based multi-omics data (e.g., transcriptomics and proteomics profiles) with graph-structured biomolecular interaction networks to derive biologically meaningful, low-dimensional representations for downstream classification tasks, including cancer staging and subtyping. Accordingly, the framework comprises 2 major parts: (1) dual-input: preprocessed multi-omics matrices and a curated interaction graph upon transcriptomes and proteomics; (2) model architecture: multi-level feature extraction followed by adaptive fusion; and downstream prediction classification. A schematic overview of framework is presented in Fig. 1.

**Figure 1:**
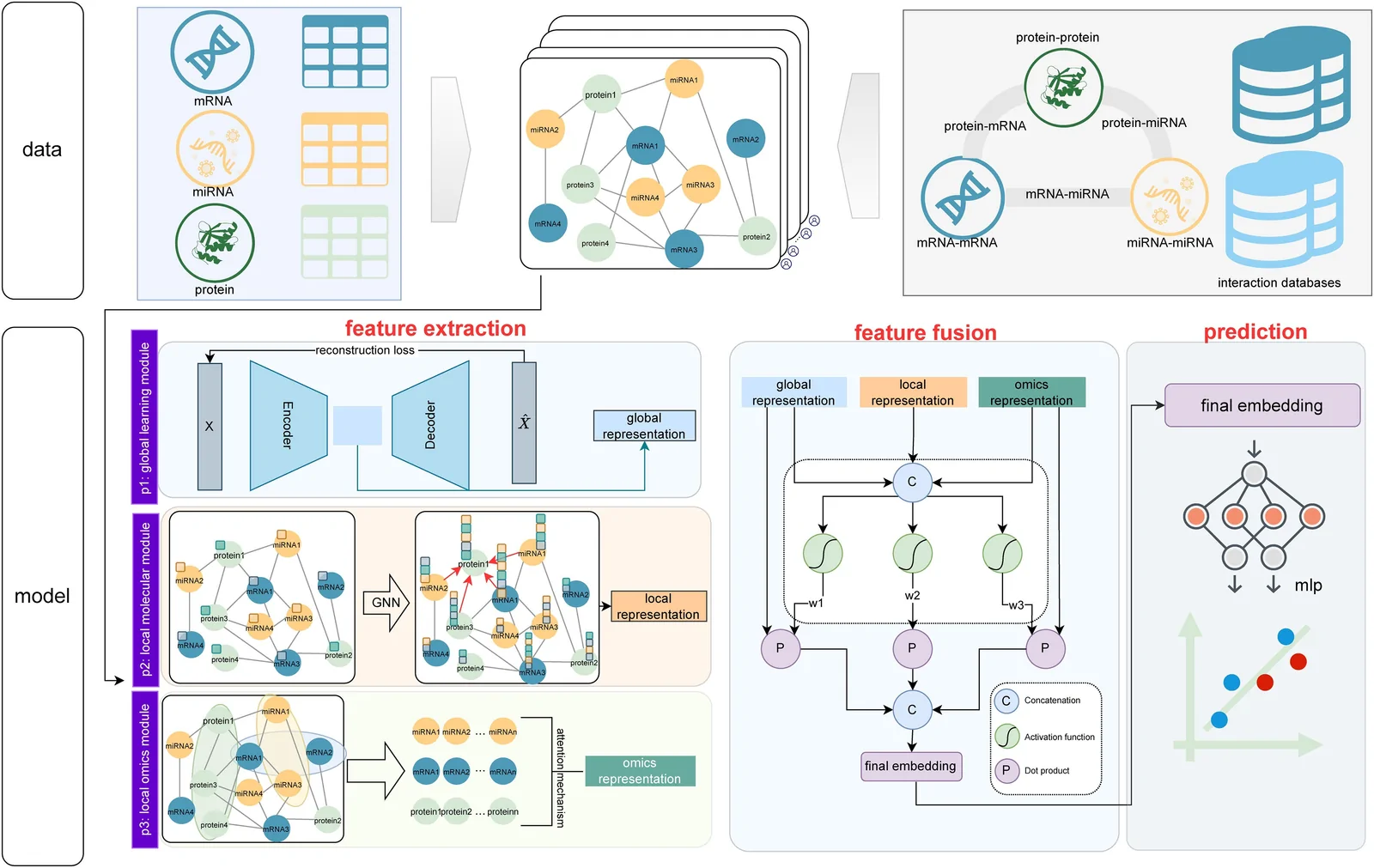
*Overview of the GONet framework*. GONet integrates multi-omics profiles (mRNA, miRNA, protein) with biomolecular interaction networks for cancer classification. *Data collection and graph construction*. Multi-omics datasets and curated molecular interaction networks are used to construct sample-specific molecular interaction graphs. *Model architecture*. Multi-omics matrices serve as inputs to the global learning module (AE-based), while the sample-specific molecular interaction graphs are processed by the local learning module (DGI-based). In parallel, the local omics module captures inter-omics dependencies using attention mechanism. The resulting representations are fused via feature fusion module (GFM-based) and passed to a prediction module for the downstream classification tasks.

In the dual-input part, multi-omics matrices, including mRNA, miRNA, and proteomics profiles, are preprocessed into a unified matrix $X\in {{R}^{n \times d}}$, where *n* and *d* represent the number of samples and features, respectively. For each sample, the personalized molecular interaction graph $G = ( {V,E,H} )$is constructed based on the curated interactions from public databases, in which *V* as nodes represents molecules, *E* as edges denotes interactions, and *H* stands for sample-specific molecular quantities (see the “Methods” section).

In the model architecture part, 3 core modules are employed, feature extraction, feature fusion, and prediction. In feature extraction modules: 3 synergistic sub-modules are designed to generate complementary data representations. (A) Global sample learning: regarding the widespread application of autoencoders for dimensionality reduction in multi-omics data integration and addressing the limitation of MLP-based compression in capturing information-rich global representations, AE is applied to compress the input matrix *X* into a low-dimensional representation ${{H}_{{\mathrm{ global}}}}$, while the decoder reconstructs the input $\hat{X}$ by minimizing mean squared error (MSE) loss. (B) Local molecular learning: considering global representations overlook local patterns in molecular interaction networks, and that conventional GCN and GAT models suffer from over-smoothing and limited discriminative capacity, DGI is applied to maximize node-graph mutual information via the contrastive loss, generating local representations ${{H}_{{\mathrm{ local}}}}$ from the molecular interaction graph *G*. (C) Local omics learning: addressing the lack of omics-specific information, a self-attention mechanism (SAM) [38] at omics-level adaptively weights inter-omics dependencies by learnable coefficients into ${{H}_{{\mathrm{ omics}}}}$. In feature fusion modules, an adaptive gated fusion mechanism (GFM) [39] aligns the complementary representations (${{H}_{{\mathrm{ global}}}},{{H}_{{\mathrm{ local}}}}$, and ${{H}_{{\mathrm{omics}}}}$) from the 3 sub-modules. The GFM adaptively weights and combines the 3 representations to generate a unified representation, ${{H}_{{\mathrm{ final}}}}$, allowing the model to adjust the relative contributions of the individual modules. In prediction modules, ${{H}_{{\mathrm{ final}}}}$ is passed through to a fully connected classifier, which outputs class probabilities for cancer subtype or stage prediction (Fig. 1).

### Performance evaluation of GONet modules compared with previous GNN-based methods

GNN-based methods have been applied to cancer type and stage prediction using multi-omics data in recent 5 years, including approaches with or without explicit modeling of interaction information, such as MOGONET [27], MOGCN [28], SynOmics [30], and Li’s method [31], MGDMCL [40], and MOGANet [41]. As shown in Fig. 2A, MOGONET and MOGCN exhibit inconsistent performance across datasets, often performing best on their original benchmark datasets. In contrast, SynOmics and Li’s method demonstrate relatively more stable cross-dataset performance, suggesting that interaction information may be beneficial for effective cancer classification. However, SynOmics primarily considered the contribution of molecular interactions while underutilizing sample-level information, whereas Li’s method integrates both local molecular interactions and global sample-level representations, yielding robust and generalizable multi-omics integration. Despite these advances, their representation learning and feature fusion strategies still leave room for further improvement.

**Figure 2:**
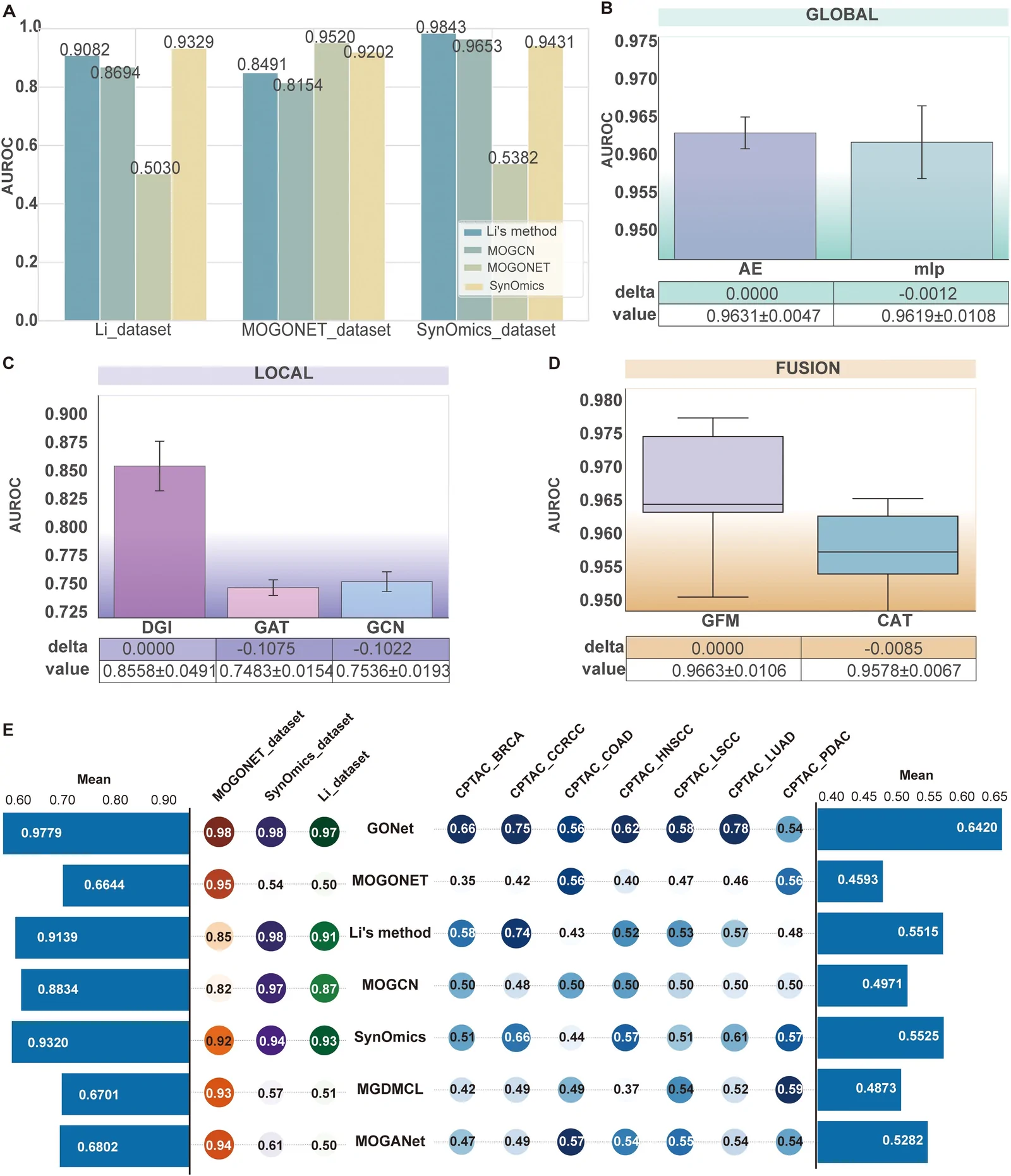
Module-level comparison and performance evaluation of GONet against existing GNN-based models. (A) Comparative predictive performance of representative GNN-based methods across multiple public multi-omics cancer datasets. (B–D) Module-specific evaluation of key architectural components in GONet on the BRCA subtype classification task using paired mRNA and miRNA data. (B) *Global learning module*. Performance comparison of AE in GONet with an MLP baseline. (C) *Local learning module*. Performance comparison of DGI with GCN and GAT baselines. (D) *Feature fusion module*. Comparison between the adaptive GFM used in GONet and CAT. (E) Generalization performance of GONet compared with representative GNN-based methods on original benchmark datasets (left) and CPTAC datasets (right).

To further analyze the contribution of each component, we compare GONet with Li et al.’s framework, which employs MLP-based global learning, GCN/GAT-based local learning, and concatenation-based fusion. In contrast, GONet adopts AE-based global encoding, DGI-based local learning, SAM-based omics modeling, and GFM-based fusion. Using the BRCA dataset from Li et al. (Li_dataset), all modules were evaluated independently under a unified classifier. For global learning, AE and MLP achieve comparable performance (AUROC: 0.9631±0.0047 vs. 0.9619±0.0108; Fig. 2B). For local learning, DGI significantly outperforms GCN and GAT (0.8558±0.0491 vs. 0.7536±0.0193 and 0.7483±0.0154; Fig. 2C). The SA-based omics module achieves an AUROC of 0.8077±0.039. For feature fusion, GFM slightly improves over concatenation (0.9663±0.0106 vs. 0.9578±0.0067; Fig. 2D). To further evaluate the contribution of each architectural component to entire GONet, ablation analyses were adopted on the CPTAC datasets, like removal of AE, replacement of DGI, removal of omics representation, and replacement of gated fusion. The ablation results endorsed the conclusions drawn from Fig. 2A–D, with DGI playing a role of major contributor. Detailed ablation results are provided in Supplementary Fig. S1A–D and Supplementary Tables S3 and S4. Overall, each module of GONet positively contributes to subtype classification, especially in local learning.

Performance of the full GONet framework was further evaluated for cancer subtype and stage classification in comparison with representative GNN-based methods on their original datasets using mRNA and miRNA data. As summarized in Fig. 2E, GONet was evaluated for cancer classification across the datasets used for the development of these GNN-based methods. On Li’s dataset, GONet achieved an AUROC of 0.9663, representing a 6.39% improvement over Li’s method (0.9082). On the MOGONET datasets, GONet yielded an AUROC of 0.9824, higher than those of MOGONET (0.9520), MGDMCL (0.9329), and MOGANet (0.9386), respectively. GONet also obtained an AUROC of 0.9849 on the SynOmics dataset, compared with 0.9431 for SynOmics. Together, these results demonstrate the improved prediction of cancer classification performance by GONet.

Next question is whether the prediction performance of GONet and the other GNN-based methods is comparable on the same omics datasets. CPTAC dataset was used for such evaluation, using mRNA and miRNA data across 7 cancer types (BRCA, CCRCC, COAD, HNSCC, LUAD, LSCC, and PDAC). As shown in Fig. 2E and Table S4, GONet consistently yielded higher prediction performance than other GNN-based methods for cancer stage classification across the CPTAC datasets, with an average AUROC of 0.6420, corresponding to 16.41% relative improvement over Li’s method, 39.78% over MOGONET, 29.15% over MOGCN, 16.20% over SynOmics, 21.54% over MOGANet (0.5282), and 31.75% over MGDMCL (0.4873) (*P* < 0.05). Although performance gains are consistent, absolute AUROC values remain moderate, suggesting that dataset size and omics quality remain key limiting factors for multi-omics cancer prediction.

### Proteomics contributes to GONet for cancer classification

Unlike mRNA and miRNA data, which primarily capture transcriptional information, proteomics data directly reflects the functional state of gene expression [33, 35, 42]. Besides, large-scale resources, such as BioGRID [43] and STRING [44], accumulate extensive protein–protein interaction (PPI) information, providing inherent support for GNN-based modeling. These characteristics motivate the evaluation of proteomics as an alternative omics modality within the GONet for cancer classification prediction.

To systematically assess the applicability of GONet to proteomics data, a modality substitution experiment was conducted, where transcriptomic inputs (miRNA and mRNA) were replaced with proteomics data together with corresponding PPI networks across 7 CPTAC cancer types.

As presented in Fig. 3A, GONet well accommodated the proteomics data, achieving performance comparable to transcriptomics-based models (average AUROC: 0.6360 vs. 0.6420). At the individual cancer-type level, the proteomics-based predictions achieved slightly higher AUROC values than transcriptomics-based predictions in 5 of 7 cancer types (BRCA, CCRCC, LUAD, HNSCC, and PDAC), whereas modestly lower performance was observed in COAD and LSCC. Overall, these results suggest that the proteomics data is a viable modality for GNN-based cancer stage prediction with GONet, at least in the CPTAC cohort.

**Figure 3:**
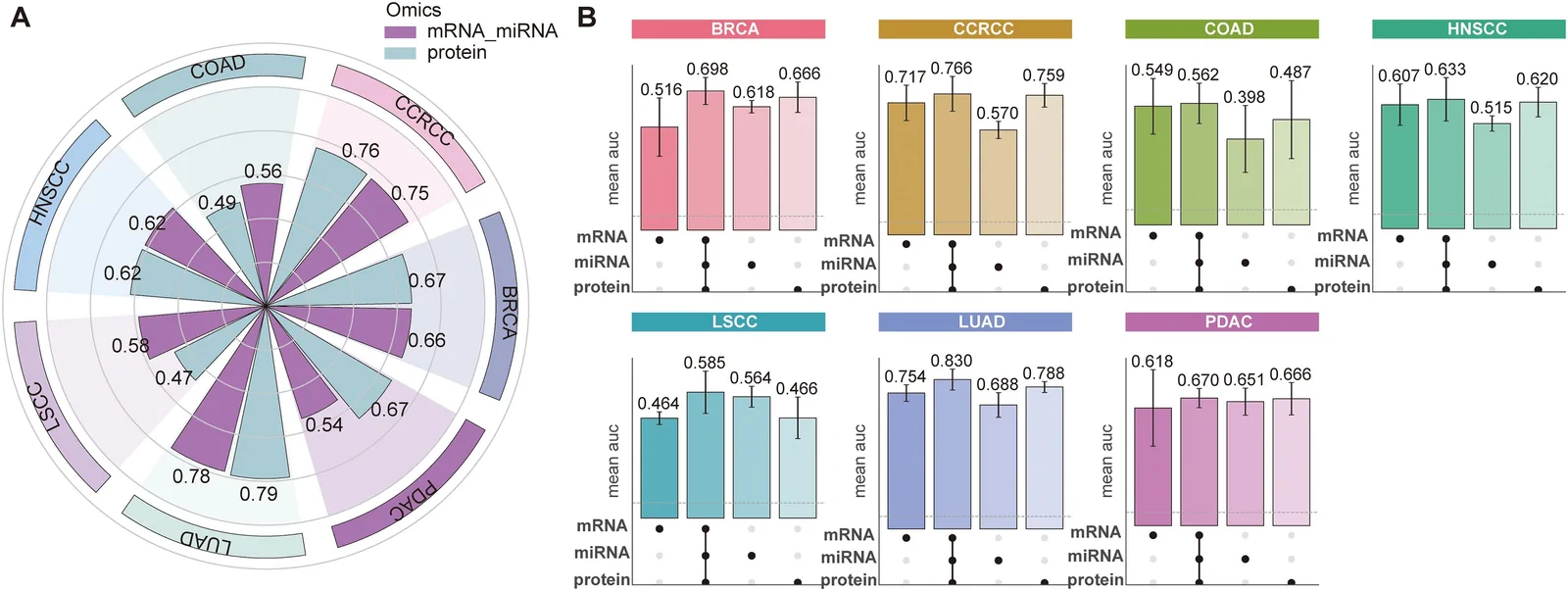
Evaluation of proteomics integration in GONet. (A) Comparison of transcriptomics-based (mRNA-miRNA) and proteomics-based inputs for cancer stage classification across CPTAC cohort. (B) Performance of single- and tri-omics integration (mRNA, miRNA, protein) for cancer stage classification across CPTAC datasets.

An arising question concerns the relative contribution of individual omics to cancer stage prediction in GONet. As summarized in Fig. 3B, proteomics alone yielded higher predictive performance among the 3 single-omics inputs, with an average AUROC of 0.6360, compared to 0.6038 for mRNA and 0.5721 for miRNA. At the individual dataset, proteomics-based predictions outperformed those derived from either mRNA or miRNA alone in 5 of the 7 CPTAC cancer types. These results suggest that proteomics provides stronger discriminative information for cancer stage classification than mRNA or miRNA alone within the GONet framework.

Finally, we evaluated whether the integration of 3 omics data could further improve cancer stage prediction in CPTAC. As shown in Fig. 3B, the mean AUROC for tri-omics integration of miRNA, mRNA, and proteomics in GONet was 0.6777, which is obviously higher than that derived by proteomics alone (0.6360; *P* < 0.05) or transcriptomics (miRNA-mRNA:0.6420; *P* < 0.05). These results demonstrate that multi-omics integration provides complementary information, with proteomics contributing essential functional-level signals that enhance cancer stage prediction.

### Performance comparison of GONet and non-GNN methods for cancer classification

A wide range of non-GNN-based multi-omics integration methods have been applied to cancer type and stage classification, including several conventional methods, such as RF [45], SVM [46], LR [47], AE, MLP, and recently published approaches, such as HTML [22], CLCLSA [21], and MOAEAM [48]. Given the strong performance of GONet compared with existing GNN-based methods, a systematic evaluation was conducted from 3 perspectives: (i) comparison with existing non-GNN methods on their original datasets and on CPTAC, (ii) evaluation under proteomics integration, and (iii) global statistical comparison across all datasets and methods.

A total of 13 datasets were recruited, including those previously studied by HTML (COAD, ESCA, GBMLGG, SARC, STAD, STES, THCA, UCEC), CLCLSA (LGG, KIPAN), MOGONET, SynOmics, and Li et al. (Table S1). All datasets contained the mRNA and miRNA profiles with cancer stage or subtype labels. Fourteen approaches, including GNN- and non-GNN-based methods, were applied onto each dataset, with results summarized in Supplementary Data (Table S1). The performance can be categorized into 3 circumstances, (1) due to unclear reason, all approaches achieved relatively poor performance on the 2 datasets HTML_GBMLGG and HTML_STAD, with AUROC ranging from 0.6297 to 0.7272 (Fig. 4A); (2) most approaches obtained near-saturated predictions on 3 datasets HTML_ESCA, HTML_STES, and HTML_KIPAN, with AUROC values exceeding 0.99 (Fig. 4B); and (3) the different approaches exhibited variable prediction performance on the remaining 8 datasets (Fig. 4C). GONet consistently showed higher prediction performance than the comparison methods, with an average AUROC of 0.9495 versus 0.8687 for the non-GNN methods. Notably, the GNN-based comparison methods showed lower prediction performance on the same datasets, with an average AUROC of 0.7465. These results strengthen our argument that improvements in representation learning and interaction modeling are critical for GNN-based multi-omics methods.

**Figure 4:**
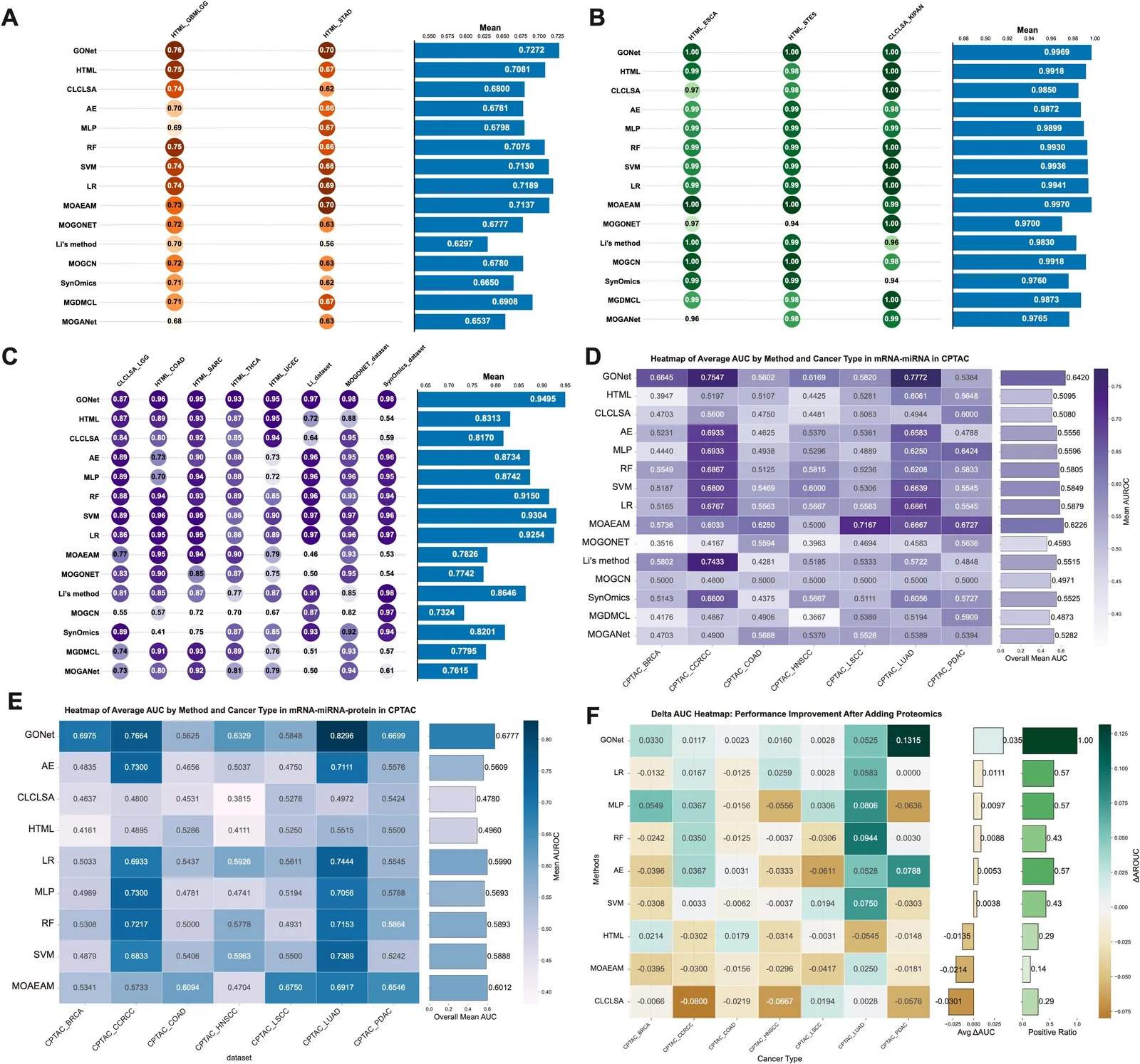
Systematic benchmarking of GONet performance across multi-omics cancer classification tasks. (A–C) Performance comparison for bi-omics integration (mRNA-miRNA) between GONet and 14 competing methods across 13 datasets collected from previous studies grouped by task difficulty. (D) Heatmap depicting AUROC values for bi-omics integration (mRNA-miRNA) between GONet and the 14 competing methods across 7 CPTAC cancers. (E) Heatmap showing AUROC values for tri-omics integration (mRNA, miRNA, and proteomics) between GONet and the 8 non-GNN methods across 7 CPTAC cancers. (F) Heatmap illustrating the absolute AUROC improvements (ΔAUROC) for each method after integrating proteomics data.

All the non-GNN methods were applied to the CPTAC datasets for assessment of cancer stage classification. As shown in Fig. 4D, these methods achieve AUROC values ranging from 0.5080 to 0.6226, all significantly lower than GONet (0.6420; *P* < 0.05). Although overall performance remains moderate across all methods, likely due to limited sample size and task difficulty, GONet consistently achieves the best performance among both GNN and non-GNN approaches.

Giving the importance of proteomics, a question is raised if non-GNN-based approaches can effectively utilize tri-omics integration (mRNA, miRNA, and proteomics) on CPTAC. As shown in Fig. 4E, non-GNN-based approaches exhibited lower predictive performance, with average AUROC values spanning 0.4780–0.6012 (*P* < 0.05). Moreover, proteomics integration led to inconsistent gains across methods and across the 7 cancer types (Fig. 4F).

Finally, a Friedman test across 27 experimental scenarios was conducted to statistically evaluate the advantages of GONet. As shown in Supplementary Fig. S5, GONet ranked first, and the null hypothesis of equal performance was rejected (*P* < 0.001), indicating that GONet achieves statistically differences in the evaluation scenarios.

## Discussion

GNNs have recently emerged as a powerful approach for multi-omics data integration by incorporating molecular interaction networks, as exemplified by Li et al. and the SynOmics framework [30, 31]. However, as shown in Fig. 2A, systematic comparison of the current GNN-based approaches reveals that integration of interactions does not consistently yield improve performance, suggesting that effectiveness depends not only on the introduction of interaction information, but also on how efficiently they are leveraged. In complex cancer omics networks, conventional GCN and GAT models inherently behave as low-pass filters [49, 50], aggregating local neighborhoods information, and readily suffering from over-smoothing during propagation processes, resulting in indistinguishable node representations and reduced discriminability [50, 51]. To avoid the drawback, GONet innovatively incorporates DGI, which extracts more discriminative node representations from molecular interaction networks by maximizing mutual information between node-level representations and graph-level representations [37]. From an information-theoretic perspective, mutual information maximization captures global context and higher-order dependencies, and has been shown to improve embedding quality for downstream tasks [52, 53]. Furthermore, DGI’s contrastive learning framework distinguishes real node-graph pairs from corrupted ones, encouraging robustness to noise and improving representation quality [54, 55]. Module comparison experiments confirm that the DGI markedly improves the standard GCN or GAT, yielding superior classification performance (Fig. 2C). The improvement is consistent with prior reports in biological network representation learning, in which DGI enhances representation quality and downstream task performance in molecular-disease association networks [56], PPI networks [57], and single-cell transcriptome-derived gene regulatory networks [58].

Proteins represent functional executors of biological processes, whereas transcripts primarily reflect upstream regulatory activity [59]. Accordingly, proteomics provides complementary functional and interaction-level signals, yet remains underutilized in existing approaches. Some studies integrated only protein expression data (MOGCN with RPPA profiles [28]), while others included protein interaction networks without protein abundance, leaving synergistic modeling unexplored [31]. In this study, we simultaneously integrated protein abundance and associated interaction networks from the CPTAC cohort. Proteomes alone exhibited predictive power comparable to mRNA and miRNA, while integration with transcriptomic data further improves performance (Figs 3A, B and 4C). Ablation analysis shows that neither abundance-only nor interaction-only signals yield strong performance (AUROC = 0.6039 and 0.5587), whereas their combination yields a substantial gain (AUROC = 0.6777; Fig. S7). These results indicate that predictive improvements arise from the synergistic integration of proteomic abundance and interaction structure. Notably, although incorporation of proteome into GONet improves performance, the gain magnitude remains moderate. GONet still leaves substantial room for further improvement, primarily in 2 directions: more advanced model architectures, such as large language model-based approaches [60, 61], and larger cohort sizes to enhance deep learning capacity and better capture mRNA and protein characteristics [62].

Beyond predictive performance, exploring the relationships between GONet-based disease classification and molecular features may provide additional insights into the biological relevance of the predictions. As pointed out by Sundararajan et al., Integrated Gradients (IG) can be used to quantify the contribution of individual input features to model predictions and facilitate the identification of biologically relevant signals [63]. Further development of such interpretability analyses may provide additional biological insights into GONet-based disease classification.

In summary, a modular GNN-based framework for integration of omics data with molecular interactions, GONet, was proposed and examined its capacity in cancer types and stages classification. GONet adopts a hierarchical representation learning strategy, AE-based global encoding, DGI-based local interaction modeling, attention-based modality weighting, and gated multi-level fusion. Extensive evaluations across multiple cancer cohorts and omics settings demonstrate that GONet achieves consistently robust performance compared with both GNN- and non-GNN-based methods. Particularly, GONet enables systematic joint modeling of proteomic abundance and interaction topology, with stable performance gains when proteomics data are involved. Overall, GONet has established a multi-omics integration framework that potentially provides a methodological foundation for disease classification.

## Methods

### Data preprocessing

The benchmark datasets followed the original formats in previous literatures, whereas the CPTAC data were processed using the procedures described by Wang et al. and Ji et al. [27, 65]. Specifically, data preprocessing procedure followed a step-wise strategy in each random train-test split, removal of features with more than 20% missing values, imputation of remaining missing values using *k*-nearest neighbors (KNN), min–max normalization, and ANOVA-based feature selection were performed on the training set. And then processing procedure and selected features were applied to the corresponding independent test sets.

### Molecular interaction networks

Comprehensive molecular interaction networks in this study were curated by aggregation of public interaction repositories, including PPIs derived from BioGRID [43], STRING [44], and IID [66]; mRNA-related regulatory interactions sourced from RISE [67], NPInter [68], and RNAInter [69]; and miRNA-related regulatory interactions retrieved from miRNet [70]. All interaction data were limited to human interactions, and nodes and edges involved in such interactions were further filtered to remove incomplete or erroneous records (Table S3). Molecular identifiers were standardized using UniProt IDs for proteins, HGNC symbols for mRNAs, and miRDB IDs for miRNAs.

### Graph construction based on multi-omics interactions

For each patient sample *s*, a personalized molecular interaction graph ${{G}_s}$ was constructed by integrating multi-omics profiles with curated interaction networks and defined as


\begin{eqnarray*}
{{G}_s} = \left( {{{V}_s},{{E}_s},{{H}_s}} \right),
\end{eqnarray*}

where ${{V}_s}$ represents nodes with molecular entities (mRNAs, miRNAs, and proteins) detected in sample *s*, with ${{v}_i}$ and ${{v}_j}$ correspond to 2 nodes of ${{V}_s}.{\mathrm{\ }}{{E}_s}$ denotes edges derived from the curated interaction networks for sample *s*. Each edge ${{e}_{ij}}\in {{E}_s}$ between molecules ${{v}_i}$ and ${{v}_j}$ is defined as


\begin{eqnarray*}
{{e}_{ij}} = \left\{ {\begin{array}{@{}*{2}{c}@{}} 1, & \mathrm{ if}\, {\rm an}\ \mathrm{ interaction}\ {\rm exists}\ \mathrm{ between}\ v_i\ {\rm and}\ v_j\\{0}, & \mathrm{ otherwise} \end{array}} \right.
\end{eqnarray*}

${{H}_s}$ corresponds to the normalized expression values of the respective molecular entities in sample *s*.

### Construction of GONet

#### Global sample learning module

The AE used for global sample learning comprises an encoder and decoder.

**Encoder processing**. Let the original multi-omics feature matrix $X\in {{R}^{n \times m}}$ serve as input, where *n* represents the number of samples and *m* denotes the total number of molecular entities. A low dimensional global representation, ${{H}_{{\mathrm{ global}}}}\in {{R}^{n \times {{d}_g}}}({{d}_g} \ll m)$, is compressed by encoder functioned ${{f}_{enc}}( \cdot )$:


\begin{eqnarray*}
{{H}_{{\mathrm{ global}}}} = {{f}_{\mathrm{ enc}}}\left( X \right),
\end{eqnarray*}

where the ${{f}_{\mathrm{ enc}}}$ consists of fully connected layers followed by LeakyReLU activations to capture non-linear patterns.

**Decoder processing**. The reconstructed representation $\hat{X}$ is generated from ${{H}_{{\mathrm{ global}}}}$ by decoder functioned ${{f}_{\mathrm{ dec}}}( \cdot )$ to ensure that the compressed ${{H}_{{\mathrm{ global}}}}$ retains the essential information of *X*. The reconstruction process is formulated as


\begin{eqnarray*}
\hat{X} = {{f}_{\mathrm{ dec}}}\left( {{{H}_{{\mathrm{ global}}}}} \right),
\end{eqnarray*}

where $\hat{X}\in {{R}^{n \times m}}$ represents the approximation of original matrix *X*. The decoder maintains a symmetric architecture with the encoder.

**Reconstruction loss**. The AE parameters are optimized by minimizing reconstruction loss ${{L}_{\mathrm{ rec}}}$ between *X* and $\hat{X}$, defined as


\begin{eqnarray*}
{{L}_{\mathrm{ rec}}} = \parallel X - \hat{X}\parallel _2^2,
\end{eqnarray*}

where $\parallel {\mathrm{\ }}\parallel _2^2{\mathrm{\ }}$is defined as the MSE.

#### Local molecular learning module

DGI model for local molecular learning is applied to learn high-quality local representations at 2 hierarchical scales: the node-level (individual molecules) and the graph-level (the entire molecular interaction network).

**Node-level representation**. For each sample *s*, node-level representations ${{H}_{{\mathrm{ node}}}}\in {{R}^{| {{{V}_s}} | \times {{d}_l}}}$ are learned via graph convolutional encoder $\mathrm{ GCN}( \cdot )$ that aggregates information from neighboring molecules in the sample-specific molecular interaction network ${{G}_s}$:


\begin{eqnarray*}
{{H}_{{\mathrm{ node}}}} = \mathrm{ GCN}\left( {{{G}_s}} \right),
\end{eqnarray*}

where $| {{{V}_s}} |$ represents the number of molecule nodes in sample s, and ${{d}_l}$ denotes the dimensionality of node representation. Each row ${{h}_v}\in {{H}_{{\mathrm{ node}}}}$ corresponds to a ${{d}_l}$-dimensional embedding of molecule *v*.

**Graph-level representation**. The graph-level representation ${{H}_{{\mathrm{ local}}}}\in {{R}^{2{{d}_l}}}$ is obtained by aggregating ${{H}_{{\mathrm{ node}}}}$ with a dual-pooling strategy (mean and max pooling) to capture the global topology of the molecular network:


\begin{eqnarray*}
H_{\mathrm{ local}} = \left[\frac {1}{\left| V_s \right|} \sum_{v\in V_s} vh_v \right]\oplus \left[\max_{v\in V_s} h_v \right],
\end{eqnarray*}

where *⊕* denotes vector concatenations, and the left and right part indicate pooling strategies to generate $2{{d}_l}$-dimensional vector for sample *s*.

**Maximization of mutual information**. To ensure that node-level representations encode global topology information, a discriminator *D* is introduced to maximize the mutual information between ${{H}_{{\mathrm{ node}}}}$ and ${{H}_{{\mathrm{ local}}}}$, which distinguishes between real node-graph pairs $( {{{h}_v},{{H}_{{\mathrm{ local}}}}} )$ from the true interaction graph ${{G}_s}$, and corrupted pairs $( {{{{\tilde{h}}}_v},{{H}_{{\mathrm{ local}}}}} )$, as described below:


\begin{eqnarray*}
L_{\mathrm{ DGI}} = - \frac{1}{\left| V_s \right|} \sum_{v\in V_s} \left[\mathrm{ log}\ D(h_v,\ H_{\mathrm{ local}}) + \mathrm{ log} \left(1-D\left(\widetilde{h_v},H_{\mathrm{ local}}\right)\right)\right],
\end{eqnarray*}

where ${{\tilde{h}}_v}$ represents the corrupted node representation obtained from a corrupted graph with randomly shuffle nodes.

#### Local omics learning module

The local omics learning module aggregates node-level representations into omics-specific representations and integrates information from different omics through an adaptive attention mechanism.

**Omics-specific representation aggregation**. To reduce noise and capture omics-specific signatures, omics-specific representations ${{H}_m}$ for each omics modality *m* are generated by averaging the node-level representations of its constituent molecules ${{V}_m}$:


\begin{eqnarray*}
{{H}_m} = \frac{1}{{\left| {{{V}_m}} \right|}}\mathop \sum \limits_{v\in {{V}_m}} {{h}_v},
\end{eqnarray*}

where ${{V}_m}$ represents the set of nodes belonging to modality *m*. The representations of all omics modalities are then stacked as


\begin{eqnarray*}
{{H}_{{\mathrm{ stack}}}} = \left[ {\begin{array}{@{}*{1}{c}@{}} {{{H}_1}}\\ {{{H}_2}}\\\vdots \\ {{{H}_{\left| M \right|}}} \end{array}} \right],\qquad {{H}_{{\mathrm{ stack}}}}\in {{R}^{\left| M \right| \times {{d}_l}}},
\end{eqnarray*}

where *| M |* is the number of omics.

**Self-attention mechanism**. To adaptively weight the contributions of different omics modalities, the attention weights *A* are computed as follows:


\begin{eqnarray*}
A = {\mathrm{ softmax}}\left( {\frac{{\sigma \left( {{{H}_{{\mathrm{ stack}}}}{{W}_Q}} \right){{{\left( {{{H}_{{\mathrm{ stack}}}}{{W}_k}} \right)}}^ \top }}}{{\sqrt {{{d}_k}} }}} \right),
\end{eqnarray*}

where ${{W}_Q}$ and ${{W}_K}$ are learnable weight matrices and ${{d}_k}$ is the attention dimension. The fused omics-level representation ${{H}_{{\mathrm{ omics}}}}$ is obtained by weighting ${{H}_{{\mathrm{ stack}}}}$ with A:


\begin{eqnarray*}
{{H}_{{\mathrm{ omics}}}} = A\left( {{{H}_{{\mathrm{ stack}}}}{{W}_V}} \right).
\end{eqnarray*}

**Contrastive loss**. To optimize the omics module and ensure that the learned representations ${{H}_{{\mathrm{omics}}}}$ are discriminative, a supervised contrastive loss ${{L}_{\mathrm{ sup}}}$ is minimized to encourage sample with same label *y* to have similar embeddings:


\begin{eqnarray*}
{{L}_{\mathrm{ sup}}} = {\mathrm{ SupConLoss}}\left( {{{H}_{{\mathrm{ omics}}}},y;\tau } \right),
\end{eqnarray*}

where ${\mathrm{\tau }}$ represents the temperature hyperparameter controlling the scale of the loss.

#### Feature fusion module

The feature fusion module integrates global, local, and omics representations through representation alignment followed by adaptive fusion to synthesize multi-level information.

**Representation alignment**. Given the different feature spaces and dimensions of ${{H}_{{\mathrm{ global}}}}{\mathrm{\ }}{{H}_{{\mathrm{ local}}}}$ and ${{H}_{{\mathrm{ omics}}}}$, the 3 representations are projected into aligned representations ${{{{Z}}}_{\mathrm{ mod}}}$ with a common dimension ${{d}_f}$ by a fully connected layer followed by a LeakyReLU activation:


\begin{eqnarray*}
{{Z}_{\mathrm{ mod}}} &=& {\mathrm{ LeakyReLU}}\left( {{{W}_{\mathrm{ mod}}}{{H}_{\mathrm{ mod}}} + {{b}_{\mathrm{ mod}}}} \right),\\ \mathrm{ mod} &\in& \left\{ {{\mathrm{ global}},{\mathrm{ local}},{\mathrm{ omics}}} \right\},
\end{eqnarray*}

where ${{W}_{\mathrm{ mod}}}{\mathrm{\ }}$ and ${{b}_{\mathrm{ mod}}}$ are learnable parameters.

**Representation fusion**. An adaptive gated mechanism ${{f}_{{\mathrm{Gate}}}}( \cdot )$ is employed to learn relative importance weights ${{\alpha }_{\mathrm{ mod}}}$ of each representation:


\begin{eqnarray*}
{{\alpha }_{\mathrm{ mod}}} = \left[ {{{\alpha }_{{\mathrm{ global}}}},{{\alpha }_{{\mathrm{ local}}}},{{\alpha }_{{\mathrm{ omics}}}}} \right] = {{f}_{{\mathrm{Gate}}}}\left( {\left[ {{{{{Z}}}_{{\mathrm{ global}}}}\oplus{{{{Z}}}_{{\mathrm{ local}}}}\oplus{{{{Z}}}_{{\mathrm{ omics}}}}} \right]} \right),
\end{eqnarray*}

\begin{eqnarray*}
\mathop \sum \limits_{{\mathrm{mod}}} {{\alpha }_{\mathrm{ mod}}} = 1,
\end{eqnarray*}

where $\mathrm{ oplus}$ denotes concatenation, and the gating function is implemented as a softmax operation to normalize the importance weights across the 3 modules. The final fused representation ${{H}_{{\mathrm{ final}}}}$ is defined as the convex combination of the ${{Z}_{\mathrm{ mod}}}$:


\begin{eqnarray*}
{{H}_{{\mathrm{ final}}}} = \mathop \sum \limits_{{\mathrm{mod}}} {{\alpha }_{\mathrm{ mod}}}{{Z}_{\mathrm{ mod}}},\ {{H}_{{\mathrm{final}}}} \in {{R}^{n \times {{d}_f}}}.
\end{eqnarray*}

#### Prediction module

The prediction module performs cancer stage or subtype classification based on ${{H}_{{\mathrm{ final}}}}$, and the prediction performance is assessed using focal loss [71].

**Classification prediction**. The fused representation ${{H}_{{\mathrm{ final}}}}$ is fed into a fully connected layer with learnable parameters ${{W}_c}$ and ${{b}_c}$, followed by a $\mathrm{ softmax}$ function to predict cancer stages or subtypes. The predicted probability vector *p* is computed as


\begin{eqnarray*}
p = {\mathrm{ softmax}}\left( {{{W}_c}{{H}_{{\mathrm{ final}}}} + {{b}_c}} \right).
\end{eqnarray*}

**Focal loss**. Focal loss is used as the classification loss ${{L}_{\mathrm{ cls}}}$, which measures discrepancy between the predicted probability *p* and the true class $y\ \in \{ {1,{\mathrm{\ }}\ldots ,\ C} \}$:


\begin{eqnarray*}
{{L}_{\mathrm{ cls}}} = - \alpha {{\left( {1 - {{p}_y}} \right)}^\gamma }\mathrm{ log}\left( {{{p}_y}} \right),
\end{eqnarray*}

where ${{p}_y}$ represents the predicted probability for true label *y*, and ${\mathrm{\alpha }}$ and ${\mathrm{\gamma }}$ are hyperparameter controlling class weighting and focusing strength, respectively.

#### Total loss function

The GONet framework is trained by minimizing a joint multi-objective loss function:


\begin{eqnarray*}
{{L}_{{\mathrm{ total}}}} = {{\lambda }_{\mathrm{ rec}}}{{L}_{\mathrm{ rec}}} + {{\lambda }_{\mathrm{ DGI}}}{{L}_{\mathrm{ DGI}}} + {{\lambda }_{\mathrm{ sup}}}{{L}_{\mathrm{ sup}}} + {{\lambda }_{\mathrm{ cls}}}{{L}_{\mathrm{ cls}}} + {{\lambda }_{\mathrm{ reg}}}{{L}_{\mathrm{ reg}}},
\end{eqnarray*}

where ${{L}_{\mathrm{ reg}}}$ is a regularization item to prevent overfitting, and trade-off hyperparameters {${{{\mathrm{\lambda }}}_{{\mathrm{rec}}}},{{{\mathrm{\lambda }}}_{{\mathrm{DGI}}}},{{{\mathrm{\lambda }}}_{{\mathrm{contra}}}},{{{\mathrm{\lambda }}}_{{\mathrm{cls}}}},{\mathrm{and}}\ {{{\mathrm{\lambda }}}_{{\mathrm{reg}}}}$} control the relative contribution of each loss items.

#### Data splitting and model training

Each dataset was partitioned into a training set and an independent test set at an 8:2 ratio using stratified random splitting. For GONet, hyperparameters were optimized strictly in the 80% training set using 5-fold cross-validation. Based on the optimal hyperparameters determined via cross-validation, the final model was retrained on the entire 80% training set and subsequently evaluated on the held-out 20% independent test set. To assess the stability of GONet, the entire procedure was repeated 5 times using different random seeds, and the final performance metrics were reported as the average across these 5 iterations. All comparison methods, regardless of GNN or non-GNN, were evaluated using the same train-test splits to ensure a fair and consistent benchmarking comparison (Supplementary Fig. S3). All experiments were performed on a Linux system equipped with an NVIDIA A100 GPU (80 GB VRAM). The GONet was implemented in Python 3.8, using PyTorch 2.0 with CUDA 11.8, and PyTorch Geometric (PyG) 2.4.0.

#### Hyperparameter optimization

The hyperparameters of GONet were optimized on the training set using Optuna [72], a framework based on Bayesian optimization principles. Specifically, the optimization was conducted within a predefined search space through many independent trials. In each trial, Optuna proposed a candidate hyperparameter configuration based on the results of previous trials, which was then evaluated using 5-fold cross-validation. The mean AUROC across the 5 folds was used as the performance metric to guide selection of the next candidate configuration of hyperparameters. After completing the optimization process, the hyperparameters configuration yielding the highest mean AUROC was selected as the final configuration for GONet (see Tables S5 and S6).

## Additional files

**Supplementary Figure S1**. Module ablation analyses of GONet for cancer stage classification. To systematically quantify the contribution of each architectural component in GONet, module-wise ablation analyses were conducted on the cancer stage classification task using paired mRNA, miRNA, and proteomics data from 7 cancer types in the CPTAC cohort. (A) *Ablation of the global sample module:* Performance of the full GONet model was compared with a variant without the AE-based global sample learning (noAE). (B) *Ablation of local molecular module*: DGI was replaced with graph convolutional network (GCN) and graph attention network (GAT), while keeping other GONet components unchanged. (C) *Ablation of omics module*: The full GONet model was compared with a variant lacking the self-attention-based omics modeling (noOmics). (D) *Ablation of feature fusion strategy*: The adaptive gated fusion mechanism in GONet was replaced with naive feature concatenation (CAT) for integrating representations across different modules. These module-wise ablation experiments collectively quantify the individual contribution of each component and validate the overall architectural design of GONet.

**Supplementary Figure S2**. Interpretability analysis of GONet predictions using Integrated Gradients (IG) in an independent Fu-iCCA cohort. (A) Receiver operating characteristic curve demonstrating the subtype classification performance of GONet in the independent Fu-iCCA cohort. GONet achieved robust classification performance with an AUROC of 0.98. (B–C) Distribution of Integrated Gradients (IG) scores for the top 100 features identified from the global and local modules, stratified by molecular modality (transcriptomic and proteomic features). The results demonstrate that highly ranked predictive features were derived from both mRNA and protein modalities. (D) Sankey diagram illustrating the functional associations of the top-ranked mRNA and protein features identified by GONet. The diagram depicts the relationships among molecular features, enriched biological functions, and corresponding Fu-iCCA molecular subtypes, highlighting the biological processes and signaling pathways associated with the identified predictive features. (E) Box plots showing the subtype-specific distribution patterns of the prioritized molecular features. The consistent differential expression patterns across Fu-iCCA subtypes further support the biological relevance of the features identified by GONet.

**Supplementary Figure S3**. Illustration of GONet hyperparameters and training strategy. Schematic diagram depicts the key hyperparameters and training procedures employed in GONet for multi-omics cancer classification tasks. (See the “Methods” section for full optimization and training details).

**Supplementary Figure S4**. Illustration of data preprocessing.

**Supplementary Figure S5**. Average ranks of all methods across multiple benchmarking scenarios, with lower ranks indicating superior overall performance.

**Supplementary Figure S6**. Performance gains from proteomics integration in GONet across single- and bi-omics baselines. The incremental impact of proteomics integration was assessed by measuring the change in AUROC (ΔAUROC) when proteomics data were added to baseline models. Analyses were conducted using paired data from 7 cancer types in the CPTAC datasets. (A) Single-omics mRNA baseline: ΔAUROC observed after adding proteomics. (B) Single-omics miRNA baseline: ΔAUROC observed after introducing proteomics. (C) Bi-omics (mRNA-miRNA) baseline: ΔAUROC observed after integrating proteomics. Box plots display distributions of ΔAUROC values, with mean values annotated. These analyses highlight the quantitative benefit of proteomics integration for improving multi-omics cancer classification in GONet.

**Table S1**. Characteristics of public multi-omics datasets used for model training and evaluation.

**Table S2**. Overview of curated molecular interaction networks used for graph construction.

**Table S3**. Ablation analysis of individual GONet modules for cancer classification performance.

**Table S4**. Quantitative summary of module-wise ablation analyses of GONet across 7 CPTAC cancer cohorts for cancer stage classification.

**Table S5**. Detailed hyperparameter space for the GONet model.

**Table S6**. Detailed hyperparameter configurations for the GONet model.

**Table S7**. The IG scores of global module and local module within the GONet.

## Availability of source code and requirements

Project name: GONet

Project home page: https://github.com/EayonYang/GONet.git

License: Apache-2.0 license

Operating system(s): Ubuntu 22.04

Programming language: Python 3.8 or higher

Other requirements: PyTorch 2.1 or higher, Pytorch Geometric 2.4 or higher, and other dependencies specified in the project repository

RRID: SCR_028640

## List of abbreviations

AE: AutoEncoder; AUROC: Area Under the Receiver Operating Characteristic curve; CPTAC: Clinical Proteomic Tumor Analysis Consortium; DGI: Deep Graph Infomax; GAT: graph attention network; GCN: graph convolutional network; GFM: gated fusion mechanism; GNN: graph neural network; IG: integrated gradients; MLP: multi-layer perceptron; MSE: mean squared error; PPI: protein–protein interaction; RF: Random Forest; SAM: self-attention mechanism; SVM: support vector machine; TCGA: The Cancer Genome Atlas.

## Supplementary Material

giag097_Supplemental_File

giag097_Authors_Response_To_Reviewer_Comments

giag097_GIGA-D-26-00172_original_submission

giag097_GIGA-D-26-00172_revision_1

giag097_Reviewer_1_Report_Original_Submission Reviewer 1 -- 6/16/2026

giag097_Reviewer_1_Report_Revision_1 Reviewer 1 -- 9/2/2026

giag097_Reviewer_2_Report_Original_Submission Reviewer 2 -- 6/21/2026

giag097_Reviewer_2_Report_Revision_1 Reviewer 2 -- 9/16/2026

giag097_Reviewer_3_Report_Original_Submission Reviewer 3 -- 7/1/2026

giag097_Reviewer_3_Report_Revision_1 Reviewer 3 -- 9/17/2026

## Data Availability

All datasets used in this study are publicly available from their respective original sources, including CLCLSA, HTML, MOGONET, SynOmics, and Li’s dataset [21, 22, 27, 30, 31]. The CPTAC datasets were obtained from the LinkOmicsKB database [64]. Detailed information on the data sources is provided in Supplementary Table S1.
