# Supplementary material for: GONet: a molecular interaction-guided graph learning framework for multi-omics cancer classification
Source: Gigascience. 2026 Oct 22;15:giag097. doi: 10.1093/gigascience/giag097 (PMC13741247; doi:10.1093/gigascience/giag097)
Supplement: Supplementary file 1 [file giag097_authors_response_to_reviewer_comments.pdf]

Author's Response To Reviewer Comments

Response to Reviewer #1:
1.The manuscript reports extensive benchmarking, but the reader may need clearer information on splits, cross-validation design, class balance, etc.
Response: I sincerely thank the reviewer for this valuable suggestion, and fully agree that the original manuscript lacked sufficient details regarding the data splitting, cross-validation strategy, and class balance. In the revised manuscript, I have expanded and clarified the experimental description in "Data splitting and model training" section (Lines 561-573) and provided Supplementary Fig. S3 to illustrate the overall workflow.
In the section, the revised statements regarding the data splitting and cross validation procedures are as follows "Each dataset was partitioned into a training set and an independent test set at an 8:2 ratio using stratified random splitting. For GONet, hyperparameters were optimized strictly in the 80% training set using 5-fold cross-validation. Based on the optimal hyperparameters determined via cross-validation, the final model was retrained on the entire 80% training set and subsequently evaluated on the held-out 20% independent test set. To assess the stability of GONet, the entire procedure was repeated five times using different random seeds, and the final performance metrics were reported as the average across these five iterations. All comparison methods, regardless of GNN or non-GNN, were evaluated using the same train-test splits to ensure a fair and consistent benchmarking comparison (Supplementary Fig. S3)".
For the question of class imbalance, we have summarized the sample sizes, and class distributions for each clinical label in Supplementary Table S1, indicating those datasets with class imbalance. In early version, we did consider impact of class imbalance, the Focal Loss[1] with α and γ parameters was taken to solve such problem. As described in the revised manuscript (Lines 537 to 538), "where p_y represents the predicted probability for true label y, and α and γ are hyperparameters controlling class weighting and focusing strength, respectively".

Supplementary Figure S3. Illustration of GONet hyperparameters and training strategy.
2.All preprocessing steps were performed inside each training fold?
Response: I completely agree with you that performing preprocessing steps within the training set is essential in deep learning. As described above and illustrated in Supplementary Figure S3, in each of the five splits, the original dataset was randomly divided into an 80% training set and a 20% independent test set. Subsequently, the learned parameters and selected features of preprocessing steps were determined using only the divided training sets and were applied to the corresponding independent test sets. The independent test set was not accessed during the preprocessing steps, thereby ensuring an unbiased evaluation of model performance.
3.The CPTAC pre-processing description states that features with more than 20% missing values were removed and remaining values were imputed with k-nearest neighbors, but the fold-wise handling of these steps is not sufficiently clear.
Response: Yes, I agree with your argument and have made the corresponding changes in the revision. All preprocessing procedures were independently performed in the five train-test splits, including missing value filtering, imputation, normalization, and feature selection, which were based on the preprocessing strategies of omics data previously reported[2][3]. Specifically, for the CPTAC datasets, the features with more than 20% missing values were removed, and the remaining missing values were imputed using KNN only based on the training samples. Besides, min-max normalization and ANOVA-based feature selection were also performed only using the training set. The resulting preprocessing parameters and selected features were then applied to the corresponding held-out 20% independent test set.
For example, the features selected by ANOVA using the training set were directly adopted in the corresponding test set. The same preprocessing strategy was consistently conducted to all CPTAC omics datasets. I have revised the relevant statements in "Data preprocessing" in Methods section (Lines 411-416) as "data preprocessing procedure followed a step-wise strategy in each random train-test splits, removal of features with more than 20% missing values, imputation of remaining missing values using k-nearest neighbors (KNN), min-max normalization, and ANOVA-based feature selection was performed on the training set. And then processing procedure and selected features were applied to the corresponding independent test sets".
To further clarify the preprocessing procedure, I have provided Supplementary Fig. S4 below to illustrate

this workflow.

Supplementary Figure S4. Illustration of data preprocessing.
4.The DGI module appears to be the main contributor, while the autoencoder and gated fusion provide smaller gains in the reported module tests; the manuscript would be stronger if it included an ablation of each component.
Response: Definitely, a comprehensive ablation analysis of each component would strengthen the argument for the contribution of each module in GONet. Following your suggestion, we evaluated GONet performance under different ablated conditions using the same CPTAC datasets with paired mRNA, miRNA, and proteomic profiles for the classification of cancer stages. Specifically, the ablation analysis of GONet was implemented under the following conditions: removal of AE for global sample representation module (noAE), replacement of DGI with GCN or GAT for local molecular representation module, removal of the self-attention mechanism for omics representation module (noOmics), and replacement of the gated fusion mechanism with direct feature concatenation for fusion module (cat). The results derived from such ablation analysis demonstrate that each component contributes to the overall performance of GONet.
As expected, removal of the AE led to decline in classification performance across the seven CPTAC cancer cohorts, replacement of DGI with GCN or GAT resulted in lower predictive performance, deletion of the omics representation module caused a moderate reduction in classification performance, and substitution of the gated fusion module made an average AUROC decrease, respectively (Supplementary Fig. S1 and Supplementary Tables S3-S4). These results indicate that although the modification of DGI provides the largest contribution, the other components capture complementary contributions to the overall performance of GONet.
In the revised manuscript, I have mentioned the description of the ablation analysis of GONet in “Results” section as follows: “To further evaluate the contribution of each architectural component to entire GONet, ablation analyses were adopted on the CPTAC datasets, like removal of AE, replacement of DGI, removal of omics representation and replacement of gated fusion. The ablation results endorsed the conclusions drawn from Figure 2A-D, with DGI playing a role of major contributor. Detailed ablation results are provided in Supplementary Fig. S1A-D and Supplementary Table S3-S4” (Lines 209-215).

Supplementary Figure S1. Module ablation analyses of GONet for cancer stage classification. (A) Ablation of the global sample representation module, (B) local molecular representation module, (C) omics representation module, and (D) feature fusion module.
Supplementary Table4: Quantitative summary of module-wise ablation analyses of GONet across seven CPTAC cancer cohorts for cancer stage classification.

Model	Mean AUROC	Relative drop	Interpretation
Full GONet	0.6777	0	Whole GONet
noAE	0.6130	10.55%	remove global representations
GCN	0.5581	21.43%	replace DGI with GCN
GAT	0.5899	14.87%	replace DGI with GAT
noOmics	0.6367	6.43%	remove omics representations
cat	0.6155	10.09%	replace gated-fusion with concatenation

5.The authors should discuss whether their approach recover cancer-relevant biology.
Response: I agree that biological relevance to the GONet prediction is essential to further strengthen the study significance beyond predictive performance. During manuscript preparation, we initially performed exploratory interpretive analyses of GONet predictions using the original cohort. As this cohort was primarily established for challenging TNM classification and comprised a relatively modest number of samples with diverse biological characteristics, we extended the analysis to a larger proteogenomic dataset with well-defined molecular subtypes, providing a more informative context for interpreting the biological relevance of GONet predictions. During the revision, we thus selected another independent Fu-iCCA cohort with a larger number of subtype cases of intrahepatic cholangiocarcinoma (iCCA), paired mRNA and proteomic data, and functional study to the disease[4]. Upon the Fu-iCCA dataset, GONet achieved a subtype classification performance with an AUROC of 0.98 (Supplementary Fig. S2A). Integrated Gradients (IG) was applied to quantify the contribution of individual molecular features to subtype prediction, yielding higher explanation fidelity than the random baseline (0.211 vs. 0.027) and a low attribution error (0.0278). Supplementary Table S7 summarizes the top 100 features with the highest IG attribution scores identified from the global and local modules. The top-ranked features derived from either the global or local modules across the mRNA and protein modalities indicate that GONet captures complementary biological signals

across multiple molecular layers rather than relying on a single omics modality (Supplementary Fig. S2B). Furthermore, functional enrichment analysis of these key transcriptomic and proteomic features reveals significant enrichment of biological processes and signaling pathways, which are consistent with previously reported biological characteristics of Fu-iCCA study (Supplementary Fig. S2C). Moreover, these key features are closely associated with subtype-differentially expressed genes or proteins (Supplementary Fig. S2D). Therefore, the interpretation analysis reveals the feasibility of biological relevance mining towards the GONet prediction.
I have given additional statements regarding biological interpretation towards the disease classification based on GONet in "Discussion" section of revised manuscript (Lines 385-391) as "Beyond predictive performance, exploring the relationships between GONet-based disease classification and molecular features may provide additional insights into the biological relevance of the predictions. As pointed out by Sundararajan et al, Integrated Gradients (IG) can be used to quantify the contribution of individual input features to model predictions and facilitate the identification of biologically relevant signals[5]. Further development of such interpretability analyses may provide additional biological insights into GONet-based disease classification".

Supplementary Figure S2. Interpretability analysis of GONet predictions using Integrated Gradients (IG) in an independent Fu-iCCA cohort. (A) Receiver operating characteristic curve demonstrating the subtype classification performance of GONet in the independent Fu-iCCA cohort. GONet achieved robust classification performance with an AUROC of 0.98. (B-C) Distribution of Integrated Gradients (IG) scores for the top 100 features identified from the global and local modules, stratified by molecular modality (transcriptomic and proteomic features). The results demonstrate that highly ranked predictive features were derived from both mRNA and protein modalities. (D) Sankey diagram illustrating the functional associations of the top-ranked mRNA and protein features identified by GONet. The diagram depicts the relationships among molecular features, enriched biological functions, and corresponding Fu-iCCA molecular subtypes, highlighting the biological processes and signaling pathways associated with the identified predictive features. (E) Box plots showing the subtype-specific distribution patterns of the prioritized molecular features. The consistent differential expression patterns across Fu-iCCA subtypes further support the biological relevance of the features identified by GONet.
6.The CPTAC performance remains moderate, and the authors already acknowledge dataset size and omics quality as limiting factors; phrases implying broad generalizability should be softened.
Response: I have accepted your advice to soften the tone regarding GONet performance. I have carefully reviewed and revised statements throughout the manuscript that may overstate the broader generalizability of GONet. Specifically, we have made the following changes:
(1) In the "Introduction" (Lines 131-132), the original statement: "Results from benchmark test and case studies demonstrated that GONet substantially improves predictive performance across diverse cancer datasets" has been corrected to: "Results from benchmark tests and case studies suggest that GONet can improve predictive performance across several cancer datasets".
(2) In "Results" Section 1 (Lines 219-227), the original statement: "As summarized in Fig2E, GONet consistently outperforms all competitors across datasets. On Li's dataset, it achieves an AUROC of 0.9663, representing a 6.39% improvement over Li's method (0.9082). Similar improvements are observed on MOGONET (0.9824 vs. 0.9520) and SynOmics (0.9849 vs. 0.9431), demonstrating strong cross-dataset generalization" has been revised to: "As summarized in Fig2E, GONet was evaluated for cancer classification across the datasets used for the development of these GNN-based methods. On Li's dataset, GONet achieved an AUROC of 0.9663, representing a 6.39% improvement over Li's method (0.9082). On the MOGONET datasets, GONet yielded an AUROC of 0.9824, higher than those of MOGONET (0.9520), MGDMCL (0.9329), and MOGANet (0.9386), respectively. GONet also obtained an AUROC of 0.9849 on the SynOmics dataset, compared with 0.9431 for SynOmics. Together, these results demonstrate the improved prediction of cancer classification performance by GONet".
(3) In "Results" Section 4 (Lines 330-331), the original statement: "the null hypothesis of equal performance is rejected ($p < 0.001$), confirming the statistically significant superiority of GONet across diverse evaluation scenarios" has been changed to: "the null hypothesis of equal performance was rejected ($p < 0.001$), indicating that GONet achieves statistically difference in the evaluation scenarios".
(4) In the "Discussion" (Lines 401-402), the original statement: "GONet has established a robust and generalizable multi-omics integration framework that provides a methodological foundation for disease prediction" has been restated as: "GONet has established a multi-omics integration framework that potentially provides a methodological foundation for disease classification".

Reference：
[1] Lin, T.-Y., Goyal, P., Girshick, R., He, K. & Dollár, P. Focal Loss for Dense Object Detection. in 2980–2988 (2017).
[2] Wang, T. et al. MOGONET integrates multi-omics data using graph convolutional networks allowing patient classification and biomarker identification. Nat. Commun. 12, 3445 (2021).
[3] Ji, Y., Dutta, P. & Davuluri, R. Deep multi-omics integration by learning correlation-maximizing representation identifies prognostically stratified cancer subtypes. Bioinforma. Adv. 3, vbad075 (2023).
[4] Dong, L. et al. Proteogenomic characterization identifies clinically relevant subgroups of intrahepatic cholangiocarcinoma. Cancer Cell 40, 70–87 (2022).
[5] Sundararajan, M., Taly, A., & Yan, Q. (2017). Axiomatic Attribution for Deep Networks. Proceedings of the 34th International Conference on Machine Learning (ICML), 3319–3328.

Response Reviewer #2:
1.Please explicitly discuss the distinction between the proposed model and existing methods in the literature review paragraphs of the Introduction section.
Response: I sincerely thank you very much for the valuable comments about clearer explanation of the innovation and necessity of our approach compared with previous studies. In response to this comment, we have revised the Introduction section of the new manuscript (Lines 67-121), and the revised text is provided below:
Benefiting from strong capabilities in nonlinear modeling and representation learning, deep learning has become a major methodology for multi-omics integration in cancer analysis16–19. Early deep learning approaches that primarily rely on non-GNN architectures, such as autoencoders (AEs), multilayer perceptrons (MLPs) and attention-based models, transform different omics profiles into unified representations and alleviate problems of high dimensionality and noise. For example, MOCAT is an AE-based framework that integrates mRNA, miRNA, and DNA methylation data across multiple diseases, including Alzheimer’s disease and several cancer types, achieving improved classification performance in disease diagnosis, subtype and stage prediction20. Attention-based models have been introduced to enhance multi-omics representation learning by dynamically weighting contributions from different omics features. Methods such as HTML and CLCLSA combine attention mechanisms with contrastive learning strategies and achieve well-improved performance in multi-cancer classification tasks21,22. GNNs, another important class of deep learning models, are designed to capture relational dependencies in graph-structured data through message passing between nodes23–25. Early GNN-based multi-omics approaches are primarily constructed using sample-level similarity graphs for classification tasks, in which nodes represent individual samples and edges encode pairwise relationships derived from omics features. For instance, MOGONET employs graph convolutional networks (GCNs)26 on sample similarity graphs constructed from mRNA, miRNA, and DNA methylation data for prediction of cancer subtypes/stages27. MOGCN constructs sample similarity graphs from CNV, mRNA, and RPPA data in TCGA breast cancer and is applied to subtype classification28. However, those approaches, either traditional non-GNN or sample-similarity-based GNN models, focus on learning global representations at the sample level from the gene expression matrices to integrate omics data. As a result, they cannot utilize the interaction information and are limited in capturing complex nonlinear dependencies across omics features, thereby leaving substantial room for improvement in representation learning for disease and cancer subtype prediction.
Cancer is not solely driven by single-gene aberrations but is regulated by complex molecular interaction networks. If interaction graphs are integrated into GNN-based multi-omics integration frameworks, with nodes representing gene features and edges characterizing the feature interactions, such a design is expected to enable GNNs to learn biologically local representations from molecular interaction topology and improve disease prediction10,16,29. Recently, SynOmics has extended GCN-based26 architectures to intra- and inter-omic interaction graphs for cancer prognosis prediction30, while Li et al. proposed a hybrid model to improve prediction performance for breast cancer subtype classification32, in which MLPs encode global omics profiles and GCN or GAT31 models molecular interaction networks. Nevertheless, there remains large room for improvement in current GNN-based models. First, feature extraction is insufficient across multiple levels. MLP-based encoders tend to compress high-dimensional omics data toward task-specific representations, potentially losing informative variations. Standard message-passing in GNNs is largely confined to local neighborhoods, and may therefore have limited ability to capture broader dependencies. In addition, most current methods assume homogeneous node representations, whereas they ignore intrinsic heterogeneity across omics types such as mRNA and proteins. Therefore, a novel feature extractor is required to be capable of representing both omics characteristics and interaction

patterns. Second, representation fusion remains insufficiently coordinated. Since simple concatenation may lead to unstable hierarchical representations and weak discrimination power in downstream tasks, a new fusion mechanism that can resolve conflicts and inconsistencies among multiple representations is highly desirable. Third, with the growing proteomic evidence for cancer classification, a promising avenue is to introduce proteomics and the corresponding interaction networks into GNN-based models to enhance discriminative capacity33–36.

2.Most of the baseline methods compared in this paper were published very early. The authors are suggested to compare with the methods in recent years.

Response: I agree that comparisons with recently developed multi-omics integration methods would provide a more comprehensive evaluation of GONet. To address this concern, we conducted an extensive survey of recent publications on multi-omics integration approaches from 2025 to 2026, and found that GNN-based approaches have become increasingly prevalent in this field; however, these approaches are fundamentally different from our model, such as molecular interactions and proteomics. Therefore, we have expanded the comparison study of GONet by including two additional recent GNN-based methods, MGDMCL (2025), and MOGANet (2026), as well as a new non-GNN-based method, MOAEAM (2026). Following the dataset-based comparison strategy used in the previous version of our manuscript, GONet was evaluated on the datasets used for the development of these methods. Briefly, the AUROC values obtained by MGDMCL, MOGANet, and MoAEAM are 0.9329, 0.9386, and 0.9305, respectively, whereas GONet achieves an AUROC of 0.9824 on the same dataset, which is higher than those obtained by the three new methods. In addition, the CPTAC datasets were also used for the performance evaluation in these methods, and the comparisons indicate that GONet also achieves competitive performance on the CPTAC datasets as well. Overall, these additional comparisons further support the effectiveness of GONet when evaluated against recently developed multi-omics integration methods across both their benchmark datasets and the CPTAC datasets used for evaluation.

In the revised manuscription, I have made the corresponding changes in the “Results” Section 2 as follows “(Lines 186-189) GNN-based methods have been applied to cancer type and stage prediction using multi-omics data in recent five years, including approaches with or without explicit modeling of interaction information, such as MOGONET27, MOGCN28, SynOmics30, and Li’s method31, MGDMCL40, and MOGANet41”, “(Lines 219-227) As summarized in Fig2E, GONet was evaluated for cancer classification across the datasets used for the development of these GNN-based methods. On Li’s dataset, GONet achieved an AUROC of 0.9663, representing a 6.39% improvement over Li’s method (0.9082). On the MOGONET datasets, GONet yielded an AUROC of 0.9824, higher than those of MOGONET (0.9520), MGDMCL (0.9329), and MOGANet (0.9386), respectively. GONet also obtained an AUROC of 0.9849 on the SynOmics dataset, compared with 0.9431 for SynOmics. Together, these results demonstrate the improved prediction of cancer classification performance by GONet”, and “(Lines 232-236) GONet consistently yielded higher prediction performance than other GNN-based methods for cancer stage classification across the CPTAC datasets, with an average AUROC of 0.6420, corresponding to 16.41% relative improvement over Li’s method, 39.78% over MOGONET, 29.15% over MOGCN, 16.20% over SynOmics, 21.54% over MOGANet (0.5282), and 31.75% over MGDMCL (0.4873)”. Meanwhile, Figure 2E has been updated to include the newly added comparison results as indicated below.

Also, I have made the restatements regarding non-GNN-based methods in Results Section 4 in the revision as follows “(lines290-293) A wide range of non-GNN-based multi-omics integration methods have been applied to cancer type and stage classification, including several conventional methods, such as RF43, SVM44, LR45, AE, MLP, and recently published approaches, such as HTML22, CLCLSA21, and MOAEAM48”, “(lines 308-313) the different approaches exhibited variable prediction performance on the remaining 8 datasets (Fig 4C). GONet consistently showed higher prediction performance than the comparison methods, with an average AUROC of 0.9495 versus 0.8687 for the non-GNN methods. Notably, the GNN-based comparison methods showed lower prediction performance on the same datasets, with an average AUROC of 0.7465”, “(lines 316-318) All the non-GNN methods were applied to the CPTAC datasets for assessment of cancer stage classification. As shown in Fig. 4D, these methods achieve AUROC values ranging from 0.5080 to 0.6226”, and “(lines 324-327) As shown in Fig 4E, non-GNN-based approaches exhibited lower predictive performance, with average AUROC values spanning 0.4780 to 0.6012 ($p<0.05$). Moreover, proteomics integration led to inconsistent gains across methods and across the 7 cancer types (Fig 4F)”. Also, the previous Figure 4 has also been revised in the revision as below.

3.The manuscript did not provide details on hyperparameter selections. Please include a sensitivity analysis

versus key hyperparameters to ensure a fair comparison.
Response: I am sorry that we did not provide sufficient details on the hyperparameter selection in the original version, although relevant information was provided in the Supplementary Materials. Following your advice, we have performed a sensitivity analysis of the key hyperparameters during the revision of manuscript.
In the revised manuscript, in "Hyperparameters Optimization" of "Methods" section, I have given the detailed description of hyperparameter selection as follows: "The hyperparameters of GONet were optimized on the training set using Optuna66, a framework based on Bayesian optimization principles. Specifically, the optimization was conducted within a predefined search space through many independent trials. In each trial, Optuna proposed a candidate hyperparameter configuration based on the results of previous trials, which was then evaluated using 5-fold cross-validation. The mean AUROC across the five folds was used as the performance metric to guide selection of the next candidate configuration of hyperparameters. After completing the optimization process, the hyperparameters configuration yielding the highest mean AUROC was selected as the final configuration for GONet (see Table S5-S6)" (Lines 575-583).
To implement the sensitivity analysis of the key hyperparameters, we selected three key hyperparameter, including learning rate, AE depth, and GNN depth, and tested the GONet performance by varying one hyperparameter while remaining the other hyperparameters no change across CPTAC datasets.
For learning rate, we evaluated the GONet performance under five learning rate settings, 1e-5, 3e-5, 1e-4, 3e-4, and 1e-3. As shown in Figure 1A below, GONet showed relatively stable performance across learning rates ranging from 3e-5 to 3e-4 in different datasets, indicating that GONet is robust within a reasonable range of learning rates. For AE depth, we assessed the GONet performance under different AE depths ranging from 2 to 5 layers. As presented in Figure 1B below, the GONet performance varied with AE depth settings in different datasets, with shallow networks (2-3 layers) performing better for LUAD and HNSCC, whereas deeper layers provided better performance in some other cohorts. These results suggest that optimal AE depth of GONet should be selected on different multi-omics datasets. For GNN depth, we evaluated GNN depths ranging from 2 to 5 layers. As shown in Figure 1C below, the optimal performance of GONet was mainly achieved with 2 or 3 layers, suggesting that GNN with deeper layers is not always beneficial for the GONet performance.
Taken together, the sensitivity analysis indicates that GONet performance varies with different parameter settings across datasets, while no single hyperparameter setting consistently dominates across all cohorts. These results further suggest dataset-specific hyperparameter optimization rather than fixed hyperparameters. Detailed hyperparameter search spaces and optimal values from Optuna are provided in Supplementary Table S5-S6.

Figure 1. Sensitivity analysis of GONet to key hyperparameters across seven cancer cohorts. (A) Learning rate, (B) autoencoder depth, and (C) GNN depth. In each analysis, one hyperparameter was varied while all other hyperparameters were fixed at the corresponding optimized configuration. Each point represents the mean AUROC obtained from five-fold cross-validation.
4.The tick marks and labels on the axes should be enlarged to improve readability for the readers.
Response: Yes, I have thoroughly revised Figures 2-4 by enlarging the font sizes of the axis labels, tick marks, and legends to improve readability. These figures have been updated accordingly in the revised manuscript.
5.What are the configurations of the simulation computer used for running these models? This hardware information can be added to the manuscript.
Response: Following your suggestion, I have added the configuration information of hardware and software to "Data splitting and model training" section of the revised manuscript (Lines 571-573) as follows: "All experiments were performed on a Linux system equipped with an NVIDIA A100 GPU (80 GB VRAM). The GONet were implemented in Python 3.8, using PyTorch 2.0 with CUDA 11.8, and PyTorch Geometric (PyG) 2.4.0".

Reviewer #3:
1.First, although Deep Graph Infomax (DGI) alleviates the over-smoothing issue of GCN/GAT through mutual information maximization, DGI is still essentially a local contrastive learning method with limited capability to capture long-range dependencies. The hierarchical regulatory relationships within omics (e.g., transcription factor-mRNA-protein cascade regulation) were not considered.
Response: I agree that DGI is fundamentally a local contrastive learning framework and has limited

capacity to capture long-range dependencies. On the other hand, DGI can maximize mutual information between local node representations and an entire graph representation, encouraging the aggregation of nodes beyond neighborhoods to a certain extent[1], yet the encoder itself remains local. In our study, hierarchical regulatory relationships (e.g., transcription-factor-miRNA-target cascades) are directly encoded in the different multi-omics graph of GONet, which comprises mRNA, miRNA and protein nodes connected by both intra-omics and inter-omics edges, while DGI learns representations on the heterogeneous graph structure. I would address the reviewer's concern at two complementary angles, whether cross-omics information contributes to predictive performance, and whether biologically hierarchical interactions contribute to the model's predictions.

First, the contribution of cross-omics information is supported by both graph statistics and predictive performance. As shown in Fig. 1A, the integrated tri-omics networks exhibit higher average node degrees (approximately 112-144 across the CPTAC cohorts) than the corresponding single-omic networks, providing more opportunities for multi-hierarchical information flow across molecular layers. Moreover, under the same DGI framework, the integrated mRNA-miRNA-protein model achieved improved predictive performance compared with individual single-omic models for stage classification across different cancer cohorts on CPTAC datasets (Results section 3). Particularly, the integrated model achieved an average AUROC of 0.68, compared with 0.64 (protein-only), 0.57 (miRNA-only) and 0.60 (mRNA-only). These results support the utility of cross-omics information for prediction performance.

Second, regarding whether hierarchical relationships contribute to the predictions, we applied Integrated Gradients (IG) to quantify the importance of individual edges[2]. Using CPTAC-LUAD as a representative cohort, we calculated an IG attribution score for each edge and retained the top 25% of edges ranked by their absolute attribution scores (47,714 edges). As illustrated in Fig. 1B, these highly attributed edges contain both within-omics interactions and cross-omics relationships. Importantly, some biologically supported hierarchical regulatory relationships were also identified among high attribution edges, including not only direct molecular interactions but also hierarchical cross-omic regulatory dependencies. For example, as illustrated in Fig. 1C, CDH1-EGFR represents a direct protein-level interaction[3], whereas SRF → miR-199a-5p → CDH1 forms a TF-miRNA-target cascade including transcriptional and post-transcriptional regulation[4]. Similarly, miR-9/AGO2-mediated regulation of ACOT7 represents a post-transcriptional regulatory relationship[5], while SRF-ACOT7 is supported by TF-binding evidence[6]. Additional high-attribution interactions previously reported in the literature (EGFR-miR-200a[7], miR-21-CDH1[8], SRF-MCL1[9]) further illustrate that specific hierarchical cross-omics interactions contribute to the model's predictions.

Figure 1. Cross-omics network connectivity and biologically relevant interactions captured by GONet. (A) Degree distribution of integrated tri-omics and single-omic networks across CPTAC cancer cohorts. (B) The network graph of highly attributed molecular relationships identified by Integrated Gradients (IG). (C) Representative physical interactions and hierarchical regulatory relationships among highly attributed interactions.

Table 1: Literature evidence supporting highly attributed molecular interactions identified by Integrated Gradients.

No. Relationships PMID Cancer/model Main finding
1 EGFR - CDH1 35074920 epithelial cells heteroreceptor complex
2 EGFR - miR-21 19597153 Lung cancer EGFR positively regulates miR-21
3 EGFR - miR-200a 29552158 NSCLC EGFR/ERK/miR-200a signaling
4 miR-21 - CDH1 30568467 NSCLC miR-21 increases SMAD7, reduces E-cadherin
5 miR-9/AGO2 - ACOT7 28472401 Human cells miR-9/WIG1/AGO2-dependent ACOT7 silencing
6 EGFR - AGO2 23636329 Cancer cell EGFR phosphorylates AGO2-Y393
7 SRF - miR-199a- CDH1 25080937 Cross-cancer evidence SRF transactivates miR-199a, miR-199a-5p targets E-cadherin
8 SRF - MCL1 9880563 Hematopoietic SRF/Elk-1 transcriptionally regulates MCL1
9 SRF – ACOT7 27426982 TFBS TFBS-supported
10 ACOT7 - phenotype 36139682 LUAD/PC9 ACOT7 knockdown suppresses proliferation/migration
11 MCL1 - phenotype 31200834 LUAD MCL1 supports survival
12 CDH1- phenotype 32015681 LUAD Promotes self-renewal and stemness in lung cancer stem-like cells
13 miR-21 - phenotype 29663730/34858053 LUAD miR-21 promotes EMT/invasion; high miR-21 predicts poor prognosis
14 miR-199a - phenotype 33868472/35844793 NSCLC miR-199a suppresses tumor phenotypes

2.Second, proteomics was evaluated only in seven cancer types from CPTAC with relatively small sample sizes, leading to moderate absolute AUROC values. Although an attention mechanism was employed, no biological interpretation was provided for the learned attention weights (e.g., which omics are more important in specific cancer types, which molecular interactions are most critical for classification).
Response: During the revision, I have followed your suggestion and performed an attention-based interpretability analysis to answer which omics are more important in specific cancer types, and which molecular interactions are most critical for classification.
To quantify the attention-based importance of each omics layer during information aggregation, an omics-level attention contribution score C_j based on the learned attention weight matrix A was calculated as follows:

$$C_j=\frac{1}{M}\sum_{i=1}^{M}A_{ij}$$

where A_{ij} denotes the attention weight directed from omics j to omics i, and M represents the total number of omics modalities (mRNA, miRNA, and protein). Thus, C_j represents the average attention weight assigned to omics j during cross-omics information aggregation[10,11] (Wang et al., 2024; Cai et al., 2024). Based on the C_j values across different cancer datasets, proteomics receives higher attention scores in BRCA and HNSCC, miRNA in LSCC, CCRCC, LUAD, and COAD, and mRNA in PDAC, respectively, suggesting that the relative attention contribution to different omics in GONet varies across cancer types (Figure 2A). This observation is consistent with previous studies reporting that the predictive contribution of individual omics modalities varies considerably across cancer types[12].
To further explore the cross-omics interactions learned by GONet, the attention weights A_{ij} are used to quantify the relative attention assigned to interactions between omics i and omics j. As shown in Figure 2B-H, the distribution of attention weights also varies across cancer types. The interactions involving miRNA are prominent in LUAD, LSCC, COAD and CCRCC, mRNA-related interactions in PDAC, and protein-related interactions in BRCA and HNSCC. There is no single interaction with consistently high attention contribution scores across these datasets. However, the attention contribution of individual omics is consistent with that of their corresponding interactions. For instance, C_j is the highest values for proteomics in BRCA and HNSCC, and the A_{ij} values for proteomics-related interactions are also relatively high in these two datasets.

Figure2. Multi-omics importance weights and critical molecular interaction networks across cancer types for classification. (A). heatmap of omics-specific importance weights (mRNA, protein, miRNA) for seven cancer types (BRCA, LSCC, CCRCC, PDAC, LUAD, HNSCC, COAD), highlighting which omics contribute most to classification in each cancer. (B-H) multi-omics interaction networks showing the critical molecular interactions (edge weights) among mRNA, miRNA, and protein for each cancer type (PDAC, LUAD, LSCC, HNSCC, COAD, CCRCC, BRCA).

3.Third, the method heavily relies on curated interaction networks from public databases (e.g., BioGRID, STRING). Experiments were mainly conducted on common cancer types (e.g., BRCA, LUAD), and the applicability to rare cancers or small-sample scenarios remains unclear. No direct association was established with clinical indicators (e.g., survival prognosis, treatment response).
Response: I fully acknowledge that GONet still has several limitations, especially regarding its applicability to rare cancers, small-sample scenarios, and in clinical interpretation.
Regarding the issues of the rare cancers and small-sample scenarios, the applicability and performance of GONet in these settings remain to be determined. To develop and evaluate GONet, we do need the basic prerequisites, such as sufficient multi-omics data for integration, enough large sample size for model training, and established methods for multi-omics data integration for cancer classification. At present, publicly available studies and datasets for rare cancers are often difficult to match these prerequisites. Therefore, our study has focused primarily on common cancer types for which sufficient cancer studies and datasets are available to meet these prerequisites. I fully agree that application of GONet to rare cancer and small-sample scenarios is an important direction for future model development.
Regarding the clinical relevance, the solid cancer datasets used in this study are derived from TCGA and CPTAC with well-defined clinically annotations, which provided the foundation for developing and evaluating GONet for cancer classification. Since GONet can effectively recognize cancer types and stages, which are clinically relevant characteristics, its predictions are partially related to clinical indicators. However, I have to acknowledge that the current version of GONet does not provide the clinical interpretation of the prediction results. This limitation may provide an important direction for the future development of GONet, particularly through the introduction of integrated gradients (IG) to identify important predictive features

and explore their associations with clinical outcomes, such as survival, treatment response, and drug sensitivity.

Reference:
[1] Veličković P, Fedus W, Hamilton W L, et al. Deep Graph Infomax[A]. arXiv, 2018.
[2] Sundararajan M, Taly A, Yan Q. Axiomatic Attribution for Deep Networks[C]//International conference on machine learning. 3319-3328.
[3] Sullivan B, Light T, Vu V, et al. Mechanical disruption of E-cadherin complexes with epidermal growth factor receptor actuates growth factor-dependent signaling[J]. Proceedings of the National Academy of Sciences of the United States of America, 2022, 119(4): e2100679119.
[4] Zhao X, He L, Li T, et al. SRF expedites metastasis and modulates the epithelial to mesenchymal transition by regulating miR-199a-5p expression in human gastric cancer[J]. Cell Death and Differentiation, 2014, 21(12): 1900-1913.
[5] Lee H C, Jung S H, Hwang H J, et al. WIG1 is crucial for AGO2-mediated ACOT7 mRNA silencing via miRNA-dependent and -independent mechanisms[J]. Nucleic Acids Research, 2017, 45(11): 6894-6910.
[6] Plaisier C L, O'Brien S, Bernard B, et al. Causal Mechanistic Regulatory Network for Glioblastoma Deciphered Using Systems Genetics Network Analysis[J]. Cell Systems, 2016, 3(2): 172-186.
[7] Zhou P, Hu J, Wang X, et al. Epidermal growth factor receptor expression affects proliferation and apoptosis in non-small cell lung cancer cells via the extracellular signal-regulated kinase/microRNA 200a signaling pathway[J]. Oncology Letters, 2018, 15(4): 5201-5207.
[8] Li X, Wu X. MiR-21-5p promotes the progression of non-small-cell lung cancer by regulating the expression of SMAD7[J]. OncoTargets and Therapy, 2018, 11: 8445-8454.
[9] Townsend K J, Zhou P, Qian L, et al. Regulation of MCL1 through a serum response factor/Elk-1-mediated mechanism links expression of a viability-promoting member of the BCL2 family to the induction of hematopoietic cell differentiation[J]. The Journal of Biological Chemistry, 1999, 274(3): 1801-1813.
[10] Cai J, Wang R, Zhao D, et al. AuD-Former: A Hierarchical Transformer Network for Multimodal Audio-Based Disease Prediction[J]. arXiv preprint arXiv:2410.09289
[11] Wang Y, He J, Wang D, et al. Multimodal transformer with adaptive modality weighting for multimodal sentiment analysis[J]. Neurocomputing, 2024, 572: 127181.
[12] Su X, Hu P, Li D, et al. Interpretable identification of cancer genes across biological networks via transformer-powered graph representation learning[J]. Nature Biomedical Engineering, 2025, 9(3): 371-389.

---

em Editorial Manager®

Cookie Preference Center

We use cookies which are necessary to make our site work. We may also use additional cookies to analyze, improve and personalize our content and your digital experience. For more information, see our Cookie Policy.

You may choose not to allow some types of cookies. However, blocking some types may impact your experience of our site and the services we are able to offer. See the different category headings below to find out more or change your settings.

Allow all

Manage Consent Preferences

Strictly Necessary Cookies

Always active

These cookies are necessary for the website to function and cannot be switched off in our systems. They are usually only set in response to actions made by you which amount to a request for services, such as setting your privacy preferences, logging in or filling in forms. You can set your browser to block or alert you about these cookies, but some parts of the site will not then work.

Functional Cookies

These cookies enable the website to provide enhanced functionality and personalisation. They may be set by us or by third party providers whose services we have added to our pages. If you do not allow these cookies then some or all of these services may not function properly.

Performance Cookies

These cookies allow us to count visits and traffic sources so we can measure and improve the performance of our site. They help us to know which pages are the most and least popular and see how visitors move around the site.

Targeting Cookies

These cookies may be set through our site by our advertising partners. They may be used by those companies to build a profile of your interests and show you relevant adverts on other sites. If you do not allow these cookies, you will experience less targeted advertising.

Cookie List

Clear

○

Apply Cancel

Consent Leg.Interest

Confirm my choices

Power
