# Supplementary material for: GONet: a molecular interaction-guided graph learning framework for multi-omics cancer classification
Source: Gigascience. 2026 Oct 22;15:giag097. doi: 10.1093/gigascience/giag097 (PMC13741247; doi:10.1093/gigascience/giag097)
Supplement: Supplementary file 2 [file giag097_giga-d-26-00172_original_submission.pdf]

GigaScience

GONet: A Molecular Interaction-Guided Graph Learning Framework for Multi-Omics Cancer Classification

--Manuscript Draft--

**Manuscript Number:**	GIGA-D-26-00172
**Full Title:**	GONet: A Molecular Interaction-Guided Graph Learning Framework for Multi-Omics Cancer Classification
**Article Type:**	Research
**Funding Information:**	Key Technologies Research and Development Program (2023YFC3402900) — Dr. Guixue Hou
**Abstract:**	Integrating multi-omics profiles with molecular interaction networks enables biologically informed representation learning by combining molecular measurements with prior biological knowledge. Although graph neural networks (GNNs) are well suited for modeling molecular interaction networks, existing multi-omics methods often rely on shallow representation learning or incomplete fusion strategies, which fail to fully capture complementary information and complex dependencies across heterogeneous omics layers, resulting in suboptimal performance and limited generalizability in cancer classification tasks.<br>Here, we propose GONet, a hierarchical GNN framework that integrates omics expression profiles, including mRNA, miRNA, and protein data, with molecular interaction networks, where nodes represent omics features and edges represent molecular interactions. GONet learns global omics representations via an autoencoder, captures local structural dependencies using Deep Graph Infomax (DGI), and enhances omics-specific representations through an attention mechanism. A gated fusion module further enables adaptive integration of heterogeneous features, improving cancer subtype and stage classification.<br>GONet was systematically evaluated on 13 publicly available benchmark datasets used in prior studies, covering 10 cancer types, ensuring fair comparison across methods. Across mRNA and miRNA datasets, GONet achieved an average AUROC of 0.9262, outperforming representative GNN- and non-GNN-based methods by 13.08% and 5.48%, respectively. On an independent CPTAC cohort spanning seven cancer types, it improved performance by 25.61% and 16.03%, respectively. Incorporating proteomics further improved cancer stage classification by 5.56%, demonstrating complementary predictive value. Ablation studies confirmed the effectiveness of the DGI-based module. Overall, GONet provides a unified framework for biologically informed multi-omics graph representation learning and cancer classification.
**Corresponding Author:**	Siqi Liu, PhD<br>BGI-Shenzhen: BGI Group<br>Shenzhen, Guangdong CHINA
**Corresponding Author Secondary Information:**	
**Corresponding Author's Institution:**	BGI-Shenzhen: BGI Group
**Corresponding Author's Secondary Institution:**	
**First Author:**	Yiyang Chen
**First Author Secondary Information:**	
**Order of Authors:**	Yiyang Chen
	Yunning Lu
	Da Qi
	Yong Bai
	Guixue Hou

	Siqi Liu
**Order of Authors Secondary Information:**	
**Additional Information:**	
**Question**	**Response**
Are you submitting this manuscript to a special series or article collection?	No
**Experimental design and statistics**<br><br>Full details of the experimental design and statistical methods used should be given in the Methods section, as detailed in our Minimum Standards Reporting Checklist. Information essential to interpreting the data presented should be made available in the figure legends.<br><br>Have you included all the information requested in your manuscript?	Yes
**Resources**<br><br>A description of all resources used, including antibodies, cell lines, animals and software tools, with enough information to allow them to be uniquely identified, should be included in the Methods section. Authors are strongly encouraged to cite Research Resource Identifiers (RRIDs) for antibodies, model organisms and tools, where possible.<br><br>Have you included the information requested as detailed in our Minimum Standards Reporting Checklist?	Yes
**Availability of data and materials**<br><br>All datasets and code on which the conclusions of the paper rely must be either included in your submission or deposited in publicly available repositories (where available and ethically appropriate), referencing such data using a unique identifier in the references and in	Yes

the “Availability of Data and Materials” section of your manuscript.<br><br>Have you have met the above requirement as detailed in our Minimum Standards Reporting Checklist?	
GigaScience has policies and guidelines in place for the use of generative AI-writing tools such as ChatGPT. If you have used such writing tools to assist with writing the manuscript this must be declared and cited in the text. Authors should not list AI-writing tools and other AI-assisted technologies as an author or co-author and should acknowledge that they are fully responsible for text generated or refined by AI-writing tools.<p><br><br>A summary of use (particularly in the introduction or among methods) needs to be included at the end of the paper, and the outputs should also be included as a supplementary file hosted in GigaDB or other open repositories. Please read our guidelines for more information. <p><br><br>By submitting to GigaScience, you are aware of the journal's AI-writing tools policy, and if you have declared use of such tools below, you have acknowledged this where appropriate in your manuscript and have made a summary of use and outputs available. <p><br><b>AI-assisted writing tools have been used in the preparation of this manuscript?	No

manuscript
Click here to access/download;Manuscript;GONet-manuscript_updated.pdf

GONet: A Molecular Interaction-Guided Graph Learning Framework for Multi-Omics Cancer Classification

Yiyang Chen (陈毅洋)[1,2], Yunning Lu (陆运凝)[2,3], Da Qi (戚达)[2], Yong Bai (白勇)[4,*], Guixue Hou (侯桂雪)[2,*], Siqi Liu (刘斯奇)[1,2,*]

chenyiyang@genomics.cn; luyunning@bgi.cn; mr_daqi@hotmail.com; Yong.bai@hotmail.com; houguixue@bgi.com; siqiliu@genomics.cn

1 College of Life Sciences, University of Chinese Academy of Sciences, Beijing 100049, China
2 BGI-Shenzhen, Shenzhen, Guangdong 518083, China
3 School of Biology and Biological Engineering, South China University of Technology, Guangzhou, 510641, China
4 BGI Research, Shenzhen, 518083, China
*Corresponding authors: siqiliu@genomics.cn; houguixue@bgi.com; Yong.bai@hotmail.com.

Abstract

Integrating multi-omics profiles with molecular interaction networks enables biologically informed representation learning by combining molecular measurements with prior biological knowledge. Although graph neural networks (GNNs) are well suited for modeling molecular interaction networks, existing multi-omics methods often rely on shallow representation learning or incomplete fusion strategies, which fail to fully capture complementary information and complex dependencies across heterogeneous omics layers, resulting in suboptimal performance and limited generalizability in cancer classification tasks.

Here, we propose GONet, a hierarchical GNN framework that integrates omics expression profiles, including mRNA, miRNA, and protein data, with molecular

interaction networks, where nodes represent omics features and edges represent molecular interactions. GONet learns global omics representations via an autoencoder, captures local structural dependencies using Deep Graph Infomax (DGI), and enhances omics-specific representations through an attention mechanism. A gated fusion module further enables adaptive integration of heterogeneous features, improving cancer subtype and stage classification.

GONet was systematically evaluated on 13 publicly available benchmark datasets used in prior studies, covering 10 cancer types, ensuring fair comparison across methods. Across mRNA and miRNA datasets, GONet achieved an average AUROC of 0.9262, outperforming representative GNN- and non-GNN-based methods by 13.08% and 5.48%, respectively. On an independent CPTAC cohort spanning seven cancer types, it improved performance by 25.61% and 16.03%, respectively. Incorporating proteomics further improved cancer stage classification by 5.56%, demonstrating complementary predictive value. Ablation studies confirmed the effectiveness of the DGI-based module. Overall, GONet provides a unified framework for biologically informed multi-omics graph representation learning and cancer classification.

Keywords: multi-omics data integration, graph neural network, cancer classification, molecular interaction network

Introduction

Cancer remains one of the leading causes of morbidity and mortality worldwide[1], driven by highly complex and heterogeneous molecular mechanisms. Elucidating these mechanisms is essential for understanding cancer initiation and progression, as well as for improving clinical diagnosis, treatment, and prevention[2,3]. The advent of high-throughput omics technologies has enabled systematic characterization of cancer across multiple molecular layers[4,5]. Genomics reveals the landscape of genetic alterations underlying cancer development[4,6], while transcriptomics and proteomics provide gene expression patterns, including gene expression dynamics, post-translational modifications, and molecular interactions[4,7,8]. In essence, cancer initiation and development are cumulative results of genetic mutations[9]; therefore, the pronounced heterogeneity of cancers is likely associated with the interactions across networks involving genome, transcriptome, and proteome[4,10]. Although single-omics data provide rich experimental evidences and bioinformatic insights at the molecular level, from the perspective of the central dogma[11], interactions across different molecular levels during the transmission of genetic information also influence biological phenotypes[12,13]. Consequently, integrating multi-omics information is essential for a comprehensive characterization of cancer biology[5,14,15]. With continuous accumulation of large-scale resources, such as The Cancer Genome Atlas (TCGA)[3], and the Clinical Proteomic Cancer Analysis Consortium (CPTAC)[2], researchers have increasingly focus on multi-omics integration studies.

Benefiting from strong capabilities in nonlinear modeling and representation learning, deep learning has become a main methodology for multi-omics integration in cancer analysis[16–19]. Early deep learning approaches primarily relied on non-GNN architectures, such as autoencoders (AEs) or multilayer perceptrons (MLPs), which project different omics profiles into unified representations and alleviate problems of high dimensionality and noise. For example, MOCAT is an AE-based framework that integrates mRNA, miRNA, and DNA methylation data across multiple diseases, including Alzheimer’s disease and several cancer types, achieving improved classification performance in disease diagnosis, subtype identification, and stage prediction[20]. Attention-based models has recently been introduced to enhance multi-omics representation learning by dynamically weighting contributions from different omics features. Methods such as HTML and CLCLSA combine attention mechanisms with contrastive learning strategies, achieving improved performance in multi-cancer classification tasks[21,22]. GNNs, another important class of deep learning models, are designed to capture relational dependencies in graph-structured data through message passing between nodes[23–25]. Early GNN-based multi-omics approaches are primarily construct sample-level similarity graphs for classification tasks, where nodes represent individual samples and edges encode pairwise relationships derived from omics features, e.g., feature correlations. For instance, MOGONET employs graph convolutional networks (GCNs)[26] on sample similarity graphs constructed from mRNA, miRNA, and DNA methylation data for subtypes and stage prediction[27]. Similarly,

MOGCN constructs sample similarity graphs from CNV, mRNA, and RPPA data in TCGA breast cancer and applies GCNs for subtype classification[28]. However, those approaches, either traditional non-GNN or sample-similarity-based GNN models, primarily focus on learning global representations at the sample level from the gene expression matrices to integrate omics data. As a result, they fail to fully utilize the interaction information and remain limited in capturing complex nonlinear dependencies across omics features, leaving substantial room for improvement in representation learning for disease and cancer subtypes prediction.

Cancer is not solely driven by single-gene aberrations but is regulated by complex molecular interaction networks, motivating the integration of interaction graphs into GNN-based omics frameworks where nodes represent genes, transcripts, proteins, or other regulatory entities and edges encode their interactions. Such designs enable GNNs to learn biologically meaningful local representations from molecular interaction topology, leading to improvement of disease prediction[10,16,29]. Recently, SynOmics extends GCN-based[26] architectures to intra- and inter-omic interaction graphs for cancer prognosis prediction[30]. Besides, Li et al. (2024) proposed a hybrid model in which MLPs encode global omics profiles and GCN or GAT[31] model molecular interaction networks, improving predictive performance for breast cancer subtype classification[32]. Nevertheless, current GNN-based models remain suboptimal. First, feature extraction is insufficient across multiple levels. MLP-based encoders tend to compress high-dimensional omics data toward task-specific representations, potentially

discarding informative variation. Meanwhile, standard message-passing in GNNs is largely confined to local neighborhoods, limiting the capture of long-range dependencies. In addition, most methods assume homogeneous node representations, ignoring intrinsic heterogeneity across omics types such as mRNA and proteins. This highlights the need for novel feature extractor that can more effectively represent both omics characteristics and interaction patterns. Second, representation fusion is often insufficient coordination. Simple concatenation may lead to unstable hierarchy representations and weak discrimination power in downstream tasks, underscoring the need for new fusion mechanism that can resolve conflicts and inconsistencies among multiple representations. Third, with the growing proteomic evidence for cancer classification, whether proteomics and their associated interaction networks are introduced to GNN-based models to enhance discriminative capacity remains a promising avenue for future research[33–36].

In this study, the GONet is proposed, a novel multi-level GNN framework for cancer classification by integrating multi-omics profiles (mRNAs, miRNAs, proteins) with interaction networks. GONet employs autoencoders to capture global representations, and applies Deep Graph Infomax (DGI)[37] to model local representations from molecular interaction networks. Considering the heterogeneous contribution across omics, GONet introduces an attention mechanism to generate omics representations that highlight of differential regulation of gene expression. Furthermore, a gated fusion module is designed in GONet to dynamically reconcile global, local, and omics-specific

representations, alleviating potential conflicts and improving integration consistency. Results from benchmark test and case studies demonstrated that GONet substantially improves predictive performance across diverse cancer datasets.

Results

GONet: A hierarchical graph neural network for multi-omics and molecular interaction integration

GONet is a modular, end-to-end framework that synergistically integrates matrix-based multi-omics data (e.g., transcriptomics and proteomics profiles) with graph-structured biomolecular interaction networks to derive biologically meaningful, low-dimensional representations for downstream classification tasks, including cancer staging and subtyping. Accordingly, the framework comprises three major parts: 1) dual-input: preprocessed multi-omics matrices and a curated interaction graph upon transcriptomes and proteomics; 2) model architecture: multi-level feature extraction followed by adaptive fusion; and 3) downstream prediction classification. A schematic overview of framework is presented in Fig. 1.

In the dual-input part, multi-omics matrices, including mRNA, miRNA, and proteomics profiles, are preprocessed into a unified matrix $X \in R^{n \times d}$, where n and d represent the number of samples and features, respectively. For each sample, the personalized molecular interaction graph $G = (V, E, H)$ is constructed based on the curated interactions from public databases, in which V as nodes represents molecules, E as

edges denotes interactions, and H stands for sample-specific molecular quantities (Methods).

In the model architecture part, three core modules are employed, feature extraction, feature fusion, and prediction. In feature extraction modules: three synergistic sub-modules are designed to generate complementary data representations. A) Global sample learning: Regarding the widespread application of autoencoders for dimensionality reduction in multi-omics data integration and addressing the limitation of MLP-based compression in capturing information-rich global representations, AE is applied to compress the input matrix X into a low-dimensional representation H_{global}, while the decoder reconstructs the input $\hat{X}$ by minimizing mean squared error (MSE) loss. B) Local molecular learning: Considering global representations overlook local patterns in molecular interaction networks, and that conventional GCN and GAT models suffer from over-smoothing and limited discriminative capacity, DGI is applied to maximize node-graph mutual information via the contrastive loss, generating local representations H_{local} from the molecular interaction graph G. C) Local omics learning: Addressing the lack of omics-specific information, a self-attention mechanism[38] (SAM) at omics-level adaptively weights inter-omics dependencies by learnable coefficients into H_{omics}. In feature fusion modules, an adaptive gated fusion mechanism[39] (GFM) aligns the complementary representations (H_{global}, H_{local}, and H_{omics}) from the three sub-modules. The GFM computes dynamic contribution weights for each sub-module and yield a unified low-dimensional representation H_{final},

minimizing redundancy and maximizing consistency of the fused representation. In prediction modules, H_{final} is passed through to a fully-connected classifier, which outputs class probabilities for cancer subtype or stage prediction (Fig. 1).

Fig 1. | Overview of the GONet framework. GONet integrates multi omics profiles (mRNA, miRNA, protein) with biomolecular interaction networks for cancer classification. **Data collection and graph construction**. Multi-omics datasets and curated molecular interaction networks are used to construct sample-specific molecular interaction graphs. **Model architecture.** Multi omics matrices serve as inputs to the global learning module (AE-based), while the sample-specific molecular interaction graphs are processed by the local learning module (DGI-based). In parallel, the local omics module captures inter-omics dependencies using attention mechanism. The resulting representations are fused via feature fusion module (gated mechanism-based) and passed to a prediction module for the downstream classification tasks.

Performance evaluation of GONet modules compared with previous GNN-based methods

GNN-based methods have been applied to cancer type and stage prediction using multi-omics data, including approaches with or without explicit modeling of interaction information, such as MOGONET[27], MOGCN[28], SynOmics[30], and Li's method[32]. As shown in Fig 2A and Table S4, MOGONET and MOGCN exhibit inconsistent performance across datasets, often performing best on their original benchmark datasets.

In contrast, SynOmics and Li's method demonstrate relatively more stable cross-dataset performance, suggesting that interaction information may be beneficial for effective cancer classification. However, SynOmics primarily considered the contribution of molecular interactions while underutilizing sample-level information, whereas Li's method integrates both local molecular interactions and global sample-level representations, yielding robust and generalizable multi-omics integration. Despite these advances, their representation learning and feature fusion strategies still leave room for further improvement.

To further analyze the contribution of each component, we compare GONet with Li et al.'s framework, which employs MLP-based global learning, GCN/GAT-based local learning, and concatenation-based fusion. In contrast, GONet adopts AE-based global encoding, DGI-based local learning, self-attention (SA)-based omics modeling, and gated fusion (GFM). Using the BRCA dataset from Li et al. (Li_dataset), all modules were evaluated independently under a unified classifier. For global learning, AE and MLP achieve comparable performance (AUROC: 0.9631±0.0047 vs. 0.9619±0.0108; Fig. 2B). For local learning, DGI significantly outperforms GCN and GAT (0.8558±0.0491 vs. 0.7536±0.0193 and 0.7483±0.0154; Fig. 2C). The SA-based omics module achieves an AUROC of 0.8077±0.039. For feature fusion, GFM slightly improves over concatenation (0.9663±0.0106 vs. 0.9578±0.0067; Fig. 2D). Overall, each module of GONet positively contributes to subtype classification, especially in local learning.

Performance of the full GONet framework was further evaluated for cancer subtype and stage classification in comparison with representative GNN-based methods on their original datasets using mRNA and miRNA data. As summarized in Fig 2E, As shown in Fig. 2E, GONet consistently outperforms all competitors across datasets. On Li et al.'s dataset, it achieves an AUROC of 0.9663, representing a 6.39% improvement over Li et al. (0.9082). Similar improvements are observed on MOGONET (0.9824 vs. 0.9520) and SynOmics (0.9849 vs. 0.9431), demonstrating strong cross-dataset generalization.

Next question is whether the prediction performance of GONet and the other GNN-based methods is comparable on the same omics datasets. CPTAC dataset was used for such evaluation, using mRNA and miRNA data across seven cancer types (BRCA, CCRCC, COAD, HNSCC, LUAD, LSCC, and PDAC). As shown in Fig 2E and Table S4, GONet consistently yields higher prediction performance than other GNN-based methods for cancer stage classification in the CPTAC datasets, with an average AUROC of 0.6420, corresponding to 16.41% relative improvement over Li's method, 39.78% over MOGONET, 29.15% over MOGCN, and 16.20% over SynOmics ($p<0.05$). Although performance gains are consistent, absolute AUROC values remain moderate, suggesting that dataset size and omics quality remain key limiting factors for multi-omics cancer prediction.

Fig 2 | Module-level comparison and performance evaluation of GONet against existing GNN-based models. **A)** Comparative predictive performance of

representative GNN-based methods across multiple public multi-omics cancer datasets. **B–D,** Module-specific evaluation of key architectural components in GONet on the BRCA subtype classification task using paired mRNA and miRNA data. **B)** *Global learning module.* Performance comparison of AE in GONet with a MLP baseline. **C)** *Local learning module.* Performance comparison of DGI with GCN and GAT baselines. **D)** *Feature fusion module.* Comparison between the adaptive GFM used in GONet and CAT. **E)** Generalization performance of GONet compared with representative GNN-based methods on original benchmark datasets (left) and CPTAC datasets (right).

Proteomics contributes to GONet for cancer classification

Unlike mRNA and miRNA data, which primarily capture transcriptional information, proteomics data directly reflects the functional state of gene expression[33,35,40]. Besides, large-scale resources, such as BioGRID[41] and STRING[42], accumulate extensive protein-protein interaction (PPI) information, providing inherent support for GNN-based modeling. These characteristics motivate the evaluation of proteomics as an alternative omics modality within the GONet for cancer classification prediction.

To systematically assess the applicability of GONet to proteomics data, a modality substitution experiment was conducted, where transcriptomic inputs (miRNA and mRNA) were replaced with proteomics data together with corresponding PPI networks across seven CPTAC cancer types.

As presented Fig 3A, GONet well accommodated the proteomics data, achieving performance comparable to transcriptomics-based models (average AUROC: 0.6360 vs. 0.6420). At the individual cancer-type level, the proteomics-based predictions achieved slightly higher AUROC values than transcriptomics-based predictions in five of seven cancer types (BRCA, CCRCC, LUAD, HNSCC and PDAC), whereas modestly lower performance was observed in COAD and LSCC. Overall, these results suggest that the proteomics data is a viable modality for GNN-based cancer stage prediction with GONet, at least in the CPTAC cohort.

An arising question concerns the relative contribution of individual omics to cancer stage prediction in GONet. As summarized in Fig 3B, proteomics alone yielded higher predictive performance among the three single-omics inputs, with an average AUROC of 0.6360, compared to 0.6038 for mRNA and 0.5721 for miRNA. At the individual dataset, proteomics-based predictions outperformed those derived from either mRNA or miRNA alone in five of the seven CPTAC cancer types. This suggests that proteomics appears a stronger discriminative signals for cancer staging within the GONet framework.

Finally, we evaluated whether the integration of three omics data could further improve cancer stage prediction in CPTAC. As shown in Fig 3B, the mean AUROC for tri-omics integration of miRNA, mRNA and proteomics in GONet was 0.6777, which is obviously higher than that derived by proteomics alone (0.6360; $p<0.05$) or transcriptomics (miRNA-mRNA:0.6420; $p<0.05$). These results demonstrate that

multi-omics integration provides complementary information, with proteomics contributing essential functional-level signals that enhance cancer stage prediction.

Fig 3 | Evaluation of proteomics integration in GONet. A) Comparison of transcriptomics-based (mRNA–miRNA) and proteomics-based inputs for cancer stage classification across CPTAC cohort. **B)** Performance of single-, dual- and tri-omics integration (mRNA, miRNA, protein), summarized by AUROC across CPTAC datasets.

Performance comparison of GONet and non-GNN methods for cancer classification

A wide range of non-GNN-based multi-omics integration methods, including RF[43], RF[43], SVM[44], LR[45], AE, MLP, HTML[22], and CLCLSA[21], have been applied to cancer type and stage classification. Given the strong performance of GONet compared with existing GNN-based methods, a systematic evaluation was conducted from three perspectives: i) comparison with existing non-GNN methods on their original datasets and on CPTAC, ii) evaluation under proteomics integration, and iii) global statistical comparison across all datasets and methods.

A total of 13 datasets were recruited, including those previously studied by HTML (COAD, ESCA, GBMLGG, SARC, STAD, STES, THCA, UCEC), CLCLSA (LGG, KIPAN), MOGONET, SynOmics, and Li et al. (Table S1). All datasets contained the mRNA and miRNA profiles with cancer stage or subtype labels. Twelves approaches, including GNN- and non-GNN-based methods, were applied onto each dataset, wth results summarized in Supplementary Data (Table S4). The performance can be

categorized into 3 circumstances, 1) due to unclear reason, all approaches achieved relatively poor performance on the 2 datasets HTML_GBMLGG and HTML_STAD, with AUROC ranging from 0.6297 to 0.7272 (the left panel of Fig 4A); 2) all approaches obtained near-saturated predictions on 3 datasets HTML_ESCA, HTML_STES, and HTML_KIPAN, with AUROC values exceeding 0.99 (the right panel of Fig 4A); and 3) the different approaches exhibited variable predictions on the remaining 8 datasets (the median panel of Fig 4A). GONet consistently outperforms all competing methods, achieving an average AUROC of 0.9495 versus 0.8810 for non-GNN methods. In contrast, GNN-based baselines achieve lower average performance (0.7978), indicating that existing GNN-based approaches are generally less effective than non-GNN baselines. These results strengthen our argument that improvements in representation learning and interaction modeling are critical for GNN-based multi-omics methods.

Performance was further evaluated on the CPTAC cohort, where seven representative non-GNN methods were applied for comparison. As shown in Fig. 4B, these methods achieve AUROC values ranging from 0.5095 to 0.5879, all significantly lower than GONet (0.6420; $p < 0.05$). Although overall performance remains moderate across all methods, likely due to limited sample size and task difficulty, GONet consistently achieves the best performance among both GNN and non-GNN approaches.

Giving the importance of proteomics, a question is raised if non-GNN-based approaches can effectively utilize tri-omics integration (mRNA, miRNA, and

proteomics) on CPTAC. As shown in Fig 4C, non-GNN-based approaches exhibited substantially lower predictive performance, with average AUROC values spanning 0.4780 to 0.5990 ($p<0.05$). Moreover, proteomics integration leads to inconsistent gains across methods. Notably, none of the methods exhibited consistent improvement across 7 cancer types, and 2 approaches (HTML and CLCLSA) showed performance declines (ΔAUROC= -0.0135 to -0.0301; Fig 4D). In contrast, GONet consistently benefited from proteomics integration in CPTAC datasets (7/7), achieving an average AUROC increase of 5.56% **(**ΔAUROC=0.0357**)**, demonstrating effective utilization of proteomic information.

Finally, a Friedman test across 27 experimental scenarios was conducted to statistically evaluate the advantages of GONet. As shown in Fig. 4E, the null hypothesis of equal performance is rejected ($p < 0.001$), confirming the statistically significant superiority of GONet across diverse evaluation scenarios.

Fig 4 | Systematic benchmarking of GONet performance across multi-omics cancer classification tasks. A) Performance comparison for bi-omics integration (mRNA-miRNA) between GONet and 11 competing methods across 14 datasets collected from previous studies grouped by task difficulty. **B)** Heatmap depicting AUROC values for bi-omics integration (mRNA-miRNA) between GONet and the 11 competing methods across seven CPTAC cancers. **C)** Heatmap showing AUROC values for tri-omics integration (mRNA, miRNA, and proteomics) between GONet and the 11 competing methods across seven CPTAC cancers. **D)** Heatmap illustrating the absolute AUROC

improvements (ΔAUROC) for each method after integrating proteomics data. **E)** Average ranks of all methods across multiple benchmarking scenarios, with lower ranks indicating superior overall performance.

Discussion

GNNs have recently emerged as a powerful approach for multi-omics data integration by incorporating molecular interaction networks, as exemplified by the Li et al. and the SynOmics framework[30,32]. However, as shown in Fig. 2A, systematic comparison of the current GNN-based approaches reveals that integration of interactions does not consistently yield improve performance, suggesting that effectiveness depends not only on the introduction of interaction information, but also on how efficiently they are leveraged. In complex cancer omics networks, conventional GCN and GAT models inherently behave as low-pass filters[46,47], aggregating local neighborhoods information, and readily suffering from over-smoothing during propagation processes, resulting in indistinguishable node representations and reduced discriminability[47,48]. To avoid the drawback, GONet innovatively incorporates DGI, which extracts more discriminative node representations from molecular interaction networks by maximizing mutual information between node-level representations and graph-level representations[37]. From an information-theoretic perspective, mutual information maximization captures global context and higher-order dependencies, and has been shown to improve embedding quality for downstream tasks[49,50]. Furthermore, DGI's contrastive learning framework distinguishes real node-graph pairs from corrupted ones, encouraging

robustness to noise and improving representation quality[51,52]. Module comparison experiments confirm that the DGI markedly improves the standard GCN or GAT, yielding superior classification performance (Fig. 2C). The improvement is consistent with prior reports in biological network representation learning, in which DGI enhances representation quality and downstream task performance in molecular-disease association networks[53], protein–protein interaction networks[54], and single-cell transcriptome-derived gene regulatory networks[55].

Proteins represent functional executors of biological processes, whereas transcripts primarily reflect upstream regulatory activity[56]. Accordingly, proteomics provides complementary functional and interaction-level signals, yet remains underutilized in existing approaches. Some studies integrated only protein expression data (moGCN with RPPA profiles[28]), while others included protein interaction networks without protein abundance, leaving synergistic modeling unexplored[32]. In this study, we simultaneously integrated protein abundance and associated interaction networks from the CPTAC cohort. Proteomes alone exhibited predictive power comparable to mRNA and miRNA, while integration with transcriptomic data further improves performance (Fig. 3A, B and Fig. 4C). Ablation analysis shows that neither abundance-only nor interaction-only signals yield strong performance (AUROC=0.6039 and 0.5587), whereas their combination yields a substantial gain (AUROC=0.6777; Fig. S5). These results indicate that predictive improvements arise from the synergistic integration of proteomic abundance and interaction structure. Notably, although incorporation of

proteome into GONet improves performance, the gain magnitude remains moderate. GONet still leaves substantial room for further improvement, primarily in two directions: more advanced model architectures, such as large language model-based approaches, and larger cohort sizes to enhance deep learning capacity and better capture mRNA and protein characteristics[57].

In summary, a modular GNN-based framework for integration of omics data with molecular interactions, GONet, was proposed and examined its capacity in cancer types and stages classification. GONet adopts a hierarchical representation learning strategy, AE-based global encoding, DGI-based local interaction modeling, attention-based modality weighting, and gated multi-level fusion. Extensive evaluations across multiple cancer cohorts and omics settings demonstrate that GONet achieves consistently robust performance compared with both GNN- and non-GNN-based methods. Particularly, GONet enables systematic joint modeling of proteomic abundance and interaction topology, with stable performance gains when proteomics data are involved. Overall, GONet has established a robust and generalizable multi-omics integration framework that provides a methodological foundation for disease prediction.

Methods

Data collection and preprocessing

Public available multi-omics datasets were collected, including the benchmark datasets from previous studies[21,22,27,30,32], and the CPTAC datasets downloaded from the

LinkomicsKB database[58], which provides uniformly processed multi-omics data. Detailed information on all datasets is summarized in Supplementary Table S1.

The benchmark datasets followed the original formats in previous literatures, whereas the CPTAC data were processed using the procedures described by wang et al. and Ji et al.[27,59]. Specifically, features with more than 20% missing values were removed, and remaining missing values were imputed using k-nearest neighbors (KNN). Besides, only samples with complete information of mRNA, miRNA, proteomics and corresponding clinical labels were remained for subsequent analyses.

Molecular interaction networks

Comprehensive molecular interaction networks in this study were curated by aggregation of public interaction repositories, including protein–protein interactions (PPIs) derived from BioGRID[41], STRING[42], and IID[60], mRNA-related regulatory interactions sourced from RISE[61], NPInter[62] and RNAInter[63], miRNA-related regulatory interactions retrieved from miRNet[64]. All interaction data were limited to human interactions, and nodes and edges involved such interactions were further filtered to remove incomplete or erroneous records. Molecular identifiers were standardized using UniProt IDs for proteins, HGNC symbols for mRNAs, and miRDB IDs for miRNAs.

Graph construction based on multi-omics interactions

For each patient sample s, a personalized molecular interaction graph G_s was constructed by integrating multi-omics profiles with curated interaction networks and defined as:

$$G_s = (V_s, E_s, H_s)$$

where V_s represents nodes with molecular entities (mRNAs, miRNAs, and proteins) detected in sample s, with v_i and v_j correspond to two nodes of V_s. E_s denotes edges derived from the curated interaction networks for sample s. Each edge $e_{ij} \in E_s$ between molecules v_i and v_j is defined as:

$$e_{ij} = \begin{cases} 1, & if \text{ an } interaction \text{ exists } between\, v_i \text{ and } v_j \\ 0, & otherwise \end{cases},$$

H_s corresponds to the normalized expression values of the respective molecular entities in sample s.

Construction of GONet

Global sample learning module

The AE used for global sample learning comprises an encoder and decoder.

Encoder processing. Let the original multi-omics feature matrix $X \in R^{n \times m}$ serve as input, where n represents the number of samples and m denotes the total number of molecular entities. A low dimensional global representation, $H_{global} \in R^{n \times d_g} (d_g \ll m)$, is compressed by encoder functioned $f_{enc}(\cdot)$:

$$H_{global} = f_{enc}(X)$$

where the f_{enc} consists of fully connected layers followed by LeakyReLU activations to capture non-linear patterns.

Decoder processing. The reconstructed representation $\hat{X}$ is generated from H_{global} by decoder functioned $f_{dec}(\cdot)$ to ensure that the compressed H_{global} retains the essential information of X. The reconstruction process is formulated as:

$$\hat{X} = f_{dec}\left(H_{global}\right)$$

where $\hat{X} \in R^{n\times m}$ represents the approximation of original matrix X. The decoder maintains a symmetric architecture with the encoder.

Reconstruction loss. The AE parameters are optimized by minimizing reconstruction loss L_{rec} between X and $\hat{X}$, defined as:

$$L_{\mathrm{rec}} = \| X - \hat{X} \|_2^2$$

where $\| \|_2^2$ is defined as the mean squared error (MSE).

Local molecular learning module

DGI model for local molecular learning is applied to learn high-quality local representations at two hierarchical scales: the node-level (individual molecules) and the graph-level (the entire molecular interaction network).

Node-level representation. For each sample s, node-level representations $H_{node} \in R^{|V_s|\times d_l}$ are learned vis graph convolutional encoder $GCN(\cdot)$ that aggregates

information from neighboring molecules in the sample-specific molecular interaction network G_s:

$$H_{node} = GCN(G_s)$$

where $|V_s|$ represents the number of molecule nodes in sample s, and d_l denotes the dimensionality of node representation. Each row $h_v \in H_{node}$ corresponds to a d_l-dimensional embedding of molecule v.

Graph-level representation. The graph-level representation $H_{local} \in R^{2d_l}$ is obtained by aggregating H_{node} with a dual-pooling strategy (mean and max pooling) to capture the global topology of the molecular network:

$$H_{local} = \left[\frac{1}{|V_s|}\sum_{v \in V_s} vh_v\right] \oplus \left[\max_{v \in V_s} h_v\right]$$

where $\oplus$ denotes vector concatenations, and the left and right part indicate pooling strategies to generate $2d_l$-dimensional vector for sample s.

Maximization of Mutual Information. To ensure that node-level representations encode global topology information, a discriminator D is introduced to maximize the mutual information between H_{node} and H_{local}, which distinguishes between real node-graph pairs (h_v, H_{local}) from the true interaction graph G_s, and corrupted pairs $(\tilde{h}_v, H_{local})$, as described below:

$$L_{DGI} = -\frac{1}{|V_s|}\sum_{v \in V_s}\left[logD(h_v, H_{local}) + log\left(1 - D\left(\widetilde{h_v}, H_{local}\right)\right)\right]$$

where $\tilde{h}_v$ represents the corrupted node representation obtained from a corrupted graph with randomly shuffle nodes.

Local omics learning module

The local omics learning module aggregates node-level representations into omics-specific representations and integrates information from different omics through an adaptive attention mechanism.

Omics-specific representation aggregation. To reduce noise and capture omics-specific signatures, omics-specific representations H_m for each omics modality m are generated by averaging the node-level representations of its constituent molecules V_m:

$$H_m = \frac{1}{|V_m|} \sum_{v \in V_m} h_v$$

where V_m represents the set of nodes belonging to modality m. The representations of all omics modalities are then stacked as:

$$H_{stack} = \begin{bmatrix} H_1 \\ H_2 \\ \vdots \\ H_{|M|} \end{bmatrix}, \qquad H_{stack} \in R^{|M| \times d_l}.$$

where $|M|$ is the number of omics.

Self-attention mechanism. To adaptively weight the contributions of different omics modalities, the attention weights A are computed as follows:

$$A = softmax\left(\frac{\sigma(H_{stack}W_Q)(H_{stack}W_k)^{\top}}{\sqrt{d_k}}\right),$$

where W_Q and W_K are learnable weight matrices and d_k is the attention dimension. The fused omics-level representation H_{omics} is obtained by weighting H_{stack} with A:

$$H_{omics} = A(H_{stack}W_V).$$

Contrastive loss. To optimize the omics module and ensure that the learned representations H_{omics} are discriminative, a supervised contrastive loss L_{sup} is minimized to encourage sample with same label y to have similar embeddings:

$$L_{sup} = \text{SupConLoss}(H_{\text{omics}}, y; \tau),$$

where τ represents the temperature hyperparameter controlling the scale of the loss.

Feature fusion module

The feature fusion module integrates global, local, and omics representations through representation alignment followed by adaptive fusion to synthesize multi-level information.

Representation alignment. Given the different feature spaces and dimensions of H_{global} H_{local} and H_{omics}, the three representations are projected into aligned representations Z_{mod} with a common dimension d_f by a fully-connected layer followed by a LeakyReLU activation:

$$\mathrm{Z}_{mod} = \text{LeakyReLU}(W_{mod}H_{\text{mod}} + b_{mod}), mod \in \{global, local, omics\}$$

where W_{mod} and b_{mod} are learnable parameters.

Representation fusion. An adaptive gated mechanism $f_{\text{Gate}}(\cdot)$ is employed to learn relative importance weights α_{mod} of each representation:

$$\alpha_{mod} = [\alpha_{global}, \alpha_{local}, \alpha_{omics}] = f_{\text{Gate}}([\mathrm{Z}_{global} \oplus \mathrm{Z}_{local} \oplus \mathrm{Z}_{omics}])$$

$$\sum_{mod} \alpha_{mod} = 1$$

where ⊕ denotes concatenation, and the gating function is implemented as a softmax operation to normalize the importance weights across the three modules. The final fused representation H_{final} is defined as the convex combination of the Z_{mod}:

$$H_{final} = \sum_{mod} \alpha_{mod} Z_{mod}, \qquad H_{\text{final}} \in R^{n \times d_f}.$$

Prediction module

The prediction module performs cancer stage or subtype classification based on H_{final}, and the prediction performance is assessed using focal loss[65].

Classification prediction. The fused representation H_{final} is fed into a fully-connected layer with learnable parameters W_c and b_c, followed by a $softmax$ function to predict cancer stages or subtypes. The predicted probability vector p is computed as:

$$p = softmax(W_c H_{final} + b_c)$$

Focal loss. Focal loss is used as the classification loss L_{cls}, which measures discrepancy between the predicted probability p and the true class $y \in \{1, \dots, C\}$:

$$L_{cls} = -\alpha(1 - p_y)^{\gamma} log(p_y)$$

where p_y represents the predicted probability for true label y, and α and γ are learnable hyperparameter controlling class weighting and focusing strength, respectively.

Total loss function

The GONet framework is trained by minimizing a joint multi-objective loss function:

$$L_{total} = \lambda_{rec}L_{rec} + \lambda_{DGI}L_{DGI} + \lambda_{sup}L_{sup} + \lambda_{cls}L_{cls} + \lambda_{reg}L_{reg}$$

where L_{reg} is a regularization item to prevent overfitting, and trade-off hyperparameters $\{\lambda_{\text{rec}}, \lambda_{\text{DGI}}, \lambda_{\text{contra}}, \lambda_{\text{cls}}, and\ \lambda_{\text{reg}}\}$ control the relative contribution of each loss items.

Data and Code Availability

The source code of GONet is publicly available at https://github.com/EayonYang/GONet, including implementation, preprocessing, training, and evaluation scripts. The repository is released under an MIT license. All datasets used in this study are publicly available from their original sources: Li's dataset (https://github.com/NabaviLab/Multimodal-GNN-for-Cancer-Subtype-Clasification.git), MOGONET (https://github.com/txWang/MOGONET.git), SynOmics (https://github.com/compbiolabucf/SynOmics/tree/main), HTML dataset (https://drive.google.com/drive/folders/1_tJ2ekhTmWp7ZcRVjUVGx0cqGMRKEhNo), CLCLSA (https://github.com/MIILab-MTU/CLCLSA/tree/main), and CPTAC (https://kb.linkedomics.org/download). All datasets are appropriately cited and described in the manuscript. A DOME-ML annotation has been completed to support standardized machine learning reporting, available at https://registry.dome-ml.org/review/25n5mvzy2v.

Conflict of interest

None declared.

Funding

The work was supported by the National Key Research and Development Program of China (grant no.: 2023YFC3402900).

Reference

1. Bray, F. *et al.* Global cancer statistics 2022: GLOBOCAN estimates of incidence and mortality worldwide for 36 cancers in 185 countries. *CA. Cancer J. Clin.* **74**, 229–263 (2024).
2. Li, Y., Dou, Y. & V, L. F. D. Proteogenomic data and resources for pan-cancer analysis. *Cancer Cell* **41**, 1397–1406 (2023).
3. The ICGC/TCGA Pan-Cancer Analysis of Whole Genomes Consortium *et al.* Pan-cancer analysis of whole genomes. *Nature* **578**, 82–93 (2020).
4. Manzoni, C. *et al.* Genome, transcriptome and proteome: the rise of omics data and their integration in biomedical sciences. *Brief Bioinform* **1;19(2):286-302**, (2018).
5. Chakraborty, S., Sharma, G., Karmakar, S. & Banerjee, S. Multi-OMICS approaches in cancer biology: New era in cancer therapy. *Biochim. Biophys. Acta BBA - Mol. Basis Dis.* **1870**, 167120 (2024).
6. Huang, C.-Y. *et al.* Integrating comprehensive genomic profiling in the management of oncology patients: applications and challenges in Taiwan. *Biomed. J.* 100851 (2025).
7. A, B., S, D. & Mandal R ,et al. Increased heterogeneity in expression of genes associated with cancer progression and drug resistance. *Transl. Oncol.* 41.
8. Ferlier, T. & Coulouarn, C. Regulation of Gene Expression in Cancer-An Overview. *Cells* **15;11(24):4058**, (2022).

9. Hanahan, D. Hallmarks of Cancer: New Dimensions. *Cancer Discov.* **12**, 31–46 (2022).

10. Li, W., Ballard, J., Zhao, Y. & Long, Q. Knowledge-guided learning methods for integrative analysis of multi-omics data. *Comput. Struct. Biotechnol. J.* **23**, 1945–1950 (2024).

11. CRICK,FRANCIS. Central Dogma of Molecular Biology. *Nature* (1970).

12. Pan, Y., Lei, X. & Zhang, Y. Association predictions of genomics, proteinomics, transcriptomics, microbiome, metabolomics, pathomics, radiomics, drug, symptoms, environment factor, and disease networks: A comprehensive approach. *Med. Res. Rev.* **42**, 441–461 (2022).

13. Panditrao, G., Bhowmick, R., Meena, C. & Sarkar, R. R. Emerging landscape of molecular interaction networks: Opportunities, challenges and prospects. *J. Biosci.* **47**, 24 (2022).

14. Luo, Y., Zhao, C. & Chen, F. Multiomics Research: Principles and Challenges in Integrated Analysis. *BioDesign Res.* **6**, 0059 (2024).

15. Subramanian, I., Verma, S., Kumar, S., Jere, A. & Anamika, K. Multi-omics Data Integration, Interpretation, and Its Application. *Bioinforma. Biol. Insights* **14**, 117793221989905 (2020).

16. Thapa, K., Kinali, M., Pei, S., Luna, A. & Babur, Ö. Strategies to include prior knowledge in omics analysis with deep neural networks. *Patterns* **6**, 101203 (2025).

17. Liu, J. *et al.* Challenges in AI-driven Biomedical Multimodal Data Fusion and Analysis. *Genomics Proteomics Bioinformatics* **23**, qzaf011 (2025).

18. Ballard, J. L., Wang, Z., Li, W., Shen, L. & Long, Q. Deep learning-based approaches for multi-omics data integration and analysis. *BioData Min.* **17**, 38 (2024).

19. Cai, Z. Machine learning for multi-omics data integration in cancer. *OPEN ACCESS*.

20. Yao, X. *et al.* MOCAT: multi-omics integration with auxiliary classifiers enhanced autoencoder. *BioData Min.* **17**, (2024).

21. Zhao, C. *et al.* CLCLSA: Cross-omics linked embedding with contrastive learning and self attention for integration with incomplete multi-omics data. *Comput. Biol. Med.* **170**, 108058 (2024).

22. Lu, Y. *et al.* Multiomics dynamic learning enables personalized diagnosis and prognosis for pancancer and cancer subtypes.

23. Valous, N. A., Popp, F., Zörnig, I., Jäger, D. & Charoentong, P. Graph machine learning for integrated multi-omics analysis. *Br. J. Cancer* **131**, 205–211 (2024).

24. Khoshraftar, S. & An, A. A Survey on Graph Representation Learning Methods. *ACM Trans. Intell. Syst. Technol.* **15**, 1–55 (2024).

25. Khemani, B., Patil, S., Kotecha, K. & Tanwar, S. A review of graph neural networks: concepts, architectures, techniques, challenges, datasets, applications, and future directions. *J. Big Data* **11**, 18 (2024).

26. Kipf, T. N. & Welling, M. Semi-Supervised Classification with Graph Convolutional Networks. Preprint at https://doi.org/10.48550/ARXIV.1609.02907 (2016).

27. Wang, T. *et al.* MOGONET integrates multi-omics data using graph convolutional networks allowing patient classification and biomarker identification. *Nat. Commun.* **12**, 3445 (2021).

28. Li, X., Ma, J. & Leng, L. MoGCN: a multi-omics integration method based on graph convolutional network for cancer subtype analysis. *Front. Genet.* **13**, 806842 (2022).

29. Xiao, S., Lin, H., Wang, C., Wang, S. & Rajapakse, J. C. Graph Neural Networks With Multiple Prior Knowledge for Multi-Omics Data Analysis. *IEEE J. Biomed. Health Inform.* **27**, 4591-4600, (2023).

30. Alif, M. N. & Zhang, W. SynOmics: integrating multi-omics data through feature interaction networks.

31. Liu, Z. & Zhou, J. Graph Attention Networks. in *Introduction to Graph Neural Networks* 39–41 (Springer International Publishing, Cham, 2020).

32. Li, B. & Nabavi, S. A multimodal graph neural network framework for cancer molecular subtype classification. *BMC Bioinformatics* **25**, (2024).

33. Kustatscher, G., Collins, T. & C, G. A. Understudied proteins: opportunities and challenges for functional proteomics. *Nat. Methods* **19**, 774–779 (2022).

34. Dong, L. *et al.* Proteogenomic characterization identifies clinically relevant subgroups of intrahepatic cholangiocarcinoma. *Cancer Cell* **40**, 70-87.e15 (2022).

35. Mun, D.-G. *et al.* Proteogenomic Characterization of Human Early-Onset Gastric Cancer. *Cancer Cell* **35**, 111-124.e10 (2019).

36. Gao, Q. *et al.* Integrated Proteogenomic Characterization of HBV-Related Hepatocellular Carcinoma. *Cell* **179**, 561-577.e22 (2019).

37. Veličković, P., Fedus, W. & L, H. W. Deep graph infomax. (2018).

38. Attention is All You Need.

39. Arevalo, J., Solorio, T. & Montes-y-Gómez, M. Gated multimodal units for information fusion. (2017).

40. R, S. S. An introduction to mass spectrometry-based proteomics. *J. Proteome Res.* **22**, 2151–2171 (2023).

41. Oughtred, R. *et al.* The BioGRID database: A comprehensive biomedical resource of curated protein, genetic, and chemical interactions. *Protein Sci* https://doi.org/10.1002/pro.3978. (2021).

42. Szklarczyk, D. *et al.* The STRING database in 2023: protein-protein association networks and functional enrichment analyses for any sequenced genome of interest. *Nucleic Acids Res* **6;51(D1):D638-D646**, (2023).

43. Breiman, L. Random Forests. *Mach. Learn.* **45**, 5–32 (2001).

44. Suykens, J. A. K. & Vandewalle, J. Least Squares Support Vector Machine Classifiers. *Neural Process. Lett.* **9**, 293–300 (1999).

45. Buckley, J. & James, I. Linear regression with censored data. *Biometrika* **66**, 429–436 (1979).

46. Wu, Z., Pan, S., Long, G., Jiang, J. & Zhang, C. Beyond Low-pass Filtering: Graph Convolutional Networks with Automatic Filtering. https://doi.org/10.48550/ARXIV.2107.04755 (2021).

47. Li, Q., Han, Z. & Wu, X.-M. Deeper Insights into Graph Convolutional Networks for Semi-Supervised Learning. Preprint at https://doi.org/10.48550/ARXIV.1801.07606 (2018).

48. Wu, Y., Hu, X., Fan, X., Ma, W. & Gao, Q. Learning Data-Driven Propagation Mechanism for Graph Neural Network. *Electronics* **12**, 46 (2022).

49. Sun, F.-Y., Hoffmann, J., Verma, V. & Tang, J. InfoGraph: Unsupervised and Semi-supervised Graph-Level Representation Learning via Mutual Information Maximization. Preprint at https://doi.org/10.48550/ARXIV.1908.01000 (2019).

50. Hjelm, R. D. *et al.* Learning deep representations by mutual information estimation and maximization. Preprint at https://doi.org/10.48550/ARXIV.1808.06670 (2018).

51. Hassani, K. & Khasahmadi, A. H. Contrastive Multi-View Representation Learning on Graphs. Preprint at https://doi.org/10.48550/ARXIV.2006.05582 (2020).

52. You, Y. *et al.* Graph Contrastive Learning with Augmentations. Preprint at https://doi.org/10.48550/ARXIV.2010.13902 (2020).

53. Zhang, L., Dong, X., Jia, S. & Zhang, J. Node2Vec-DGI-EL: A Hierarchical Graph Representation Learning Model for Ingredient-Disease Association Prediction. *Bioinforma. Adv.* vbaf216 (2025).

54. Yan, R., Islam, M. T. & Xing, L. Deep representation learning of protein-protein interaction networks for enhanced pattern discovery. *Sci. Adv.* **10**, eadq4324 (2024).

55. Wang, P. *et al.* Deciphering driver regulators of cell fate decisions from single-cell transcriptomics data with CEFCON. *Nat. Commun.* **14**, 8459 (2023).

56. Savage, S. R. *et al.* Pan-cancer proteogenomics expands the landscape of therapeutic targets. *Cell* **187**, 4389-4407.e15 (2024).

57. Dong, Y. *et al.* Deep learning methods may not outperform other machine learning methods on analyzing genomic studies. *Front. Genet.* **13**, 992070 (2022).

58. Liao, Y. *et al.* A proteogenomics data-driven knowledge base of human cancer. *Cell Syst* https://doi.org/10.1016/j.cels.2023.07.007. (2023) doi:10.1016/j.cels.2023.07.007.

59. Ji, Y., Dutta, P. & Davuluri, R. Deep multi-omics integration by learning correlation-maximizing representation identifies prognostically stratified cancer subtypes. *Bioinforma. Adv.* **3**, vbad075 (2023).

60. Pastrello, C., Kotlyar, M. & Jurisica, I. Informed Use of Protein–Protein Interaction Data: A Focus on the Integrated Interactions Database (IID). *Methods Mol. Biol.* **2074**, (2020).

61. Gong, J. *et al.* RISE: a database of RNA interactome from sequencing experiments. *Nucleic Acids Res* **4;46(D1):D194-D201**, (2018).

62. Zheng, Y. *et al.* NPInter v5.0: ncRNA interaction database in a new era. *Nucleic Acids Res.* **51**, D232–D239 (2023).

63. Kang, J. *et al.* RNAInter v4.0: RNA interactome repository with redefined confidence scoring system and improved accessibility. *Nucleic Acids Res* **7;50(D1):D326-D332**, (2022).

64. Chang, L., Zhou, G., Soufan, O. & Xia, J. miRNet 2.0: network-based visual analytics for miRNA functional analysis and systems biology. *Nucleic Acids Res.* **48**, W244–W251 (2020).

65. Lin, T.-Y., Goyal, P., Girshick, R., He, K. & Dollár, P. Focal Loss for Dense Object Detection. *Art* https://doi.org/10.48550/arXiv.1708.02002. (2017).

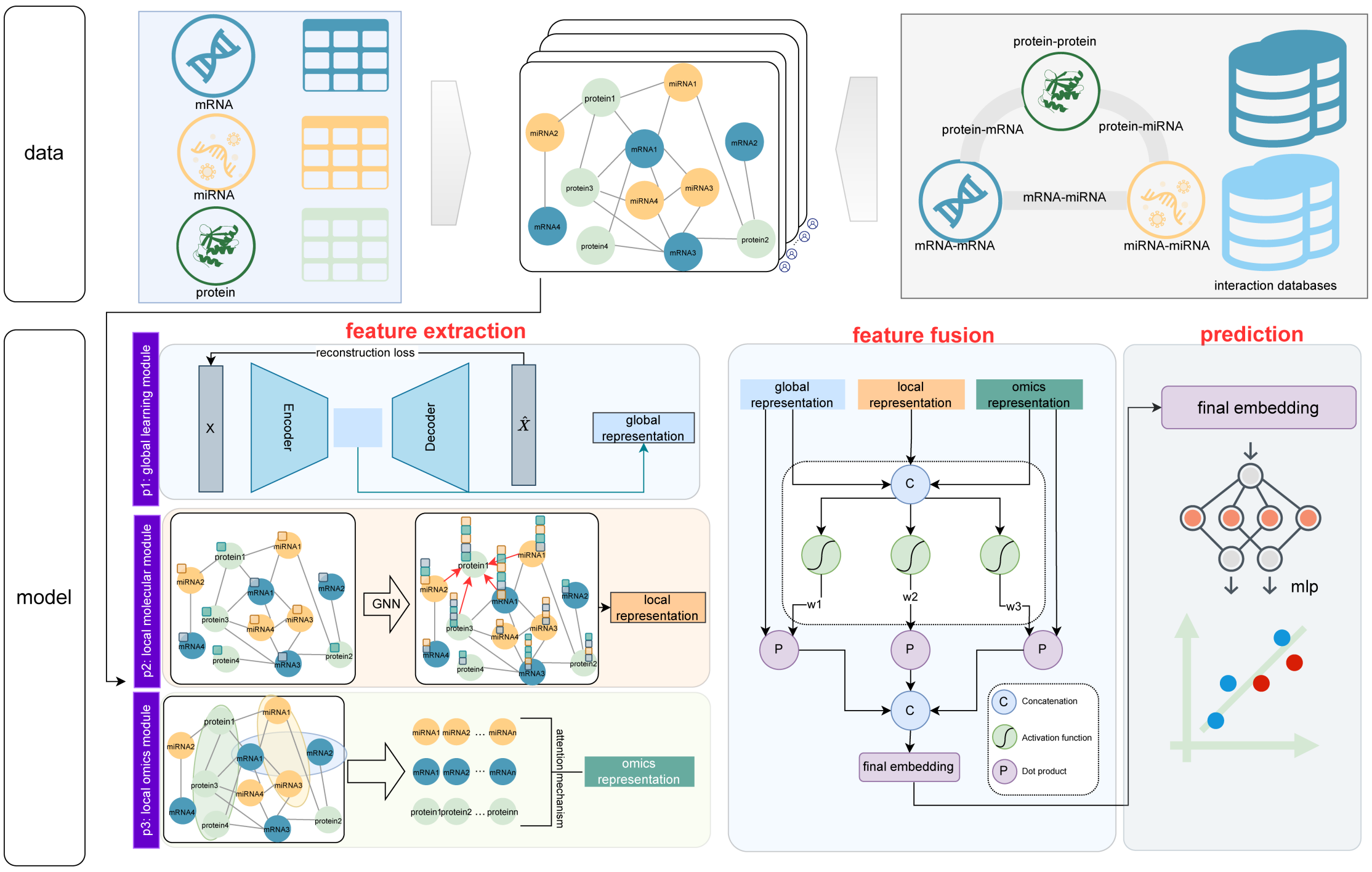

Fig 1. | Overview of the GONet framework. GONet integrates multi omics profiles (mRNA, miRNA, protein) with biomolecular interaction networks for cancer classification. **Data collection and graph construction**. Multi-omics datasets and curated molecular interaction networks are used to construct sample-specific molecular interaction graphs. **Model architecture.** Multi omics matrices serve as inputs to the global learning module (AE-based), while the sample-specific molecular interaction graphs are processed by the local learning module (DGI-based). In parallel, the local omics module captures inter-omics dependencies using attention mechanism. The resulting representations are fused via feature fusion module (gated mechanism-based) and passed to a prediction module for the downstream classification tasks.

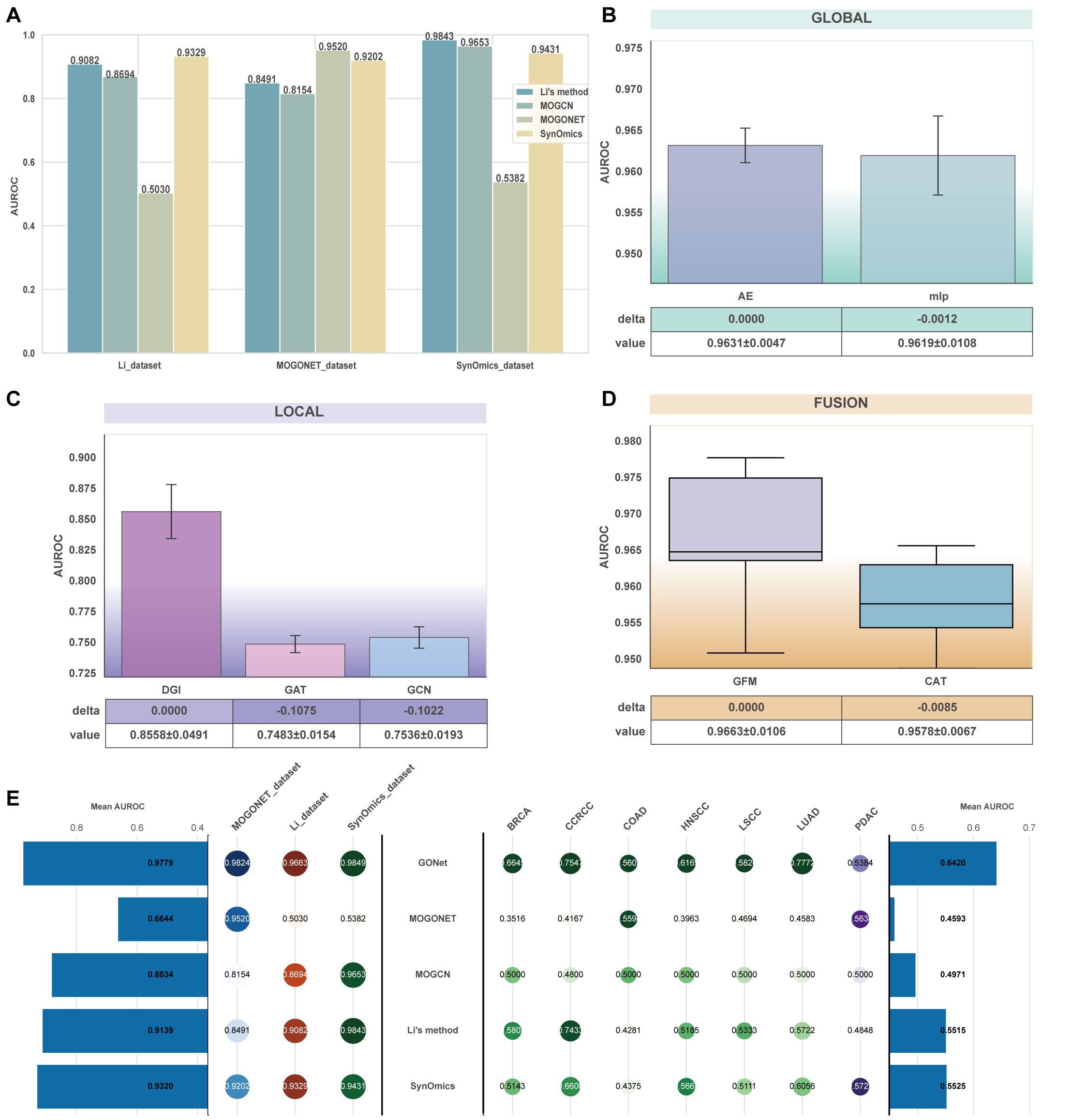

Fig 2 | Module-level comparison and performance evaluation of GONet against existing GNN-based models. **A)** Comparative predictive performance of representative GNN-based methods across multiple public multi-omics cancer datasets. **B–D,** Module-specific evaluation of key architectural components in GONet on the BRCA subtype classification task using paired mRNA and miRNA data. **B)** *Global learning module.* Performance comparison of AE in GONet with a MLP baseline. **C)** *Local learning module.* Performance comparison of DGI with GCN and GAT baselines. **D)** *Feature fusion module.* Comparison between the adaptive GFM used in GONet and CAT. **E)** Generalization performance of GONet compared with representative GNN-based methods on original benchmark datasets (left) and CPTAC datasets (right).

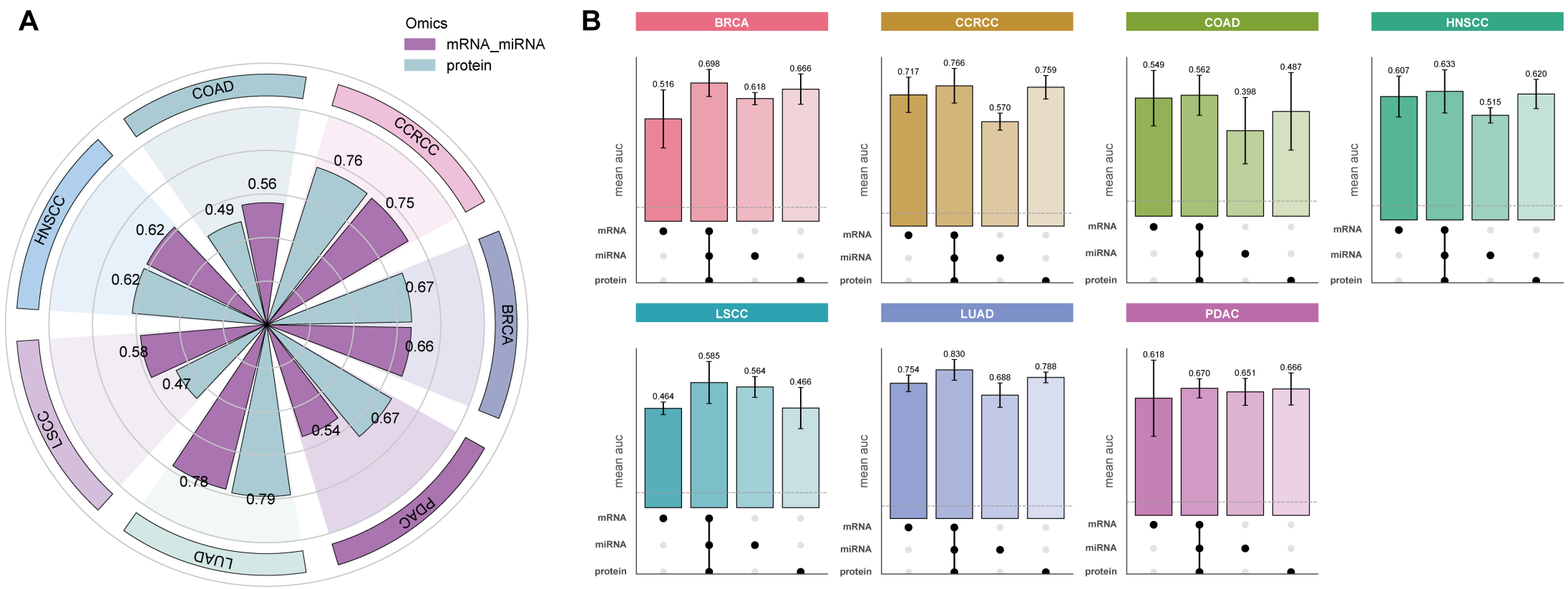

Fig 3 | Evaluation of proteomics integration in GONet. A) Comparison of transcriptomics-based (mRNA–miRNA) and proteomics-based inputs for cancer stage classification across CPTAC cohort. **B)** Performance of single-, dual- and tri-omics integration (mRNA, miRNA, protein), summarized by AUROC across CPTAC datasets.

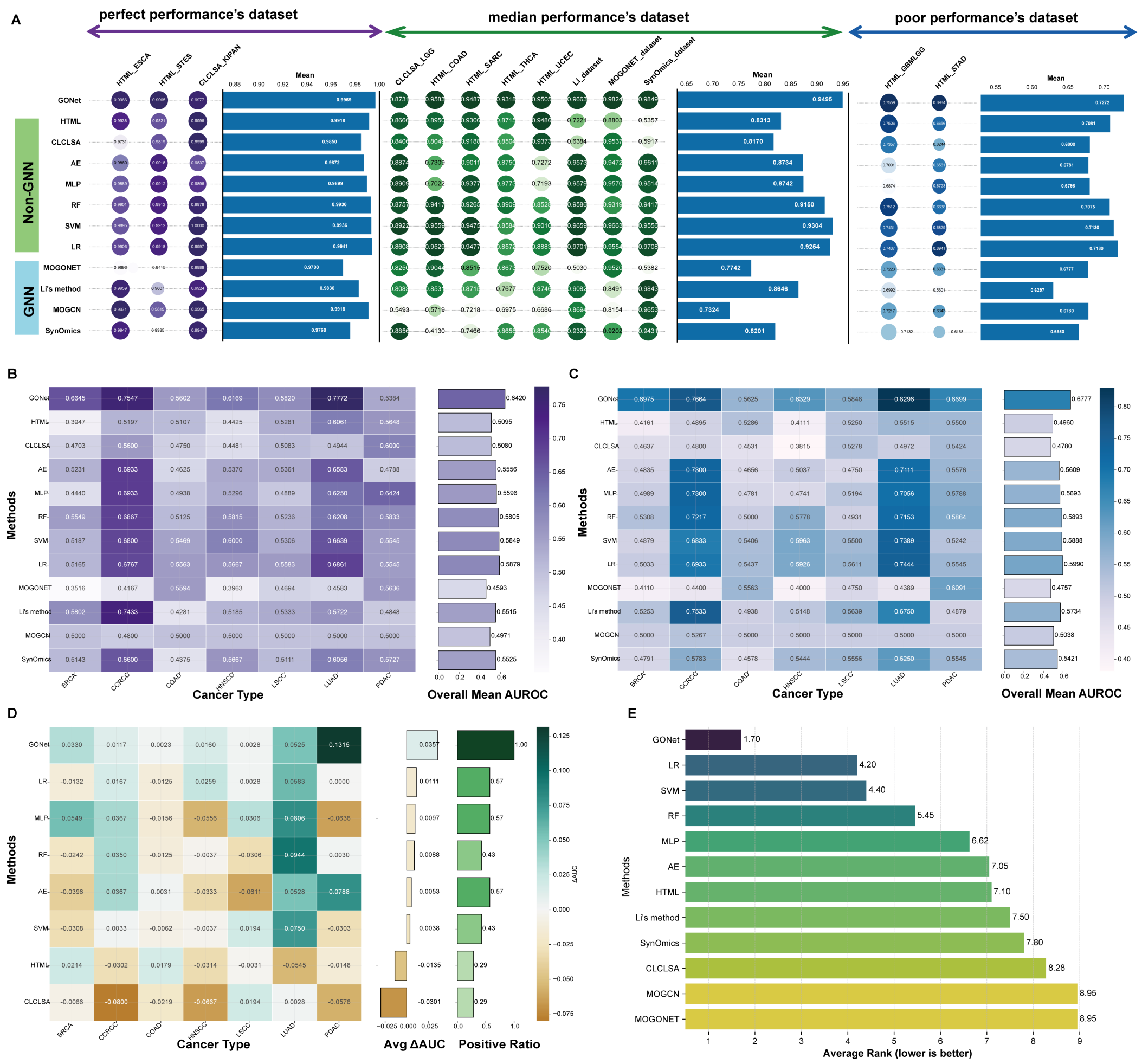

Fig 4 | Systematic benchmarking of GONet performance across multi-omics cancer classification tasks. A) Performance comparison for bi-omics integration (mRNA-miRNA) between GONet and 11 competing methods across 14 datasets collected from previous studies grouped by task difficulty. **B)** Heatmap depicting AUROC values for bi-omics integration (mRNA-miRNA) between GONet and the 11 competing methods across seven CPTAC cancers. **C)** Heatmap showing AUROC values for tri-omics integration (mRNA, miRNA, and proteomics) between GONet and the 11 competing methods across seven CPTAC cancers. **D)** Heatmap illustrating the absolute AUROC improvements (ΔAUROC) for each method after integrating proteomics data. **E)** Average ranks of all methods across multiple benchmarking scenarios, with lower ranks indicating superior overall performance.

Fig 1. | Overview of the GONet framework. GONet integrates multi omics profiles (mRNA, miRNA, protein) with biomolecular interaction networks for cancer

Click here to access/download;Figure;Fig1.tif

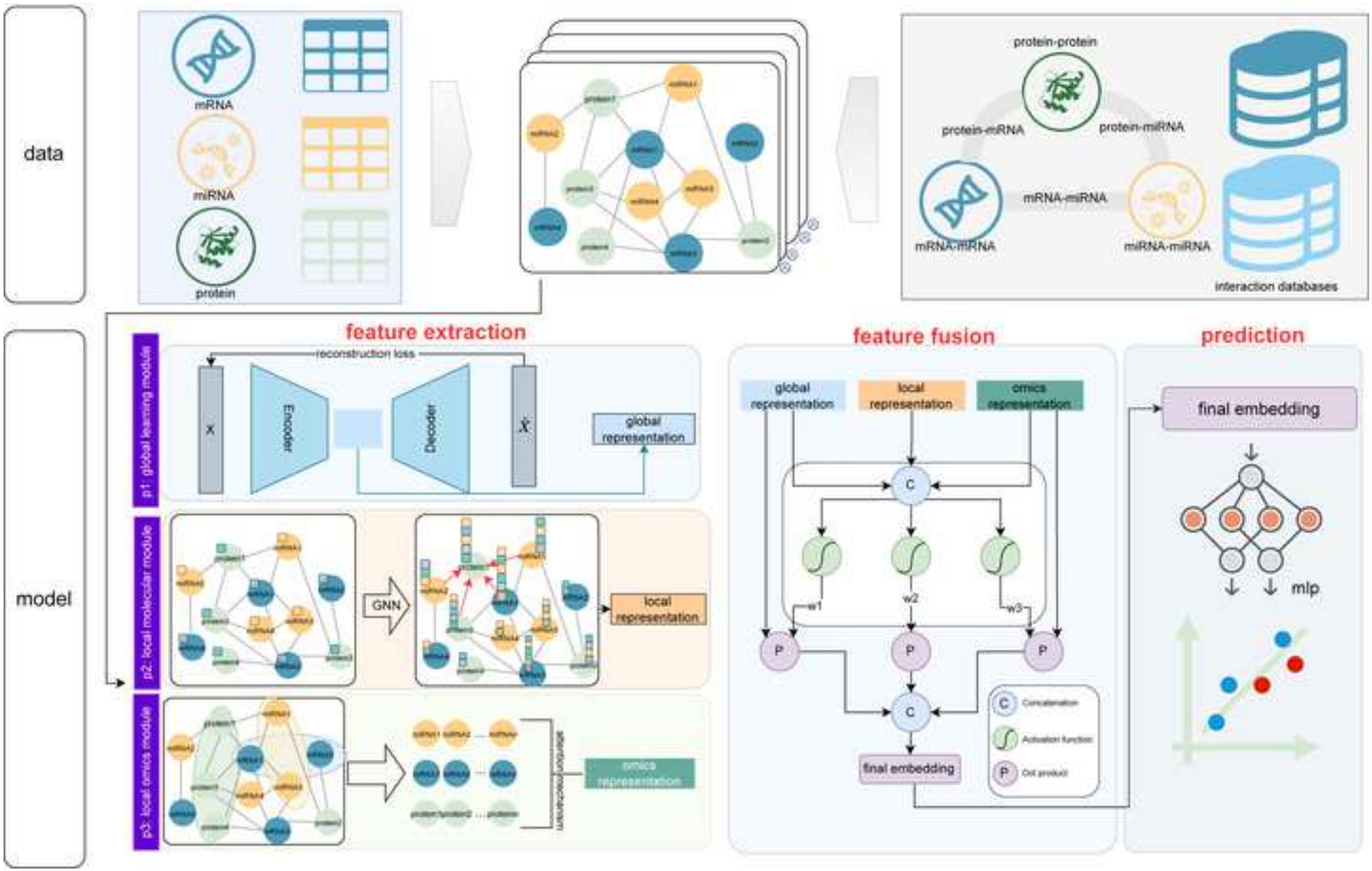

Fig 2 | Module-level comparison and performance evaluation of GONet against existing GNN-based models. A) Comparative

Click here to access/download;Figure;Fig2.tif

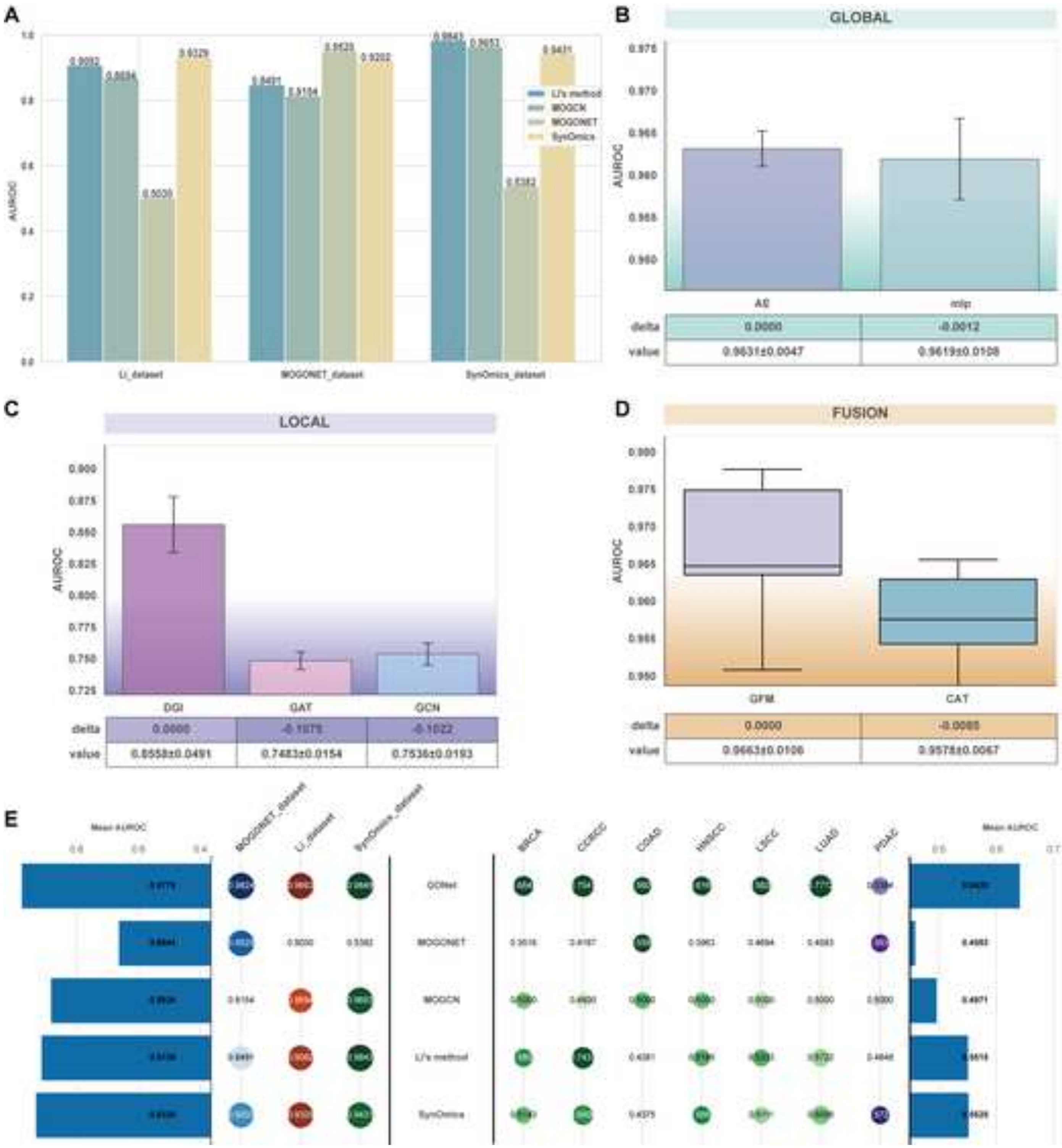

Fig 3 | Evaluation of proteomics integration in GONet. A) Comparison of transcriptomics-based (mRNA-miRNA) and proteomics-based inputs for cancer stage

Click here to access/download;Figure;Fig3.tif

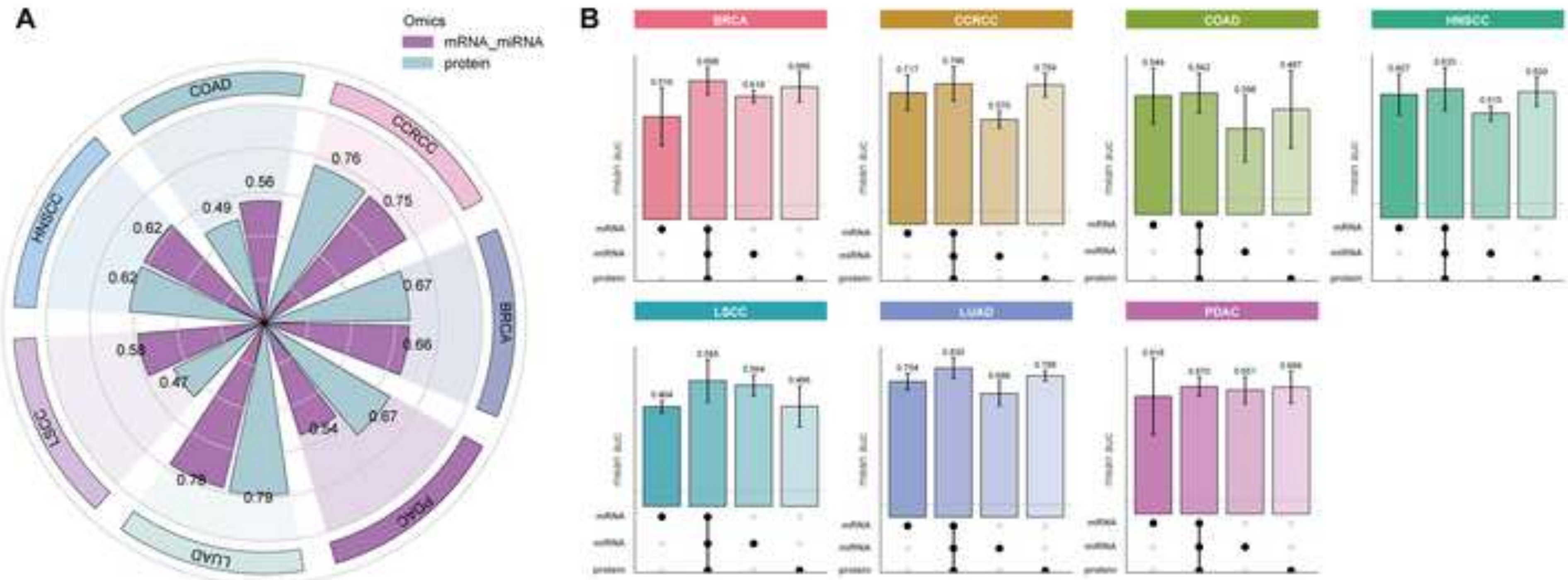

Fig 4 | Systematic benchmarking of GONet performance across multi-omics cancer classification tasks. A) Performance comparison for bi-omics integration (mRNA-

Click here to access/download;Figure;Fig4.tif

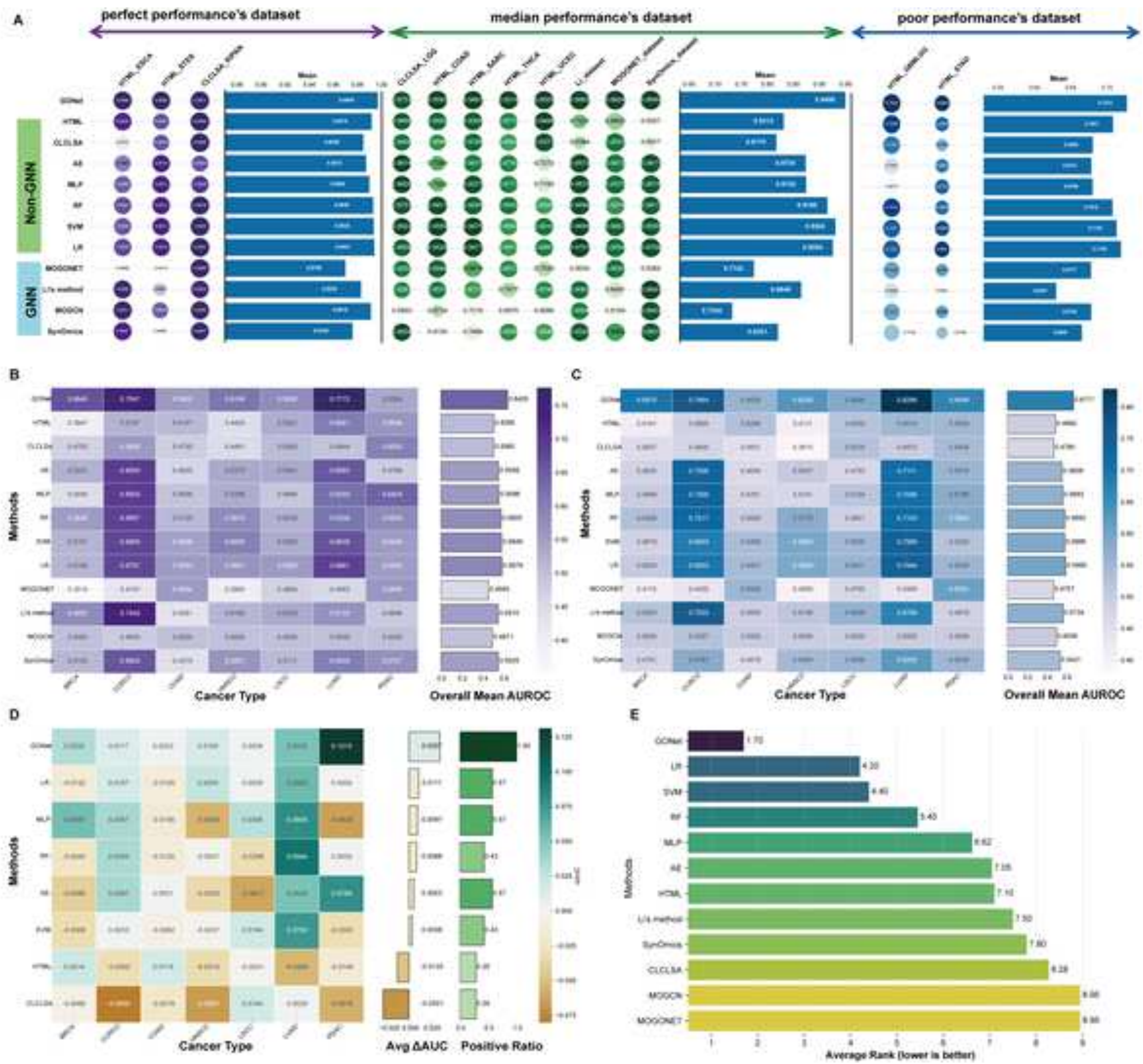

supplementary

Click here to access/download
Supplementary Material
supplementary-updated.pdf
