# Supplementary material for: GONet: a molecular interaction-guided graph learning framework for multi-omics cancer classification
Source: Gigascience. 2026 Oct 22;15:giag097. doi: 10.1093/gigascience/giag097 (PMC13741247; doi:10.1093/gigascience/giag097)
Supplement: Supplementary file 3 [file giag097_giga-d-26-00172_revision_1.pdf]

GigaScience

GONet: A Molecular Interaction-Guided Graph Learning Framework for Multi-Omics Cancer Classification

--Manuscript Draft--

**Manuscript Number:**	GIGA-D-26-00172R1
**Full Title:**	GONet: A Molecular Interaction-Guided Graph Learning Framework for Multi-Omics Cancer Classification
**Article Type:**	Research
**Funding Information:**	Key Technologies Research and Development Program (2023YFC3402900) — Dr. Guixue Hou
**Abstract:**	Integrating multi-omics profiles with molecular interaction networks enables biologically informed representation learning by combining molecular measurements with prior biological knowledge. Although graph neural networks (GNNs) are well suited for modeling molecular interaction networks, existing multi-omics methods often rely on shallow representation learning or incomplete fusion strategies, which fail to fully capture complementary information and complex dependencies across heterogeneous omics layers, resulting in suboptimal performance and limited generalizability in cancer classification tasks.<br>Here, we propose GONet, a hierarchical GNN framework that integrates omics expression profiles, including mRNA, miRNA, and protein data, with molecular interaction networks, where nodes represent omics features and edges represent molecular interactions. GONet learns global omics representations via an autoencoder, captures local structural dependencies using Deep Graph Infomax (DGI), and enhances omics-specific representations through an attention mechanism. A gated fusion module further enables adaptive integration of heterogeneous features, improving cancer subtype and stage classification.<br>GONet was systematically evaluated on 13 publicly available benchmark datasets used in prior studies, covering 10 cancer types, ensuring fair comparison across methods. Across mRNA and miRNA datasets, GONet achieved an average AUROC of 0.9262, outperforming representative GNN- and non-GNN-based methods by 13.77% and 6.33%, respectively. On an independent CPTAC cohort spanning seven cancer types, it improved performance by 25.22% and 13.91%, respectively. Incorporating proteomics further improved cancer stage classification by 5.56%, demonstrating complementary predictive value. Ablation studies confirmed the effectiveness of the DGI-based module. Overall, GONet provides a unified framework for biologically informed multi-omics graph representation learning and cancer classification.
**Corresponding Author:**	Siqi Liu, PhD<br>BGI-Shenzhen: BGI Group<br>Shenzhen, Guangdong CHINA
**Corresponding Author Secondary Information:**	
**Corresponding Author's Institution:**	BGI-Shenzhen: BGI Group
**Corresponding Author's Secondary Institution:**	
**First Author:**	Yiyang Chen
**First Author Secondary Information:**	
**Order of Authors:**	Yiyang Chen
	Yunning Lu
	Da Qi
	Yong Bai
	Guixue Hou

Powered by Editorial Manager® and ProduXion Manager® from Aries Systems Corporation

	Siqi Liu
**Order of Authors Secondary Information:**	
**Response to Reviewers:**	Response to Reviewer #1:<br>1.The manuscript reports extensive benchmarking, but the reader may need clearer information on splits, cross-validation design, class balance, etc.<br>Response: I sincerely thank the reviewer for this valuable suggestion, and fully agree that the original manuscript lacked sufficient details regarding the data splitting, cross-validation strategy, and class balance. In the revised manuscript, I have expanded and clarified the experimental description in "Data splitting and model training" section (Lines 561-573) and provided Supplementary Fig. S3 to illustrate the overall workflow. In the section, the revised statements regarding the data splitting and cross validation procedures are as follows "Each dataset was partitioned into a training set and an independent test set at an 8:2 ratio using stratified random splitting. For GONet, hyperparameters were optimized strictly in the 80% training set using 5-fold cross-validation. Based on the optimal hyperparameters determined via cross-validation, the final model was retrained on the entire 80% training set and subsequently evaluated on the held-out 20% independent test set. To assess the stability of GONet, the entire procedure was repeated five times using different random seeds, and the final performance metrics were reported as the average across these five iterations. All comparison methods, regardless of GNN or non-GNN, were evaluated using the same train-test splits to ensure a fair and consistent benchmarking comparison (Supplementary Fig. S3)".<br>For the question of class imbalance, we have summarized the sample sizes, and class distributions for each clinical label in Supplementary Table S1, indicating those datasets with class imbalance. In early version, we did consider impact of class imbalance, the Focal Loss[1] with α and γ parameters was taken to solve such problem. As described in the revised manuscript (Lines 537 to 538), "where p_y represents the predicted probability for true label y, and α and γ are hyperparameters controlling class weighting and focusing strength, respectively".<br><br>Supplementary Figure S3. Illustration of GONet hyperparameters and training strategy.<br>2.All preprocessing steps were performed inside each training fold?<br>Response: I completely agree with you that performing preprocessing steps within the training set is essential in deep learning. As described above and illustrated in Supplementary Figure S3, in each of the five splits, the original dataset was randomly divided into an 80% training set and a 20% independent test set. Subsequently, the learned parameters and selected features of preprocessing steps were determined using only the divided training sets and were applied to the corresponding independent test sets. The independent test set was not accessed during the preprocessing steps, thereby ensuring an unbiased evaluation of model performance.<br>3.The CPTAC pre-processing description states that features with more than 20% missing values were removed and remaining values were imputed with k-nearest neighbors, but the fold-wise handling of these steps is not sufficiently clear.<br>Response: Yes, I agree with your argument and have made the corresponding changes in the revision. All preprocessing procedures were independently performed in the five train-test splits, including missing value filtering, imputation, normalization, and feature selection, which were based on the preprocessing strategies of omics data previously reported[2][3]. Specifically, for the CPTAC datasets, the features with more than 20% missing values were removed, and the remaining missing values were imputed using KNN only based on the training samples. Besides, min-max normalization and ANOVA-based feature selection were also performed only using the training set. The resulting preprocessing parameters and selected features were then applied to the corresponding held-out 20% independent test set.<br>For example, the features selected by ANOVA using the training set were directly adopted in the corresponding test set. The same preprocessing strategy was consistently conducted to all CPTAC omics datasets. I have revised the relevant statements in "Data preprocessing" in Methods section (Lines 411-416) as "data preprocessing procedure followed a step-wise strategy in each random train-test splits, removal of features with more than 20% missing values, imputation of remaining missing values using k-nearest neighbors (KNN), min-max normalization, and ANOVA-based feature selection was performed on the training set. And then processing procedure and selected features were applied to the corresponding independent test sets".

To further clarify the preprocessing procedure, I have provided Supplementary Fig. S4 below to illustrate this workflow.

Supplementary Figure S4. Illustration of data preprocessing.
4.The DGI module appears to be the main contributor, while the autoencoder and gated fusion provide smaller gains in the reported module tests; the manuscript would be stronger if it included an ablation of each component.
Response: Definitely, a comprehensive ablation analysis of each component would strengthen the argument for the contribution of each module in GONet. Following your suggestion, we evaluated GONet performance under different ablated conditions using the same CPTAC datasets with paired mRNA, miRNA, and proteomic profiles for the classification of cancer stages. Specifically, the ablation analysis of GONet was implemented under the following conditions: removal of AE for global sample representation module (noAE), replacement of DGI with GCN or GAT for local molecular representation module, removal of the self-attention mechanism for omics representation module (noOmics), and replacement of the gated fusion mechanism with direct feature concatenation for fusion module (cat). The results derived from such ablation analysis demonstrate that each component contributes to the overall performance of GONet.
As expected, removal of the AE led to decline in classification performance across the seven CPTAC cancer cohorts, replacement of DGI with GCN or GAT resulted in lower predictive performance, deletion of the omics representation module caused a moderate reduction in classification performance, and substitution of the gated fusion module made an average AUROC decrease, respectively (Supplementary Fig. S1 and Supplementary Tables S3-S4). These results indicate that although the modification of DGI provides the largest contribution, the other components capture complementary contributions to the overall performance of GONet.
In the revised manuscript, I have mentioned the description of the ablation analysis of GONet in “Results” section as follows: “To further evaluate the contribution of each architectural component to entire GONet, ablation analyses were adopted on the CPTAC datasets, like removal of AE, replacement of DGI, removal of omics representation and replacement of gated fusion. The ablation results endorsed the conclusions drawn from Figure 2A-D, with DGI playing a role of major contributor. Detailed ablation results are provided in Supplementary Fig. S1A-D and Supplementary Table S3-S4” (Lines 209-215).

Supplementary Figure S1. Module ablation analyses of GONet for cancer stage classification. (A) Ablation of the global sample representation module, (B) local molecular representation module, (C) omics representation module, and (D) feature fusion module.
Supplementary Table4: Quantitative summary of module-wise ablation analyses of GONet across seven CPTAC cancer cohorts for cancer stage classification.
ModelMean AUROCRelative dropInterpretation
Full GONet0.67770Whole GONet
noAE0.613010.55%remove global representations
GCN0.558121.43%replace DGI with GCN
GAT0.589914.87%replace DGI with GAT
noOmics0.63676.43%remove omics representations
cat0.615510.09%replace gated-fusion with concatenation
5.The authors should discuss whether their approach recover cancer-relevant biology.
Response: I agree that biological relevance to the GONet prediction is essential to further strengthen the study significance beyond predictive performance. During manuscript preparation, we initially performed exploratory interpretive analyses of GONet predictions using the original cohort. As this cohort was primarily established for challenging TNM classification and comprised a relatively modest number of samples with diverse biological characteristics, we extended the analysis to a larger proteogenomic dataset with well-defined molecular subtypes, providing a more informative context for interpreting the biological relevance of GONet predictions. During the revision, we thus selected another independent Fu-iCCA cohort with a larger number of subtype cases of intrahepatic cholangiocarcinoma (iCCA), paired mRNA and proteomic data, and functional study to the disease[4]. Upon the Fu-iCCA dataset, GONet achieved a subtype classification performance with an AUROC of 0.98 (Supplementary Fig. S2A). Integrated Gradients (IG) was applied to quantify the contribution of individual molecular features to subtype prediction, yielding higher

Powered by Editorial Manager® and ProduXion Manager® from Aries Systems Corporation

explanation fidelity than the random baseline (0.211 vs. 0.027) and a low attribution error (0.0278). Supplementary Table S7 summarizes the top 100 features with the highest IG attribution scores identified from the global and local modules. The top-ranked features derived from either the global or local modules across the mRNA and protein modalities indicate that GONet captures complementary biological signals across multiple molecular layers rather than relying on a single omics modality (Supplementary Fig. S2B). Furthermore, functional enrichment analysis of these key transcriptomic and proteomic features reveals significant enrichment of biological processes and signaling pathways, which are consistent with previously reported biological characteristics of Fu-iCCA study (Supplementary Fig. S2C). Moreover, these key features are closely associated with subtype-differentially expressed genes or proteins (Supplementary Fig. S2D). Therefore, the interpretation analysis reveals the feasibility of biological relevance mining towards the GONet prediction.
I have given additional statements regarding biological interpretation towards the disease classification based on GONet in "Discussion" section of revised manuscript (Lines 385-391) as "Beyond predictive performance, exploring the relationships between GONet-based disease classification and molecular features may provide additional insights into the biological relevance of the predictions. As pointed out by Sundararajan et al, Integrated Gradients (IG) can be used to quantify the contribution of individual input features to model predictions and facilitate the identification of biologically relevant signals[5]. Further development of such interpretability analyses may provide additional biological insights into GONet-based disease classification".

Supplementary Figure S2. Interpretability analysis of GONet predictions using Integrated Gradients (IG) in an independent Fu-iCCA cohort. (A) Receiver operating characteristic curve demonstrating the subtype classification performance of GONet in the independent Fu-iCCA cohort. GONet achieved robust classification performance with an AUROC of 0.98. (B-C) Distribution of Integrated Gradients (IG) scores for the top 100 features identified from the global and local modules, stratified by molecular modality (transcriptomic and proteomic features). The results demonstrate that highly ranked predictive features were derived from both mRNA and protein modalities. (D) Sankey diagram illustrating the functional associations of the top-ranked mRNA and protein features identified by GONet. The diagram depicts the relationships among molecular features, enriched biological functions, and corresponding Fu-iCCA molecular subtypes, highlighting the biological processes and signaling pathways associated with the identified predictive features. (E) Box plots showing the subtype-specific distribution patterns of the prioritized molecular features. The consistent differential expression patterns across Fu-iCCA subtypes further support the biological relevance of the features identified by GONet.
6.The CPTAC performance remains moderate, and the authors already acknowledge dataset size and omics quality as limiting factors; phrases implying broad generalizability should be softened.
Response: I have accepted your advice to soften the tone regarding GONet performance. I have carefully reviewed and revised statements throughout the manuscript that may overstate the broader generalizability of GONet. Specifically, we have made the following changes:
(1) In the "Introduction" (Lines 131-132), the original statement: "Results from benchmark test and case studies demonstrated that GONet substantially improves predictive performance across diverse cancer datasets" has been corrected to: "Results from benchmark tests and case studies suggest that GONet can improve predictive performance across several cancer datasets".
(2) In "Results" Section 1 (Lines 219-227), the original statement: "As summarized in Fig2E, GONet consistently outperforms all competitors across datasets. On Li's dataset, it achieves an AUROC of 0.9663, representing a 6.39% improvement over Li's method (0.9082). Similar improvements are observed on MOGONET (0.9824 vs. 0.9520) and SynOmics (0.9849 vs. 0.9431), demonstrating strong cross-dataset generalization" has been revised to: "As summarized in Fig2E, GONet was evaluated for cancer classification across the datasets used for the development of these GNN-based methods. On Li's dataset, GONet achieved an AUROC of 0.9663, representing a 6.39% improvement over Li's method (0.9082). On the MOGONET datasets, GONet yielded an AUROC of 0.9824, higher than those of MOGONET (0.9520), MGDMCL (0.9329), and MOGANet (0.9386), respectively. GONet also obtained an AUROC of 0.9849 on the SynOmics dataset, compared with 0.9431 for SynOmics. Together, these results demonstrate the improved prediction of cancer classification performance

by GONet".
(3) In "Results" Section 4 (Lines 330-331), the original statement: "the null hypothesis of equal performance is rejected ($p < 0.001$), confirming the statistically significant superiority of GONet across diverse evaluation scenarios" has been changed to: "the null hypothesis of equal performance was rejected ($p < 0.001$), indicating that GONet achieves statistically difference in the evaluation scenarios".
(4) In the "Discussion" (Lines 401-402), the original statement: "GONet has established a robust and generalizable multi-omics integration framework that provides a methodological foundation for disease prediction" has been restated as: "GONet has established a multi-omics integration framework that potentially provides a methodological foundation for disease classification".

Reference：
[1] Lin, T.-Y., Goyal, P., Girshick, R., He, K. & Dollár, P. Focal Loss for Dense Object Detection. in 2980–2988 (2017).
[2] Wang, T. et al. MOGONET integrates multi-omics data using graph convolutional networks allowing patient classification and biomarker identification. Nat. Commun. 12, 3445 (2021).
[3] Ji, Y., Dutta, P. & Davuluri, R. Deep multi-omics integration by learning correlation-maximizing representation identifies prognostically stratified cancer subtypes. Bioinforma. Adv. 3, vbad075 (2023).
[4] Dong, L. et al. Proteogenomic characterization identifies clinically relevant subgroups of intrahepatic cholangiocarcinoma. Cancer Cell 40, 70–87 (2022).
[5] Sundararajan, M., Taly, A., & Yan, Q. (2017). Axiomatic Attribution for Deep Networks. Proceedings of the 34th International Conference on Machine Learning (ICML), 3319–3328.

Response Reviewer #2:
1.Please explicitly discuss the distinction between the proposed model and existing methods in the literature review paragraphs of the Introduction section.
Response: I sincerely thank you very much for the valuable comments about clearer explanation of the innovation and necessity of our approach compared with previous studies. In response to this comment, we have revised the Introduction section of the new manuscript (Lines 67-121), and the revised text is provided below:
Benefiting from strong capabilities in nonlinear modeling and representation learning, deep learning has become a major methodology for multi-omics integration in cancer analysis16–19. Early deep learning approaches that primarily rely on non-GNN architectures, such as autoencoders (AEs), multilayer perceptrons (MLPs) and attention-based models, transform different omics profiles into unified representations and alleviate problems of high dimensionality and noise. For example, MOCAT is an AE-based framework that integrates mRNA, miRNA, and DNA methylation data across multiple diseases, including Alzheimer's disease and several cancer types, achieving improved classification performance in disease diagnosis, subtype and stage prediction20. Attention-based models have been introduced to enhance multi-omics representation learning by dynamically weighting contributions from different omics features. Methods such as HTML and CLCLSA combine attention mechanisms with contrastive learning strategies and achieve well-improved performance in multi-cancer classification tasks21,22. GNNs, another important class of deep learning models, are designed to capture relational dependencies in graph-structured data through message passing between nodes23–25. Early GNN-based multi-omics approaches are primarily constructed using sample-level similarity graphs for classification tasks, in which nodes represent individual samples and edges encode pairwise relationships derived from omics features. For instance, MOGONET employs graph convolutional networks (GCNs)26 on sample similarity graphs constructed from mRNA, miRNA, and DNA methylation data for prediction of cancer subtypes/stages27. MOGCN constructs sample similarity graphs from CNV, mRNA, and RPPA data in TCGA breast cancer and is applied to subtype classification28. However, those approaches, either traditional non-GNN or sample-similarity-based GNN models, focus on learning global representations at the sample level from the gene expression matrices to integrate omics data. As a result, they cannot utilize the interaction information and are limited in capturing complex nonlinear dependencies across omics features, thereby leaving substantial room for improvement in representation learning for disease and cancer subtype prediction.
Cancer is not solely driven by single-gene aberrations but is regulated by complex molecular interaction networks. If interaction graphs are integrated into GNN-based

multi-omics integration frameworks, with nodes representing gene features and edges characterizing the feature interactions, such a design is expected to enable GNNs to learn biologically local representations from molecular interaction topology and improve disease prediction10,16,29. Recently, SynOmics has extended GCN-based26 architectures to intra- and inter-omic interaction graphs for cancer prognosis prediction30, while Li et al. proposed a hybrid model to improve prediction performance for breast cancer subtype classification32, in which MLPs encode global omics profiles and GCN or GAT31 models molecular interaction networks. Nevertheless, there remains large room for improvement in current GNN-based models. First, feature extraction is insufficient across multiple levels. MLP-based encoders tend to compress high-dimensional omics data toward task-specific representations, potentially losing informative variations. Standard message-passing in GNNs is largely confined to local neighborhoods, and may therefore have limited ability to capture broader dependencies. In addition, most current methods assume homogeneous node representations, whereas they ignore intrinsic heterogeneity across omics types such as mRNA and proteins. Therefore, a novel feature extractor is required to be capable of representing both omics characteristics and interaction patterns. Second, representation fusion remains insufficiently coordinated. Since simple concatenation may lead to unstable hierarchical representations and weak discrimination power in downstream tasks, a new fusion mechanism that can resolve conflicts and inconsistencies among multiple representations is highly desirable. Third, with the growing proteomic evidence for cancer classification, a promising avenue is to introduce proteomics and the corresponding interaction networks into GNN-based models to enhance discriminative capacity33–36.

2.Most of the baseline methods compared in this paper were published very early. The authors are suggested to compare with the methods in recent years.

Response: I agree that comparisons with recently developed multi-omics integration methods would provide a more comprehensive evaluation of GONet. To address this concern, we conducted an extensive survey of recent publications on multi-omics integration approaches from 2025 to 2026, and found that GNN-based approaches have become increasingly prevalent in this field; however, these approaches are fundamentally different from our model, such as molecular interactions and proteomics. Therefore, we have expanded the comparison study of GONet by including two additional recent GNN-based methods, MGDMCL (2025), and MOGANet (2026), as well as a new non-GNN-based method, MOAEAM (2026). Following the dataset-based comparison strategy used in the previous version of our manuscript, GONet was evaluated on the datasets used for the development of these methods. Briefly, the AUROC values obtained by MGDMCL, MOGANet, and MoAEAM are 0.9329, 0.9386, and 0.9305, respectively, whereas GONet achieves an AUROC of 0.9824 on the same dataset, which is higher than those obtained by the three new methods. In addition, the CPTAC datasets were also used for the performance evaluation in these methods, and the comparisons indicate that GONet also achieves competitive performance on the CPTAC datasets as well. Overall, these additional comparisons further support the effectiveness of GONet when evaluated against recently developed multi-omics integration methods across both their benchmark datasets and the CPTAC datasets used for evaluation.

In the revised manuscription, I have made the corresponding changes in the “Results” Section 2 as follows “(Lines 186-189) GNN-based methods have been applied to cancer type and stage prediction using multi-omics data in recent five years, including approaches with or without explicit modeling of interaction information, such as MOGONET27, MOGCN28, SynOmics30, and Li’s method31, MGDMCL40, and MOGANet41”, “(Lines 219-227) As summarized in Fig2E, GONet was evaluated for cancer classification across the datasets used for the development of these GNN-based methods. On Li’s dataset, GONet achieved an AUROC of 0.9663, representing a 6.39% improvement over Li’s method (0.9082). On the MOGONET datasets, GONet yielded an AUROC of 0.9824, higher than those of MOGONET (0.9520), MGDMCL (0.9329), and MOGANet (0.9386), respectively. GONet also obtained an AUROC of 0.9849 on the SynOmics dataset, compared with 0.9431 for SynOmics. Together, these results demonstrate the improved prediction of cancer classification performance by GONet”, and “(Lines 232-236) GONet consistently yielded higher prediction performance than other GNN-based methods for cancer stage classification across the CPTAC datasets, with an average AUROC of 0.6420, corresponding to 16.41% relative improvement over Li’s method, 39.78% over MOGONET, 29.15% over MOGCN, 16.20% over SynOmics, 21.54% over MOGANet (0.5282), and 31.75% over MGDMCL

(0.4873)". Meanwhile, Figure 2E has been updated to include the newly added comparison results as indicated below.

Also, I have made the restatements regarding non-GNN-based methods in Results Section 4 in the revision as follows "(lines290-293) A wide range of non-GNN-based multi-omics integration methods have been applied to cancer type and stage classification, including several conventional methods, such as RF43, SVM44, LR45, AE, MLP, and recently published approaches, such as HTML22, CLCLSA21, and MOAEAM48", "(lines 308-313) the different approaches exhibited variable prediction performance on the remaining 8 datasets (Fig 4C). GONet consistently showed higher prediction performance than the comparison methods, with an average AUROC of 0.9495 versus 0.8687 for the non-GNN methods. Notably, the GNN-based comparison methods showed lower prediction performance on the same datasets, with an average AUROC of 0.7465", "(lines 316-318) All the non-GNN methods were applied to the CPTAC datasets for assessment of cancer stage classification. As shown in Fig. 4D, these methods achieve AUROC values ranging from 0.5080 to 0.6226", and "(lines 324-327) As shown in Fig 4E, non-GNN-based approaches exhibited lower predictive performance, with average AUROC values spanning 0.4780 to 0.6012 ($p<0.05$). Moreover, proteomics integration led to inconsistent gains across methods and across the 7 cancer types (Fig 4F)". Also, the previous Figure 4 has also been revised in the revision as below.

3.The manuscript did not provide details on hyperparameter selections. Please include a sensitivity analysis versus key hyperparameters to ensure a fair comparison.
Response: I am sorry that we did not provide sufficient details on the hyperparameter selection in the original version, although relevant information was provided in the Supplementary Materials. Following your advice, we have performed a sensitivity analysis of the key hyperparameters during the revision of manuscript.
In the revised manuscript, in "Hyperparameters Optimization" of "Methods" section, I have given the detailed description of hyperparameter selection as follows: "The hyperparameters of GONet were optimized on the training set using Optuna66, a framework based on Bayesian optimization principles. Specifically, the optimization was conducted within a predefined search space through many independent trials. In each trial, Optuna proposed a candidate hyperparameter configuration based on the results of previous trials, which was then evaluated using 5-fold cross-validation. The mean AUROC across the five folds was used as the performance metric to guide selection of the next candidate configuration of hyperparameters. After completing the optimization process, the hyperparameters configuration yielding the highest mean AUROC was selected as the final configuration for GONet (see Table S5-S6)" (Lines 575-583).
To implement the sensitivity analysis of the key hyperparameters, we selected three key hyperparameter, including learning rate, AE depth, and GNN depth, and tested the GONet performance by varying one hyperparameter while remaining the other hyperparameters no change across CPTAC datasets.
For learning rate, we evaluated the GONet performance under five learning rate settings, 1e-5, 3e-5, 1e-4, 3e-4, and 1e-3. As shown in Figure 1A below, GONet showed relatively stable performance across learning rates ranging from 3e-5 to 3e-4 in different datasets, indicating that GONet is robust within a reasonable range of learning rates. For AE depth, we assessed the GONet performance under different AE depths ranging from 2 to 5 layers. As presented in Figure 1B below, the GONet performance varied with AE depth settings in different datasets, with shallow networks (2-3 layers) performing better for LUAD and HNSCC, whereas deeper layers provided better performance in some other cohorts. These results suggest that optimal AE depth of GONet should be selected on different multi-omics datasets. For GNN depth, we evaluated GNN depths ranging from 2 to 5 layers. As shown in Figure 1C below, the optimal performance of GONet was mainly achieved with 2 or 3 layers, suggesting that GNN with deeper layers is not always beneficial for the GONet performance.
Taken together, the sensitivity analysis indicates that GONet performance varies with different parameter settings across datasets, while no single hyperparameter setting consistently dominates across all cohorts. These results further suggest dataset-specific hyperparameter optimization rather than fixed hyperparameters. Detailed hyperparameter search spaces and optimal values from Optuna are provided in Supplementary Table S5-S6.

Powered by Editorial Manager® and ProduXion Manager® from Aries Systems Corporation

Figure 1. Sensitivity analysis of GONet to key hyperparameters across seven cancer cohorts. (A) Learning rate, (B) autoencoder depth, and (C) GNN depth. In each analysis, one hyperparameter was varied while all other hyperparameters were fixed at the corresponding optimized configuration. Each point represents the mean AUROC obtained from five-fold cross-validation.
4.The tick marks and labels on the axes should be enlarged to improve readability for the readers.
Response: Yes, I have thoroughly revised Figures 2-4 by enlarging the font sizes of the axis labels, tick marks, and legends to improve readability. These figures have been updated accordingly in the revised manuscript.
5.What are the configurations of the simulation computer used for running these models? This hardware information can be added to the manuscript.
Response: Following your suggestion, I have added the configuration information of hardware and software to "Data splitting and model training" section of the revised manuscript (Lines 571-573) as follows: "All experiments were performed on a Linux system equipped with an NVIDIA A100 GPU (80 GB VRAM). The GONet were implemented in Python 3.8, using PyTorch 2.0 with CUDA 11.8, and PyTorch Geometric (PyG) 2.4.0".

Reviewer #3:
1.First, although Deep Graph Infomax (DGI) alleviates the over-smoothing issue of GCN/GAT through mutual information maximization, DGI is still essentially a local contrastive learning method with limited capability to capture long-range dependencies. The hierarchical regulatory relationships within omics (e.g., transcription factor-mRNA-protein cascade regulation) were not considered.
Response: I agree that DGI is fundamentally a local contrastive learning framework and has limited capacity to capture long-range dependencies. On the other hand, DGI can maximize mutual information between local node representations and an entire graph representation, encouraging the aggregation of nodes beyond neighborhoods to a certain extent[1], yet the encoder itself remains local. In our study, hierarchical regulatory relationships (e.g., transcription-factor-miRNA-target cascades) are directly encoded in the different multi-omics graph of GONet, which comprises mRNA, miRNA and protein nodes connected by both intra-omics and inter-omics edges, while DGI learns representations on the heterogeneous graph structure. I would address the reviewer's concern at two complementary angles, whether cross-omics information contributes to predictive performance, and whether biologically hierarchical interactions contribute to the model's predictions.
First, the contribution of cross-omics information is supported by both graph statistics and predictive performance. As shown in Fig. 1A, the integrated tri-omics networks exhibit higher average node degrees (approximately 112-144 across the CPTAC cohorts) than the corresponding single-omic networks, providing more opportunities for multi-hierarchical information flow across molecular layers. Moreover, under the same DGI framework, the integrated mRNA-miRNA-protein model achieved improved predictive performance compared with individual single-omic models for stage classification across different cancer cohorts on CPTAC datasets (Results section 3). Particularly, the integrated model achieved an average AUROC of 0.68, compared with 0.64 (protein-only), 0.57 (miRNA-only) and 0.60 (mRNA-only). These results support the utility of cross-omics information for prediction performance.
Second, regarding whether hierarchical relationships contribute to the predictions, we applied Integrated Gradients (IG) to quantify the importance of individual edges[2]. Using CPTAC-LUAD as a representative cohort, we calculated an IG attribution score for each edge and retained the top 25% of edges ranked by their absolute attribution scores (47,714 edges). As illustrated in Fig. 1B, these highly attributed edges contain both within-omics interactions and cross-omics relationships. Importantly, some biologically supported hierarchical regulatory relationships were also identified among high attribution edges, including not only direct molecular interactions but also hierarchical cross-omic regulatory dependencies. For example, as illustrated in Fig. 1C, CDH1-EGFR represents a direct protein-level interaction[3], whereas SRF → miR-199a-5p → CDH1 forms a TF-miRNA-target cascade including transcriptional and post-transcriptional regulation[4]. Similarly, miR-9/AGO2-mediated regulation of ACOT7 represents a post-transcriptional regulatory relationship[5], while SRF-ACOT7 is supported by TF-binding evidence[6]. Additional high-attribution interactions previously reported in the literature (EGFR-miR-200a[7], miR-21-CDH1[8], SRF-MCL1[9]) further illustrate that specific hierarchical cross-omics interactions contribute to the model's

predictions.

Figure 1. Cross-omics network connectivity and biologically relevant interactions captured by GONet. (A) Degree distribution of integrated tri-omics and single-omic networks across CPTAC cancer cohorts. (B) The network graph of highly attributed molecular relationships identified by Integrated Gradients (IG). (C) Representative physical interactions and hierarchical regulatory relationships among highly attributed interactions.

Table 1: Literature evidence supporting highly attributed molecular interactions identified by Integrated Gradients.

No.RelationshipsPMIDCancer/modelMain finding
1EGFR - CDH135074920epithelial cellsheteroreceptor complex
2EGFR - miR-2119597153Lung cancerEGFR positively regulates miR-21
3EGFR - miR-200a29552158NSCLCEGFR/ERK/miR-200a signaling
4miR-21 - CDH130568467NSCLCmiR-21 increases SMAD7, reduces E-cadherin
5miR-9/AGO2 - ACOT728472401Human cellsmiR-9/WIG1/AGO2-dependent ACOT7 silencing
6EGFR - AGO223636329Cancer cellEGFR phosphorylates AGO2-Y393
7SRF - miR-199a- CDH125080937Cross-cancer evidenceSRF transactivates miR-199a, miR-199a-5p targets E-cadherin
8SRF - MCL19880563HematopoieticSRF/Elk-1 transcriptionally regulates MCL1
9SRF – ACOT727426982TFBSTFBS-supported
10ACOT7 - phenotype36139682LUAD/PC9ACOT7 knockdown suppresses proliferation/migration
11MCL1 - phenotype31200834LUADMCL1 supports survival
12CDH1- phenotype32015681LUADPromotes self-renewal and stemness in lung cancer stem-like cells
13miR-21 - phenotype29663730/34858053LUADmiR-21 promotes EMT/invasion; high miR-21 predicts poor prognosis
14miR-199a - phenotype33868472/35844793NSCLCmiR-199a suppresses tumor phenotypes

2.Second, proteomics was evaluated only in seven cancer types from CPTAC with relatively small sample sizes, leading to moderate absolute AUROC values. Although an attention mechanism was employed, no biological interpretation was provided for the learned attention weights (e.g., which omics are more important in specific cancer types, which molecular interactions are most critical for classification).

Response: During the revision, I have followed your suggestion and performed an attention-based interpretability analysis to answer which omics are more important in specific cancer types, and which molecular interactions are most critical for classification.

To quantify the attention-based importance of each omics layer during information aggregation, an omics-level attention contribution score C_j based on the learned attention weight matrix A was calculated as follows:

$$C_j=\frac{1}{M}\sum_{i=1}^{M}A_{ij}$$

where A_{ij} denotes the attention weight directed from omics j to omics i, and M represents the total number of omics modalities (mRNA, miRNA, and protein). Thus, C_j represents the average attention weight assigned to omics j during cross-omics information aggregation[10,11] (Wang et al., 2024; Cai et al., 2024). Based on the C_j values across different cancer datasets, proteomics receives higher attention scores in BRCA and HNSCC, miRNA in LSCC, CCRCC, LUAD, and COAD, and mRNA in PDAC, respectively, suggesting that the relative attention contribution to different omics in GONet varies across cancer types (Figure 2A). This observation is consistent with previous studies reporting that the predictive contribution of individual omics modalities varies considerably across cancer types[12].

To further explore the cross-omics interactions learned by GONet, the attention weights A_{ij} are used to quantify the relative attention assigned to interactions between omics i and omics j. As shown in Figure 2B-H, the distribution of attention weights also varies across cancer types. The interactions involving miRNA are prominent in LUAD, LSCC, COAD and CCRCC, mRNA-related interactions in PDAC, and protein-related interactions in BRCA and HNSCC. There is no single interaction with consistently high attention contribution scores across these datasets. However, the attention contribution of individual omics is consistent with that of their corresponding interactions. For instance, C_j is the highest values for proteomics in BRCA and HNSCC, and the A_{ij} values for proteomics-related interactions are also

Powered by Editorial Manager® and ProduXion Manager® from Aries Systems Corporation

relatively high in these two datasets.

Figure2. Multi-omics importance weights and critical molecular interaction networks across cancer types for classification. (A). heatmap of omics-specific importance weights (mRNA, protein, miRNA) for seven cancer types (BRCA, LSCC, CCRCC, PDAC, LUAD, HNSCC, COAD), highlighting which omics contribute most to classification in each cancer. (B-H) multi-omics interaction networks showing the critical molecular interactions (edge weights) among mRNA, miRNA, and protein for each cancer type (PDAC, LUAD, LSCC, HNSCC, COAD, CCRCC, BRCA).

3.Third, the method heavily relies on curated interaction networks from public databases (e.g., BioGRID, STRING). Experiments were mainly conducted on common cancer types (e.g., BRCA, LUAD), and the applicability to rare cancers or small-sample scenarios remains unclear. No direct association was established with clinical indicators (e.g., survival prognosis, treatment response).

Response: I fully acknowledge that GONet still has several limitations, especially regarding its applicability to rare cancers, small-sample scenarios, and in clinical interpretation.

Regarding the issues of the rare cancers and small-sample scenarios, the applicability and performance of GONet in these settings remain to be determined. To develop and evaluate GONet, we do need the basic prerequisites, such as sufficient multi-omics data for integration, enough large sample size for model training, and established methods for multi-omics data integration for cancer classification. At present, publicly available studies and datasets for rare cancers are often difficult to match these prerequisites. Therefore, our study has focused primarily on common cancer types for which sufficient cancer studies and datasets are available to meet these prerequisites. I fully agree that application of GONet to rare cancer and small-sample scenarios is an important direction for future model development.

Regarding the clinical relevance, the solid cancer datasets used in this study are derived from TCGA and CPTAC with well-defined clinically annotations, which provided the foundation for developing and evaluating GONet for cancer classification. Since GONet can effectively recognize cancer types and stages, which are clinically relevant characteristics, its predictions are partially related to clinical indicators. However, I have to acknowledge that the current version of GONet does not provide the clinical interpretation of the prediction results. This limitation may provide an important direction for the future development of GONet, particularly through the introduction of integrated gradients (IG) to identify important predictive features and explore their associations with clinical outcomes, such as survival, treatment response, and drug sensitivity.

Reference:

[1]Veličković P, Fedus W, Hamilton W L, et al. Deep Graph Infomax[A]. arXiv, 2018.
[2]Sundararajan M, Taly A, Yan Q. Axiomatic Attribution for Deep Networks[C]//International conference on machine learning. 3319-3328.
[3]Sullivan B, Light T, Vu V, et al. Mechanical disruption of E-cadherin complexes with epidermal growth factor receptor actuates growth factor-dependent signaling[J]. Proceedings of the National Academy of Sciences of the United States of America, 2022, 119(4): e2100679119.
[4]Zhao X, He L, Li T, et al. SRF expedites metastasis and modulates the epithelial to mesenchymal transition by regulating miR-199a-5p expression in human gastric cancer[J]. Cell Death and Differentiation, 2014, 21(12): 1900-1913.
[5]Lee H C, Jung S H, Hwang H J, et al. WIG1 is crucial for AGO2-mediated ACOT7 mRNA silencing via miRNA-dependent and -independent mechanisms[J]. Nucleic Acids Research, 2017, 45(11): 6894-6910.
[6]Plaisier C L, O’Brien S, Bernard B, et al. Causal Mechanistic Regulatory Network for Glioblastoma Deciphered Using Systems Genetics Network Analysis[J]. Cell Systems, 2016, 3(2): 172-186.
[7]Zhou P, Hu J, Wang X, et al. Epidermal growth factor receptor expression affects proliferation and apoptosis in non-small cell lung cancer cells via the extracellular signal-regulated kinase/microRNA 200a signaling pathway[J]. Oncology Letters, 2018, 15(4): 5201-5207.
[8]Li X, Wu X. MiR-21-5p promotes the progression of non-small-cell lung cancer by regulating the expression of SMAD7[J]. OncoTargets and Therapy, 2018, 11: 8445-8454.
[9]Townsend K J, Zhou P, Qian L, et al. Regulation of MCL1 through a serum response

Powered by Editorial Manager® and ProduXion Manager® from Aries Systems Corporation

	factor/Elk-1-mediated mechanism links expression of a viability-promoting member of the BCL2 family to the induction of hematopoietic cell differentiation[J]. The Journal of Biological Chemistry, 1999, 274(3): 1801-1813.<br>[10]Cai J, Wang R, Zhao D, et al. AuD-Former: A Hierarchical Transformer Network for Multimodal Audio-Based Disease Prediction[J]. arXiv preprint arXiv:2410.09289<br>[11]Wang Y, He J, Wang D, et al. Multimodal transformer with adaptive modality weighting for multimodal sentiment analysis[J]. Neurocomputing, 2024, 572: 127181.<br>[12]Su X, Hu P, Li D, et al. Interpretable identification of cancer genes across biological networks via transformer-powered graph representation learning[J]. Nature Biomedical Engineering, 2025, 9(3): 371-389.
**Additional Information:**	
**Question**	**Response**
Are you submitting this manuscript to a special series or article collection?	No
**Experimental design and statistics**<br><br>Full details of the experimental design and statistical methods used should be given in the Methods section, as detailed in our Minimum Standards Reporting Checklist. Information essential to interpreting the data presented should be made available in the figure legends.<br><br>Have you included all the information requested in your manuscript?	Yes
**Resources**<br><br>A description of all resources used, including antibodies, cell lines, animals and software tools, with enough information to allow them to be uniquely identified, should be included in the Methods section. Authors are strongly encouraged to cite Research Resource Identifiers (RRIDs) for antibodies, model organisms and tools, where possible.<br><br>Have you included the information requested as detailed in our Minimum Standards Reporting Checklist?	Yes
**Availability of data and materials**<br><br>All datasets and code on which the	Yes

conclusions of the paper rely must be either included in your submission or deposited in publicly available repositories (where available and ethically appropriate), referencing such data using a unique identifier in the references and in the “Availability of Data and Materials” section of your manuscript.<br><br>Have you have met the above requirement as detailed in our Minimum Standards Reporting Checklist?	
GigaScience has policies and guidelines in place for the use of generative AI-writing tools such as ChatGPT. If you have used such writing tools to assist with writing the manuscript this must be declared and cited in the text. Authors should not list AI-writing tools and other AI-assisted technologies as an author or co-author and should acknowledge that they are fully responsible for text generated or refined by AI-writing tools.<p><br><br>A summary of use (particularly in the introduction or among methods) needs to be included at the end of the paper, and the outputs should also be included as a supplementary file hosted in GigaDB or other open repositories. Please read our guidelines for more information. <p><br><br>By submitting to GigaScience, you are aware of the journal's AI-writing tools policy, and if you have declared use of such tools below, you have acknowledged this where appropriate in your manuscript and have made a summary of use and outputs available. <p><br><b>AI-assisted writing tools have been used in the preparation of this manuscript?	No

Manuscript
Click here to access/download;Manuscript;GIGA-D-26-00172.revised.updated-20261013(1).docx

GONet: A Molecular Interaction-Guided Graph Learning Framework for Multi-Omics Cancer Classification

Yiyang Chen (陈毅洋)[1,2], Yunning Lu (陆运凝)[2,3], Da Qi (戚达)[2], Yong Bai (白勇)[4,*], Guixue Hou (侯桂雪)[2,*], Siqi Liu (刘斯奇)[1,2,*]

chenyiyang@genomics.cn; luyunning@bgi.cn; mr_daqi@hotmail.com; Yong.bai@hotmail.com; houguixue@bgi.com; siqiliu@genomics.cn

1 College of Life Sciences, University of Chinese Academy of Sciences, Beijing 100049, China

2 BGI-Shenzhen, Shenzhen, Guangdong 518083, China

3 School of Biology and Biological Engineering, South China University of Technology, Guangzhou, 510641, China

4 BGI Research, Shenzhen, 518083, China

*Corresponding authors: siqiliu@genomics.cn; houguixue@bgi.com; Yong.bai@hotmail.com.

ORCID:

Yiyang Chen: https://orcid.org/0009-0003-3791-4797

Yunning Lu: https://orcid.org/0009-0008-6705-7235

Da Qi: https://orcid.org/0000-0001-6079-9764

Yong Bai: https://orcid.org/0000-0001-5960-8000

Guixue Hou: https://orcid.org/0000-0003-0342-9593

Siqi Liu: https://orcid.org/0000-0001-9744-3681

Abstract

Integrating multi-omics profiles with molecular interaction networks enables biologically informed representation learning by combining molecular measurements with prior biological knowledge. Although graph neural networks (GNNs) are well suited for modeling molecular interaction networks, existing multi-omics methods

often rely on shallow representation learning or incomplete fusion strategies, which fail to fully capture complementary information and complex dependencies across heterogeneous omics layers, resulting in suboptimal performance and limited generalizability in cancer classification tasks.

Here, we propose GONet, a hierarchical GNN framework that integrates omics expression profiles, including mRNA, miRNA, and protein data, with molecular interaction networks, where nodes represent omics features and edges represent molecular interactions. GONet learns global omics representations via an autoencoder, captures local structural dependencies using Deep Graph Infomax (DGI), and enhances omics-specific representations through an attention mechanism. A gated fusion module further enables adaptive integration of heterogeneous features, improving cancer subtype and stage classification.

GONet was systematically evaluated on 13 publicly available benchmark datasets used in prior studies, covering 10 cancer types, ensuring fair comparison across methods. Across mRNA and miRNA datasets, GONet achieved an average AUROC of 0.9262, outperforming representative GNN- and non-GNN-based methods by 13.77% and 6.35%, respectively. On an independent CPTAC cohort spanning seven cancer types, it improved performance by 25.22% and 13.91%, respectively. Incorporating proteomics further improved cancer stage classification by 5.56%, demonstrating complementary predictive value. Ablation studies confirmed the effectiveness of the DGI-based module. Overall, GONet provides a unified framework for biologically informed multi-omics graph representation learning and cancer classification.

Keywords: multi-omics data integration, graph neural network, cancer classification, molecular interaction network

Introduction

Cancer remains one of the leading causes of morbidity and mortality worldwide[1], driven by highly complex and heterogeneous molecular mechanisms. Elucidating these mechanisms is essential for understanding cancer initiation and progression, as well as for improving clinical diagnosis, treatment, and prevention[2,3]. The advent of high-throughput omics technologies has enabled systematic characterization of cancer across multiple molecular layers[4,5]. Genomics reveals the landscape of genetic alterations underlying cancer development[4,6], while transcriptomics and proteomics provide gene expression patterns, including gene expression dynamics, post-translational modifications, and molecular interactions[4,7,8]. In essence, cancer initiation and development are cumulative results of genetic mutations[9]; therefore, the pronounced heterogeneity of cancers is likely associated with the interactions across networks involving genome, transcriptome, and proteome[4,10]. Although single-omics data provide rich experimental evidences and bioinformatic insights at the molecular level, from the perspective of the central dogma[11], interactions across different molecular levels during the transmission of genetic information also influence biological phenotypes[12,13]. Consequently, integrating multi-omics information is essential for a comprehensive characterization of cancer biology[5,14,15]. With continuous accumulation of large-scale resources, such as The Cancer Genome Atlas (TCGA)[3], and the Clinical Proteomic Tumor Analysis Consortium (CPTAC)[2], researchers have increasingly focus on multi-omics integration studies.

Benefiting from strong capabilities in nonlinear modeling and representation learning, deep learning has become a major methodology for multi-omics integration in cancer analysis[16–19]. Early deep learning approaches that primarily rely on non-GNN architectures, such as autoencoders (AEs), multilayer perceptrons (MLPs) and attention-based models, transform different omics profiles into unified representations and alleviate problems of high dimensionality and noise. For example, MOCAT is an AE-based framework that integrates mRNA, miRNA, and DNA methylation data across multiple diseases, including Alzheimer’s disease and several cancer types, achieving improved classification performance in disease diagnosis, subtype and stage prediction[20]. Attention-based models have been introduced to enhance multi-omics representation learning by dynamically weighting contributions from different omics features. Methods such as HTML and CLCLSA combine attention mechanisms with contrastive learning strategies and achieve well-improved performance in multi-cancer classification tasks[21,22]. GNNs, another important class of deep learning models, are

designed to capture relational dependencies in graph-structured data through message passing between nodes[23–25]. Early GNN-based multi-omics approaches are primarily constructed using sample-level similarity graphs for classification tasks, in which nodes represent individual samples and edges encode pairwise relationships derived from omics features. For instance, MOGONET employs graph convolutional networks (GCNs)[26] on sample similarity graphs constructed from mRNA, miRNA, and DNA methylation data for prediction of cancer subtypes/stages[27]. MOGCN constructs sample similarity graphs from CNV, mRNA, and RPPA data in TCGA breast cancer and was applied to subtype classification[28]. However, those approaches, either traditional non-GNN or sample-similarity-based GNN models, focus on learning global representations at the sample level from the gene expression matrices to integrate omics data. As a result, they cannot utilize the interaction information and are limited in capturing complex nonlinear dependencies across omics features, thereby leaving substantial room for improvement in representation learning for disease and cancer subtypes prediction.

Cancer is not solely driven by single-gene aberrations but is regulated by complex molecular interaction networks. If interaction graphs are integrated into GNN-based multi-omics integration frameworks, with nodes representing gene features and edges characterizing the feature interactions, such a design is expected to enable GNNs to learn biologically local representations from molecular interaction topology and improve disease prediction[10,16,29]. Recently, SynOmics has extended GCN-based[26] architectures to intra- and inter-omic interaction graphs for cancer prognosis prediction[30], while Li et al. proposed a hybrid model to improve prediction performance for breast cancer subtype classification[31], in which MLPs encode global omics profiles and GCN or GAT[32] models molecular interaction networks. Nevertheless, there remains large room for improvement in current GNN-based models. First, feature extraction is insufficient across multiple levels. MLP-based encoders tend to compress high-dimensional omics data toward task-specific representations, potentially losing informative variations. Standard message-passing in GNNs is largely confined to local neighborhoods, and may therefore have limited ability to capture broader dependencies. In addition, most current methods assume homogeneous node representations, whereas they ignore intrinsic heterogeneity across omics types such as mRNA and proteins. Therefore, a novel feature extractor is required to be capable of representing both omics characteristics and interaction patterns. Second, representation fusion remains insufficiently coordinated. Since simple concatenation may lead to

unstable hierarchical representations and weak discrimination power in downstream tasks, a new fusion mechanism that can resolve conflicts and inconsistencies among multiple representations is highly desirable. Third, with the growing proteomic evidence for cancer classification, a promising avenue is to introduce proteomics and the corresponding interaction networks into GNN-based models to enhance discriminative capacity[33–36].

In this study, the GONet is proposed, a novel multi-level GNN framework for cancer classification by integration of multi-omics profiles, mRNAs, miRNAs and proteins, with interaction networks. GONet employs autoencoders to capture global representations, and applies Deep Graph Infomax (DGI)[37] to model local representations from molecular interaction networks. Considering the heterogeneous contribution across omics, GONet introduces an attention mechanism to generate omics representations that highlight of differential regulation of gene expression. Furthermore, a gated fusion module is designed in GONet to dynamically reconcile global, local, and omics-specific representations, thereby alleviating potential conflicts and improving integration consistency. Results from benchmark tests and case studies suggest that GONet can improve predictive performance across several cancer datasets.

Results

GONet: A hierarchical graph neural network for multi-omics and molecular interaction integration

GONet is a modular, end-to-end framework that synergistically integrates matrix-based multi-omics data (e.g., transcriptomics and proteomics profiles) with graph-structured biomolecular interaction networks to derive biologically meaningful, low-dimensional representations for downstream classification tasks, including cancer staging and subtyping. Accordingly, the framework comprises two major parts: 1) dual-input: preprocessed multi-omics matrices and a curated interaction graph upon transcriptomes and proteomics; 2) model architecture: multi-level feature extraction followed by adaptive fusion; and downstream prediction classification. A schematic overview of framework is presented in Fig. 1.

In the dual-input part, multi-omics matrices, including mRNA, miRNA, and proteomics profiles, are preprocessed into a unified matrix $X \in R^{n \times d}$, where n and d represent the number of samples and features, respectively. For each sample, the personalized molecular interaction graph $G = (V, E, H)$ is constructed based on the curated interactions from public databases, in which V as nodes represents molecules, E as

edges denotes interactions, and H stands for sample-specific molecular quantities (Methods).

In the model architecture part, three core modules are employed, feature extraction, feature fusion, and prediction. In feature extraction modules: three synergistic sub-modules are designed to generate complementary data representations. A) Global sample learning: regarding the widespread application of autoencoders for dimensionality reduction in multi-omics data integration and addressing the limitation of MLP-based compression in capturing information-rich global representations, AE is applied to compress the input matrix X into a low-dimensional representation H_{global}, while the decoder reconstructs the input $\hat{X}$ by minimizing mean squared error (MSE) loss. B) Local molecular learning: considering global representations overlook local patterns in molecular interaction networks, and that conventional GCN and GAT models suffer from over-smoothing and limited discriminative capacity, DGI is applied to maximize node-graph mutual information via the contrastive loss, generating local representations H_{local} from the molecular interaction graph G. C) Local omics learning: addressing the lack of omics-specific information, a self-attention mechanism[38] (SAM) at omics-level adaptively weights inter-omics dependencies by learnable coefficients into H_{omics}. In feature fusion modules, an adaptive gated fusion mechanism[39] (GFM) aligns the complementary representations (H_{global}, H_{local}, and H_{omics}) from the three sub-modules. The GFM adaptively weights and combines the three representations to generate a unified representation, H_{final}, allowing the model to adjust the relative contributions of the individual modules. In prediction modules, H_{final} is passed through to a fully-connected classifier, which outputs class probabilities for cancer subtype or stage prediction (Fig. 1).

Fig 1. Overview of the GONet framework. GONet integrates multi-omics profiles (mRNA, miRNA, protein) with biomolecular interaction networks for cancer classification. **Data collection and graph construction**. Multi-omics datasets and curated molecular interaction networks are used to construct sample-specific molecular interaction graphs. **Model architecture.** Multi omics matrices serve as inputs to the global learning module (AE-based), while the sample-specific molecular interaction graphs are processed by the local learning module (DGI-based). In parallel, the local omics module captures inter-omics dependencies using attention mechanism (SAM-based). The resulting representations are fused via feature fusion module (GFM-based) and passed to a prediction module for the downstream classification tasks.

Performance evaluation of GONet modules compared with previous GNN-based methods

GNN-based methods have been applied to cancer type and stage prediction using multi-omics data in recent five years, including approaches with or without explicit modeling of interaction information, such as MOGONET[27], MOGCN[28], SynOmics[30], and Li's method[31], MGDMCL[40], and MOGANet[41]. As shown in Fig 2A, MOGONET and MOGCN exhibit inconsistent performance across datasets, often performing best on their original benchmark datasets. In contrast, SynOmics and Li's method demonstrate relatively more stable cross-dataset performance, suggesting that interaction information may be beneficial for effective cancer classification. However, SynOmics primarily considered the contribution of molecular interactions while underutilizing sample-level information, whereas Li's method integrates both local molecular interactions and global sample-level representations, yielding robust and generalizable multi-omics integration. Despite these advances, their representation learning and feature fusion strategies still leave room for further improvement.

To further analyze the contribution of each component, we compare GONet with Li et al.'s framework, which employs MLP-based global learning, GCN/GAT-based local learning, and concatenation-based fusion. In contrast, GONet adopts AE-based global encoding, DGI-based local learning, SAM-based omics modeling, and GFM-based fusion. Using the BRCA dataset from Li et al. (Li_dataset), all modules were evaluated independently under a unified classifier. For global learning, AE and MLP achieve comparable performance (AUROC: 0.9631±0.0047 vs. 0.9619±0.0108; Fig. 2B). For local learning, DGI significantly outperforms GCN and GAT (0.8558±0.0491 vs. 0.7536±0.0193 and 0.7483±0.0154; Fig. 2C). The SA-based omics module achieves an AUROC of 0.8077±0.039. For feature fusion, GFM slightly improves over concatenation (0.9663±0.0106 vs. 0.9578±0.0067; Fig. 2D). To further evaluate the contribution of each architectural component to entire GONet, ablation analyses were adopted on the CPTAC datasets, like removal of AE, replacement of DGI, removal of omics representation and replacement of gated fusion. The ablation results endorsed the conclusions drawn from Fig. 2A-D, with DGI playing a role of major contributor. Detailed ablation results are provided in Supplementary Fig. S1A-D and Supplementary Table S3-S4. Overall, each module of GONet positively contributes to subtype classification, especially in local learning.

Performance of the full GONet framework was further evaluated for cancer subtype and stage classification in comparison with representative GNN-based methods on their

original datasets using mRNA and miRNA data. As summarized in Fig2E, GONet was evaluated for cancer classification across the datasets used for the development of these GNN-based methods. On Li's dataset, GONet achieved an AUROC of 0.9663, representing a 6.39% improvement over Li's method (0.9082). On the MOGONET datasets, GONet yielded an AUROC of 0.9824, higher than those of MOGONET (0.9520), MGDMCL (0.9329), and MOGANet (0.9386), respectively. GONet also obtained an AUROC of 0.9849 on the SynOmics dataset, compared with 0.9431 for SynOmics. Together, these results demonstrate the improved prediction of cancer classification performance by GONet.

Next question is whether the prediction performance of GONet and the other GNN-based methods is comparable on the same omics datasets. CPTAC dataset was used for such evaluation, using mRNA and miRNA data across seven cancer types (BRCA, CCRCC, COAD, HNSCC, LUAD, LSCC, and PDAC). As shown in Fig 2E and Table S4, GONet consistently yielded higher prediction performance than other GNN-based methods for cancer stage classification across the CPTAC datasets, with an average AUROC of 0.6420, corresponding to 16.41% relative improvement over Li's method, 39.78% over MOGONET, 29.15% over MOGCN, 16.20% over SynOmics, 21.54% over MOGANet (0.5282), and 31.75% over MGDMCL (0.4873) ($p<0.05$). Although performance gains are consistent, absolute AUROC values remain moderate, suggesting that dataset size and omics quality remain key limiting factors for multi-omics cancer prediction.

Fig 2. Module-level comparison and performance evaluation of GONet against existing GNN-based models. **A)** Comparative predictive performance of representative GNN-based methods across multiple public multi-omics cancer datasets. **B-D,** Module-specific evaluation of key architectural components in GONet on the BRCA subtype classification task using paired mRNA and miRNA data. **B)** *Global learning module.* Performance comparison of AE in GONet with a MLP baseline. **C)** *Local learning module.* Performance comparison of DGI with GCN and GAT baselines. **D)** *Feature fusion module.* Comparison between the adaptive GFM used in GONet and CAT. **E)** Generalization performance of GONet compared with representative GNN-based methods on original benchmark datasets (left) and CPTAC datasets (right).

Proteomics contributes to GONet for cancer classification

Unlike mRNA and miRNA data, which primarily capture transcriptional information, proteomics data directly reflects the functional state of gene expression[33,35,42].

Besides, large-scale resources, such as BioGRID[43] and STRING[44], accumulate extensive protein-protein interaction (PPI) information, providing inherent support for GNN-based modeling. These characteristics motivate the evaluation of proteomics as an alternative omics modality within the GONet for cancer classification prediction.

To systematically assess the applicability of GONet to proteomics data, a modality substitution experiment was conducted, where transcriptomic inputs (miRNA and mRNA) were replaced with proteomics data together with corresponding PPI networks across seven CPTAC cancer types.

As presented Fig 3A, GONet well accommodated the proteomics data, achieving performance comparable to transcriptomics-based models (average AUROC: 0.6360 vs. 0.6420). At the individual cancer-type level, the proteomics-based predictions achieved slightly higher AUROC values than transcriptomics-based predictions in five of seven cancer types (BRCA, CCRCC, LUAD, HNSCC and PDAC), whereas modestly lower performance was observed in COAD and LSCC. Overall, these results suggest that the proteomics data is a viable modality for GNN-based cancer stage prediction with GONet, at least in the CPTAC cohort.

An arising question concerns the relative contribution of individual omics to cancer stage prediction in GONet. As summarized in Fig 3B, proteomics alone yielded higher predictive performance among the three single-omics inputs, with an average AUROC of 0.6360, compared to 0.6038 for mRNA and 0.5721 for miRNA. At the individual dataset, proteomics-based predictions outperformed those derived from either mRNA or miRNA alone in five of the seven CPTAC cancer types. This suggests that proteomics appears a stronger discriminative signals for cancer staging within the GONet framework.

Finally, we evaluated whether the integration of three omics data could further improve cancer stage prediction in CPTAC. As shown in Fig 3B, the mean AUROC for tri-omics integration of miRNA, mRNA and proteomics in GONet was 0.6777, which is obviously higher than that derived by proteomics alone (0.6360; $p<0.05$) or transcriptomics (miRNA-mRNA:0.6420; $p<0.05$). These results demonstrate that multi-omics integration provides complementary information, with proteomics contributing essential functional-level signals that enhance cancer stage prediction.

Fig 3. Evaluation of proteomics integration in GONet. A) Comparison of transcriptomics-based (mRNA–miRNA) and proteomics-based inputs for cancer stage

classification across CPTAC cohort. **B)** Performance of single-, dual- and tri-omics integration (mRNA, miRNA, protein), summarized by AUROC across CPTAC datasets.

Performance comparison of GONet and non-GNN methods for cancer classification

A wide range of non-GNN-based multi-omics integration methods have been applied to cancer type and stage classification, including several conventional methods, such as RF[45], SVM[46], LR[47], AE, MLP, and recently published approaches, such as HTML[22], CLCLSA[21], and MOAEAM[48]. Given the strong performance of GONet compared with existing GNN-based methods, a systematic evaluation was conducted from three perspectives: i) comparison with existing non-GNN methods on their original datasets and on CPTAC, ii) evaluation under proteomics integration, and iii) global statistical comparison across all datasets and methods.

A total of 13 datasets were recruited, including those previously studied by HTML (COAD, ESCA, GBMLGG, SARC, STAD, STES, THCA, UCEC), CLCLSA (LGG, KIPAN), MOGONET, SynOmics, and Li et al. (Table S1). All datasets contained the mRNA and miRNA profiles with cancer stage or subtype labels. Fourteen approaches, including GNN- and non-GNN-based methods, were applied onto each dataset, with results summarized in Supplementary Data (Table S1). The performance can be categorized into 3 circumstances, 1) due to unclear reason, all approaches achieved relatively poor performance on the 2 datasets HTML_GBMLGG and HTML_STAD, with AUROC ranging from 0.6297 to 0.7272 (Fig 4A); 2) most approaches obtained near-saturated predictions on 3 datasets HTML_ESCA, HTML_STES, and HTML_KIPAN, with AUROC values exceeding 0.99 (Fig 4B); and 3) the different approaches exhibited variable prediction performance on the remaining 8 datasets (Fig 4C). GONet consistently showed higher prediction performance than the comparison methods, with an average AUROC of 0.9495 versus 0.8687 for the non-GNN methods. Notably, the GNN-based comparison methods showed lower prediction performance on the same datasets, with an average AUROC of 0.7465. These results strengthen our argument that improvements in representation learning and interaction modeling are critical for GNN-based multi-omics methods.

All the non-GNN methods were applied to the CPTAC datasets for assessment of cancer stage classification. As shown in Fig. 4D, these methods achieve AUROC values ranging from 0.5080 to 0.6226, all significantly lower than GONet (0.6420; $p < 0.05$). Although overall performance remains moderate across all methods, likely due to

limited sample size and task difficulty, GONet consistently achieves the best performance among both GNN and non-GNN approaches.

Giving the importance of proteomics, a question is raised if non-GNN-based approaches can effectively utilize tri-omics integration (mRNA, miRNA, and proteomics) on CPTAC. As shown in Fig 4E, non-GNN-based approaches exhibited lower predictive performance, with average AUROC values spanning 0.4780 to 0.6012 ($p<0.05$). Moreover, proteomics integration led to inconsistent gains across methods and across the 7 cancer types (Fig 4F).

Finally, a Friedman test across 27 experimental scenarios was conducted to statistically evaluate the advantages of GONet. As shown in Supplementary Fig. S5, GONet ranked first, and the null hypothesis of equal performance was rejected ($p < 0.001$), indicating that GONet achieves statistically differences in the evaluation scenarios.

Fig 4. Systematic benchmarking of GONet performance across multi-omics cancer classification tasks. A-C) Performance comparison for bi-omics integration (mRNA-miRNA) between GONet and 14 competing methods across 13 datasets collected from previous studies grouped by task difficulty. **D)** Heatmap depicting AUROC values for bi-omics integration (mRNA-miRNA) between GONet and the 14 competing methods across seven CPTAC cancers. **E)** Heatmap showing AUROC values for tri-omics integration (mRNA, miRNA, and proteomics) between GONet and the 14 competing methods across seven CPTAC cancers. **F)** Heatmap illustrating the absolute AUROC improvements (ΔAUROC) for each method after integrating proteomics data.

Discussion

GNNs have recently emerged as a powerful approach for multi-omics data integration by incorporating molecular interaction networks, as exemplified by the Li et al. and the SynOmics framework[30,31]. However, as shown in Fig. 2A, systematic comparison of the current GNN-based approaches reveals that integration of interactions does not consistently yield improve performance, suggesting that effectiveness depends not only on the introduction of interaction information, but also on how efficiently they are leveraged. In complex cancer omics networks, conventional GCN and GAT models inherently behave as low-pass filters[49,50], aggregating local neighborhoods information, and readily suffering from over-smoothing during propagation processes, resulting in indistinguishable node representations and reduced discriminability[50,51]. To avoid the drawback, GONet innovatively incorporates DGI, which extracts more discriminative node representations from molecular interaction networks by

maximizing mutual information between node-level representations and graph-level representations[37]. From an information-theoretic perspective, mutual information maximization captures global context and higher-order dependencies, and has been shown to improve embedding quality for downstream tasks[52,53]. Furthermore, DGI's contrastive learning framework distinguishes real node-graph pairs from corrupted ones, encouraging robustness to noise and improving representation quality[54,55]. Module comparison experiments confirm that the DGI markedly improves the standard GCN or GAT, yielding superior classification performance (Fig. 2C). The improvement is consistent with prior reports in biological network representation learning, in which DGI enhances representation quality and downstream task performance in molecular-disease association networks[56], protein-protein interaction networks[57], and single-cell transcriptome-derived gene regulatory networks[58].

Proteins represent functional executors of biological processes, whereas transcripts primarily reflect upstream regulatory activity[59]. Accordingly, proteomics provides complementary functional and interaction-level signals, yet remains underutilized in existing approaches. Some studies integrated only protein expression data (MOGCN with RPPA profiles[28]), while others included protein interaction networks without protein abundance, leaving synergistic modeling unexplored[31]. In this study, we simultaneously integrated protein abundance and associated interaction networks from the CPTAC cohort. Proteomes alone exhibited predictive power comparable to mRNA and miRNA, while integration with transcriptomic data further improves performance (Fig. 3A, B and Fig. 4C). Ablation analysis shows that neither abundance-only nor interaction-only signals yield strong performance (AUROC=0.6039 and 0.5587), whereas their combination yields a substantial gain (AUROC=0.6777; Fig. S7). These results indicate that predictive improvements arise from the synergistic integration of proteomic abundance and interaction structure. Notably, although incorporation of proteome into GONet improves performance, the gain magnitude remains moderate. GONet still leaves substantial room for further improvement, primarily in two directions: more advanced model architectures, such as large language model-based approaches[60,61], and larger cohort sizes to enhance deep learning capacity and better capture mRNA and protein characteristics[62].

Beyond predictive performance, exploring the relationships between GONet-based disease classification and molecular features may provide additional insights into the biological relevance of the predictions. As pointed out by Sundararajan et al, Integrated Gradients (IG) can be used to quantify the contribution of individual input features to

model predictions and facilitate the identification of biologically relevant signals[63]. Further development of such interpretability analyses may provide additional biological insights into GONet-based disease classification.

In summary, a modular GNN-based framework for integration of omics data with molecular interactions, GONet, was proposed and examined its capacity in cancer types and stages classification. GONet adopts a hierarchical representation learning strategy, AE-based global encoding, DGI-based local interaction modeling, attention-based modality weighting, and gated multi-level fusion. Extensive evaluations across multiple cancer cohorts and omics settings demonstrate that GONet achieves consistently robust performance compared with both GNN- and non-GNN-based methods. Particularly, GONet enables systematic joint modeling of proteomic abundance and interaction topology, with stable performance gains when proteomics data are involved. Overall, GONet has established a multi-omics integration framework that potentially provides a methodological foundation for disease classification.

Methods

Data preprocessing

The benchmark datasets followed the original formats in previous literatures, whereas the CPTAC data were processed using the procedures described by Wang et al. and Ji et al.[27,65]. Specifically, data preprocessing procedure followed a step-wise strategy in each random train-test splits, removal of features with more than 20% missing values, imputation of remaining missing values using k-nearest neighbors (KNN), min-max normalization, and ANOVA-based feature selection was performed on the training set. And then processing procedure and selected features were applied to the corresponding independent test sets.

Molecular interaction networks

Comprehensive molecular interaction networks in this study were curated by aggregation of public interaction repositories, including PPIs derived from BioGRID[43], STRING[44], and IID[66], mRNA-related regulatory interactions sourced from RISE[67], NPInter[68] and RNAInter[69], miRNA-related regulatory interactions retrieved from miRNet[70]. All interaction data were limited to human interactions, and nodes and edges involved such interactions were further filtered to remove incomplete or erroneous records. Molecular identifiers were standardized using UniProt IDs for proteins, HGNC symbols for mRNAs, and miRDB IDs for miRNAs.

Graph construction based on multi-omics interactions

For each patient sample s, a personalized molecular interaction graph G_s was constructed by integrating multi-omics profiles with curated interaction networks and defined as:

$$G_s = (V_s, E_s, H_s)$$

where V_s represents nodes with molecular entities (mRNAs, miRNAs, and proteins) detected in sample s, with v_i and v_j correspond to two nodes of V_s. E_s denotes edges derived from the curated interaction networks for sample s. Each edge $e_{ij} \in E_s$ between molecules v_i and v_j is defined as:

$$e_{ij} = \begin{cases} 1, & if \text{ an } interaction \text{ exists } between\, v_i \text{ and } v_j \\ 0, & otherwise \end{cases}$$

H_s corresponds to the normalized expression values of the respective molecular entities in sample s.

Construction of GONet

Global sample learning module

The AE used for global sample learning comprises an encoder and decoder.

Encoder processing. Let the original multi-omics feature matrix $X \in R^{n \times m}$ serve as input, where n represents the number of samples and m denotes the total number of molecular entities. A low dimensional global representation, $H_{global} \in R^{n \times d_g} (d_g \ll m)$, is compressed by encoder functioned $f_{enc}(\cdot)$:

$$H_{global} = f_{enc}(X)$$

where the f_{enc} consists of fully connected layers followed by LeakyReLU activations to capture non-linear patterns.

Decoder processing. The reconstructed representation $\hat{X}$ is generated from H_{global} by decoder functioned $f_{dec}(\cdot)$ to ensure that the compressed H_{global} retains the essential information of X. The reconstruction process is formulated as:

$$\hat{X} = f_{dec}\left(H_{global}\right)$$

where $\hat{X} \in R^{n \times m}$ represents the approximation of original matrix X. The decoder maintains a symmetric architecture with the encoder.

Reconstruction loss. The AE parameters are optimized by minimizing reconstruction loss L_{rec} between X and $\hat{X}$, defined as:

$$L_{\mathrm{rec}} = \| X - \hat{X} \|_2^2$$

where $\| \ \|_2^2$ is defined as the mean squared error (MSE).

Local molecular learning module

DGI model for local molecular learning is applied to learn high-quality local representations at two hierarchical scales: the node-level (individual molecules) and the graph-level **(**the entire molecular interaction network**)**.

Node-level representation. For each sample s, node-level representations $H_{node} \in R^{|V_s| \times d_l}$ are learned via graph convolutional encoder $GCN(\cdot)$ that aggregates information from neighboring molecules in the sample-specific molecular interaction network G_s:

$$H_{node} = GCN(G_s)$$

where $|V_s|$ represents the number of molecule nodes in sample s, and d_l denotes the dimensionality of node representation. Each row $h_v \in H_{node}$ corresponds to a d_l-dimensional embedding of molecule v.

Graph-level representation. The graph-level representation $H_{local} \in R^{2d_l}$ is obtained by aggregating H_{node} with a dual-pooling strategy (mean and max pooling) to capture the global topology of the molecular network:

$$H_{local} = \left[\frac{1}{|V_s|} \sum_{v \in V_s} v h_v \right] \oplus \left[\max_{v \in V_s} h_v \right]$$

where $\oplus$ denotes vector concatenations, and the left and right part indicate pooling strategies to generate $2d_l$-dimensional vector for sample s.

Maximization of Mutual Information. To ensure that node-level representations encode global topology information, a discriminator D is introduced to maximize the mutual information between H_{node} and H_{local}, which distinguishes between real node-graph pairs (h_v, H_{local}) from the true interaction graph G_s, and corrupted pairs $(\tilde{h}_v, H_{local})$, as described below:

$$L_{DGI} = -\frac{1}{|V_s|}\sum_{v \in V_s}\left[logD(h_v, H_{local}) + log\left(1 - D\left(\widetilde{h_v}, H_{local}\right)\right)\right]$$

where $\tilde{h}_v$ represents the corrupted node representation obtained from a corrupted graph with randomly shuffle nodes.

Local omics learning module

The local omics learning module aggregates node-level representations into omics-specific representations and integrates information from different omics through an adaptive attention mechanism.

Omics-specific representation aggregation. To reduce noise and capture omics-specific signatures, omics-specific representations H_m for each omics modality m are generated by averaging the node-level representations of its constituent molecules V_m:

$$H_m = \frac{1}{|V_m|}\sum_{v \in V_m} h_v$$

where V_m represents the set of nodes belonging to modality m. The representations of all omics modalities are then stacked as:

$$H_{stack} = \begin{bmatrix} H_1 \\ H_2 \\ \vdots \\ H_{|M|} \end{bmatrix}, \qquad H_{stack} \in R^{|M| \times d_l}$$

where $|M|$ is the number of omics.

Self-attention mechanism. To adaptively weight the contributions of different omics modalities, the attention weights A are computed as follows:

$$A = softmax\left(\frac{\sigma(H_{stack}W_Q)(H_{stack}W_k)^{\top}}{\sqrt{d_k}}\right)$$

where W_Q and W_K are learnable weight matrices and d_k is the attention dimension. The fused omics-level representation H_{omics} is obtained by weighting H_{stack} with A:

$$H_{omics} = A(H_{stack}W_V)$$

Contrastive loss. To optimize the omics module and ensure that the learned representations H_{omics} are discriminative, a supervised contrastive loss L_{sup} is minimized to encourage sample with same label y to have similar embeddings:

$$L_{sup} = SupConLoss(H_{omics}, y; \tau)$$

where τ represents the temperature hyperparameter controlling the scale of the loss.

Feature fusion module

The feature fusion module integrates global, local, and omics representations through representation alignment followed by adaptive fusion to synthesize multi-level information.

Representation alignment. Given the different feature spaces and dimensions of H_{global} H_{local} and H_{omics}, the three representations are projected into aligned representations Z_{mod} with a common dimension d_f by a fully-connected layer followed by a LeakyReLU activation:

$$Z_{mod} = LeakyReLU(W_{mod}H_{mod} + b_{mod}), mod \in \{global, local, omics\}$$

where W_{mod} and b_{mod} are learnable parameters.

Representation fusion. An adaptive gated mechanism $f_{\text{Gate}}(\cdot)$ is employed to learn relative importance weights α_{mod} of each representation:

$$\alpha_{mod} = [\alpha_{global}, \alpha_{local}, \alpha_{omics}] = f_{\text{Gate}}([\mathrm{Z}_{global} \oplus \mathrm{Z}_{local} \oplus \mathrm{Z}_{omics}])$$

$$\sum_{mod} \alpha_{mod} = 1$$

where $\oplus$ denotes concatenation, and the gating function is implemented as a softmax operation to normalize the importance weights across the three modules. The final fused representation H_{final} is defined as the convex combination of the Z_{mod}:

$$H_{final} = \sum_{mod} \alpha_{mod} Z_{mod}, \qquad H_{\text{final}} \in R^{n \times d_f}.$$

Prediction module

The prediction module performs cancer stage or subtype classification based on H_{final}, and the prediction performance is assessed using focal loss[71].

Classification prediction. The fused representation H_{final} is fed into a fully-connected layer with learnable parameters W_c and b_c, followed by a $softmax$ function to predict cancer stages or subtypes. The predicted probability vector p is computed as:

$$p = softmax\left(W_c H_{final} + b_c\right)$$

Focal loss. Focal loss is used as the classification loss L_{cls}, which measures discrepancy between the predicted probability p and the true class $y \in \{1, \ldots, C\}$:

$$L_{cls} = -\alpha\left(1 - p_y\right)^{\gamma} log\left(p_y\right)$$

where p_y represents the predicted probability for true label y, and α and γ are hyperparameter controlling class weighting and focusing strength, respectively.

Total loss function

The GONet framework is trained by minimizing a joint multi-objective loss function:

$$L_{total} = \lambda_{rec}L_{rec} + \lambda_{DGI}L_{DGI} + \lambda_{sup}L_{sup} + \lambda_{cls}L_{cls} + \lambda_{reg}L_{reg}$$

where L_{reg} is a regularization item to prevent overfitting, and trade-off hyperparameters $\{\lambda_{\mathrm{rec}}, \lambda_{\mathrm{DGI}}, \lambda_{\mathrm{contra}}, \lambda_{\mathrm{cls}}, and\ \ \lambda_{\mathrm{reg}}\}$ control the relative contribution of each loss items.

Data splitting and model training

Each dataset was partitioned into a training set and an independent test set at an 8:2 ratio using stratified random splitting. For GONet, hyperparameters were optimized strictly in the 80% training set using 5-fold cross-validation. Based on the optimal hyperparameters determined via cross-validation, the final model was retrained on the entire 80% training set and subsequently evaluated on the held-out 20% independent test set. To assess the stability of GONet, the entire procedure was repeated five times using different random seeds, and the final performance metrics were reported as the average across these five iterations. All comparison methods, regardless of GNN or non-GNN, were evaluated using the same train-test splits to ensure a fair and consistent benchmarking comparison (Supplementary Fig. S3). All experiments were performed on a Linux system equipped with an NVIDIA A100 GPU (80 GB VRAM). The GONet were implemented in Python 3.8, using PyTorch 2.0 with CUDA 11.8, and PyTorch Geometric (PyG) 2.4.0.

Hyperparameter Optimization

The hyperparameters of GONet were optimized on the training set using Optuna[72], a framework based on Bayesian optimization principles. Specifically, the optimization was conducted within a predefined search space through many independent trials. In each trial, Optuna proposed a candidate hyperparameter configuration based on the results of previous trials, which was then evaluated using 5-fold cross-validation. The mean AUROC across the five folds was used as the performance metric to guide selection of the next candidate configuration of hyperparameters. After completing the optimization process, the hyperparameters configuration yielding the highest mean AUROC was selected as the final configuration for GONet (see Table S5-S6).

Abbreviations

The following table describes the meaning of various abbreviations and acronyms used throughout the thesis.

Abbreviation	Meaning
AE	AutoEncoder
AUROC	Area Under the Receiver Operating Characteristic curve
CPTAC	Clinical Proteomic Tumor Analysis Consortium
DGI	Deep Graph Infomax
GAT	Graph Attention Network
GCN	Graph Convolutional Network
GFM	Gated Fusion Mechanism
GNN	Graph Neural Network
IG	Integrated Gradients
MLP	Multi-layer perceptron
MSE	Mean Squared Error
PPI	Protein–Protein Interaction
RF	Random Forest
SAM	Self-Attention Mechanism
SVM	Support Vector Machine
TCGA	The Cancer Genome Atlas

Acknowledgments

Thanks for the support of the High-performance Computing Platform of YaZhou Bay Science and Technology City Advanced Computing Center (YZBSTCACC).

Funding

The work was supported by the National Key Research and Development Program of China (grant no.: 2023YFC3402900).

Data Availability

All datasets used in this study are publicly available from their respective original sources, including CLCLSA, HTML, MOGONET, SynOmics, and Li's dataset [21,22,27,30,31]. The CPTAC datasets were obtained from the LinkOmicsKB database [64]. Detailed information on the data sources is provided in Supplementary Table S1.

Availability of Source Code and Requirements

Project name: GONet

Project home page: https://github.com/EayonYang/GONet.git

License: Apache-2.0 license

Operating system(s): Ubuntu 22.04

Programming language: Python 3.8 or higher

Other requirements: PyTorch 2.1 or higher, Pytorch Geometric 2.4 or higher, and other dependencies specified in the project repository

RRID: SCR_028640

Conflict of interest

Some of the authors are affiliated with BGI, the sponsor of GigaScience. All editorial handling and peer review of this manuscript were conducted independently by the journal's editorial team in accordance with the journal's standard editorial policies and procedures, to ensure transparency, objectivity, and impartiality. The authors have no additional competing interests to declare.

Reference

1. Bray F, Laversanne M, Sung H, Ferlay J, Siegel RL, Soerjomataram I, et al. Global cancer statistics 2022: GLOBOCAN estimates of incidence and mortality worldwide for 36 cancers in 185 countries. *CA Cancer J Clin*. 2024, 74(3):229-263; doi: 10.3322/caac.21834.

2. Li Y, Dou Y, Da Veiga Leprevost F, Geffen Y, Calinawan AP, Aguet F, et al. Proteogenomic data and resources for pan-cancer analysis. *Cancer Cell*. 2023, 41(8):1397-1406; doi: 10.1016/j.ccell.2023.06.009.

3. Aaltonen LA, Abascal F, Abeshouse A, Aburatani H, Adams DJ, Agrawal N, et al. Pan-cancer analysis of whole genomes. *Nature*. 2020, 578(7793):82-93; doi: 10.1038/s41586-020-1969-6.

4. Manzoni C, Kia DA, Vandrovcova J, Hardy J, Wood NW, Lewis PA, et al. Genome, transcriptome and proteome: the rise of omics data and their integration in biomedical sciences. *Brief Bioinform*. 2018, 19(2):286-302; doi: 10.1093/bib/bbw114.

5. Chakraborty S, Sharma G, Karmakar S, Banerjee S. Multi-OMICS approaches in cancer biology: New era in cancer therapy. *Biochim Biophys Acta Mol Basis Dis*. 2024, 1870(5):167120; doi: 10.1016/j.bbadis.2024.167120.

6. Huang C-Y, Huang W-K, Yeh K-Y, Chang JW-C, Lin Y-C, Chou W-C. Integrating comprehensive genomic profiling in the management of oncology patients: Applications and challenges in Taiwan. *Biomed J*. 2025, 48(5):100851; doi: 10.1016/j.bj.2025.100851.

7. Bose A, Datta S, Mandal R, Ray U, Dhar R. Increased heterogeneity in expression of genes associated with cancer progression and drug resistance. *Transl Oncol*. 2024, 41:101879; doi: 10.1016/j.tranon.2024.101879.

8. Ferlier T, Coulouarn C. Regulation of Gene Expression in Cancer—An Overview. *Cells*. 2022, 11(24):4058; doi: 10.3390/cells11244058.

9. Hanahan D. Hallmarks of Cancer: New Dimensions. Cancer Discov. 2022, 12(1):31-46; doi: 10.1158/2159-8290.CD-21-1059.

10. Li W, Ballard J, Zhao Y, Long Q. Knowledge-guided learning methods for integrative analysis of multi-omics data. *Comput Struct Biotechnol J*. 2024, 30:23:1945-1950; doi: 10.1016/j.csbj.2024.04.053.

11. Crick FHC. Central Dogma of Molecular Biology. *Nature*. 1970, 227(5258):561-3; doi: 10.1038/227561a0.

12. Pan Y, Lei X, Zhang Y. Association predictions of genomics, proteinomics, transcriptomics, microbiome, metabolomics, pathomics, radiomics, drug, symptoms,

environment factor, and disease networks: A comprehensive approach. *Med Res Rev*. 2022, 42(1):441-461; doi: 10.1002/med.21847.

13. Panditrao G, Bhowmick R, Meena C, Sarkar RR. Emerging landscape of molecular interaction networks: Opportunities, challenges and prospects. *J Biosci*. 2022, 47(2):24; doi: 10.1007/s12038-022-00253-y.

14. Luo Y, Zhao C, Chen F. Multiomics Research: Principles and Challenges in Integrated Analysis. *BioDesign Res*. 2024, 6:0059; doi: 10.34133/bdr.0059.

15. Subramanian I, Verma S, Kumar S, Jere A, Anamika K. Multi-omics Data Integration, Interpretation, and Its Application. *Bioinforma Biol Insights*. 2020, 14:1177932219899051; doi: 10.1177/1177932219899051.

16. Thapa K, Kinali M, Pei S, Luna A, Babur Ö. Strategies to include prior knowledge in omics analysis with deep neural networks. *Patterns*. 2025, 6:101203; doi: 10.1016/j.patter.2025.101203.

17. Liu J, Cen X, Yi C, Wang F, Ding J, Cheng J, et al. Challenges in AI-driven Biomedical Multimodal Data Fusion and Analysis. *Genomics Proteomics Bioinformatics*. 2025, 23(1):qzaf011; doi: 10.1093/gpbjnl/qzaf011.

18. Ballard JL, Wang Z, Li W, Shen L, Long Q. Deep learning-based approaches for multi-omics data integration and analysis. *BioData Min*. 2024, 17(1):38; doi: 10.1186/s13040-024-00391-z.

19. Cai Z, Poulos RC, Liu J, Zhong Q. Machine learning for multi-omics data integration in cancer. *iScience*. 2022, 25(2):103798; doi: 10.1016/j.isci.2022.103798.

20. Yao X, Jiang X, Luo H, Liang H, Ye X, Wei Y, et al. MOCAT: multi-omics integration with auxiliary classifiers enhanced autoencoder. *BioData Min*. 2024, 17:9; doi: 10.1186/s13040-024-00360-6.

21. Zhao C, Liu A, Zhang X, Cao X, Ding Z, Sha Q, et al. CLCLSA: Cross-omics linked embedding with contrastive learning and self attention for integration with incomplete multi-omics data. *Comput Biol Med*. 2024, 170:108058; doi: 10.1016/j.compbiomed.2024.108058.

22. Lu Y, Peng R, Dong L, Xia K, Wu R, Xu S, et al. Multiomics dynamic learning enables personalized diagnosis and prognosis for pancancer and cancer subtypes. *Brief Bioinform*. 2023, 24(6):bbad378; doi: 10.1093/bib/bbad378.

23. Valous NA, Popp F, Zörnig I, Jäger D, Charoentong P. Graph machine learning for integrated multi-omics analysis. *Br J Cancer*. 2024, 131:205 – 211; doi: 10.1038/s41416-024-02706-7.

24. Khoshraftar S, An A. A Survey on Graph Representation Learning Methods. *ACM Trans Intell Syst Technol*. 2024,15(1):1–55 ; doi: 10.1145/3633518.

25. Khemani B, Patil S, Kotecha K, Tanwar S. A review of graph neural networks: concepts, architectures, techniques, challenges, datasets, applications, and future directions. *J Big Data*. 2024, 11:18; doi: 10.1186/s40537-023-00876-4.

26. Kipf TN, Welling M. Semi-Supervised Classification with Graph Convolutional Networks. *International Conference on Learning Representations (ICLR)*; 2017. https://openreview.net/forum?id=SJU4ayYgl

27. Wang T, Shao W, Huang Z, Tang H, Zhang J, Ding Z, et al. MOGONET integrates multi-omics data using graph convolutional networks allowing patient classification and biomarker identification. *Nat Commun*. 2021, 12(1):3445; doi: 10.1038/s41467-021-23774-w.

28. Li X, Ma J, Leng L, Han M, Li M, He F, et al. MoGCN: A Multi-Omics Integration Method Based on Graph Convolutional Network for Cancer Subtype Analysis. *Front Genet*. 2022, 13:806842; doi: 10.3389/fgene.2022.806842.

29. Xiao S, Lin H, Wang C, Wang S, Rajapakse JC. Graph Neural Networks With Multiple Prior Knowledge for Multi-Omics Data Analysis. *IEEE J Biomed Health Inform*. 2023, 27(9):4591-4600; doi: 10.1109/JBHI.2023.3284794.

30. Alif MN, Zhang W. SynOmics: integrating multi-omics data through feature interaction networks. *Brief Bioinform*. 2025, 26(6):bbaf595; doi: 10.1093/bib/bbaf595.

31. Li B, Nabavi S. A multimodal graph neural network framework for cancer molecular subtype classification. *BMC Bioinformatics*. 2024, 25(1):27; doi: 10.1186/s12859-023-05622-4.

32. Veličković P, Cucurull G, Casanova A, Romero A, Liò P, Bengio Y. Graph Attention Networks. *International Conference on Learning Representations (ICLR)*; 2018. https://openreview.net/forum?id=rJXMpikCZ

33. Kustatscher G, Collins T, Gingras AC, Guo T, Hermjakob H, Ideker T, et al. Understudied proteins: opportunities and challenges for functional proteomics. *Nature Methods*. 2022,19(7):774-779 ; doi: 10.1038/s41592-022-01454-x.

34. Dong L, Lu D, Chen R, Lin Y, Zhu H, Zhang Z, et al. Proteogenomic characterization identifies clinically relevant subgroups of intrahepatic cholangiocarcinoma. *Cancer Cell*. 2022, 40(1):70-87.e15; doi: 10.1016/j.ccell.2021.12.006.

35. Mun D-G, Bhin J, Kim S, Kim H, Jung JH, Jung Y, et al. Proteogenomic Characterization of Human Early-Onset Gastric Cancer. *Cancer Cell*. 2019, 35(1):111-124.e10; doi: 10.1016/j.ccell.2018.12.003.

36. Gao Q, Zhu H, Dong L, Shi W, Chen R, Song Z, et al. Integrated Proteogenomic Characterization of HBV-Related Hepatocellular Carcinoma. *Cell*. 2019, 179(2):561-577.e22; doi: 10.1016/j.cell.2019.08.052.

37. Veličković P, Fedus W, Hamilton WL, Liò P, Bengio Y, Hjelm RD. Deep Graph Infomax. *International Conference on Learning Representations (ICLR)*; 2019. https://openreview.net/forum?id=rklz9iAcKQ

38. Vaswani A, Shazeer N, Parmar N, Uszkoreit J, Jones L, Gomez AN, et al. Attention Is All You Need. *Advances in Neural Information Processing Systems*. 2017; https://proceedings.neurips.cc/paper/2017/hash/3f5ee243547dee91fbd053c1c4a845aa-Abstract.html

39. Arevalo J, Solorio T, Montes-y-Gómez M, González FA. Gated multimodal networks. Neural Comput Appl. 2020, 32:10209–10228; doi: 10.1007/s00521-019-04559-1.

40. Chen W, Qiu H. MGDMCL: A multi-omics integration framework based on masked graph dynamic learning and multi-granularity feature contrastive learning for biomedical classification. *Comput Methods Programs Biomed*. 2025, 271:109024; doi: 10.1016/j.cmpb.2025.109024.

41. Wu H, Wu H, Xin X, Liu J. MOGANet: A Multi-omics Graph Attention Network for Cancer Diagnosis and Biomarker Identification. *Interdiscip Sci Comput Life Sci*. 2026; doi: 10.1007/s12539-026-00828-5.

42. Shuken SR. An Introduction to Mass Spectrometry-Based Proteomics. *J Proteome Res*. 2023, 22(7):2151-2171; doi: 10.1021/acs.jproteome.2c00838.

43. Oughtred R, Rust J, Chang C, Breitkreutz B, Stark C, Willems A, et al. The BioGRID database: A comprehensive biomedical resource of curated protein, genetic, and chemical interactions. *Protein Sci*. 2021, 30(1):187–200; doi: 10.1002/pro.3978.

44. Szklarczyk D, Kirsch R, Koutrouli M, Nastou K, Mehryary F, Hachilif R, et al. The STRING database in 2023: protein–protein association networks and functional enrichment analyses for any sequenced genome of interest. *Nucleic acids research*. 2023, 51(D1):D638–D646; doi: 10.1093/nar/gkac1000.

45. Breiman L. Random Forests. *Mach Learn*. 2001, 45(1):5 – 32; doi: 10.1023/A:1010933404324.

46. Suykens JAK, Vandewalle J. Least Squares Support Vector Machine Classifiers. *Neural Process Lett*. 1999, 9(3):293–300; doi: 10.1023/A:1018628609742.

47. Buckley J, James I. Linear regression with censored data. *Biometrika*. 1979, 66(3):429–436; doi: 10.1093/biomet/66.3.429.

48. Zhao S, Sun J, Yin Y, Yi M. MOAEAM: Multi-Omics Data Integration With Autoencoder and Attention Mechanisms for Cancer Patient Classification and Biomarker Identification. *IEEE J Biomed Health Inform*. 2026, 30(5):3991–4001; doi: 10.1109/JBHI.2025.3628490.

49. Wu Z, Pan S, Long G, Jiang J, Zhang C. Beyond Low-Pass Filtering: Graph Convolutional Networks With Automatic Filtering. *IEEE Trans Knowl Data Eng*. 2023, 35(7):6687–6697; doi: 10.1109/TKDE.2022.3186016.

50. Li Q, Han Z, Wu X-M. Deeper Insights into Graph Convolutional Networks for Semi-Supervised Learning. *Proc AAAI Conf Artif Intell*. 2018, 32(1); doi: 10.1609/aaai.v32i1.11604.

51. Wu Y, Hu X, Fan X, Ma W, Gao Q. Learning Data-Driven Propagation Mechanism for Graph Neural Network. *Electronics*. 2023,12(1):46 ; doi: 10.3390/electronics12010046.

52. Sun F-Y, Hoffmann J, Verma V, Tang J. InfoGraph: Unsupervised and Semi-supervised Graph-Level Representation Learning via Mutual Information Maximization. *International Conference on Learning Representations (ICLR)*; 2020. https://openreview.net/forum?id=r1lfF2NYvH.

53. Hjelm RD, Fedorov A, Lavoie-Marchildon S, Grewal K, Bachman P, Trischler A, et al. Learning Deep Representations by Mutual Information Estimation and Maximization. *International Conference on Learning Representations (ICLR)*; 2019. https://openreview.net/forum?id=Bklr3j0cKX

54. Hassani K, Khasahmadi AH. Contrastive Multi-View Representation Learning on Graphs. *Proceedings of the 37th International Conference on Machine Learning*. 2020; https://proceedings.mlr.press/v119/hassani20a.html

55. You Y, Chen T, Sui Y, Chen T, Wang Z, Shen Y. Graph Contrastive Learning with Augmentations. *Advances in Neural Information Processing Systems*. 2020; https://proceedings.neurips.cc/paper/2020/hash/3fe230348e9a12c13120749e3f9fa4cd-Abstract.html

56. Zhang L, Dong X, Jia S, Zhang J. Node2Vec-DGI-EL: a hierarchical graph representation learning model for ingredient–disease association prediction. *Bioinforma Adv*. 2026, 6(1):vbaf216; doi: 10.1093/bioadv/vbaf216.

57. Yan R, Islam MT, Xing L. Deep representation learning of protein-protein interaction networks for enhanced pattern discovery. *Sci Adv*. 2024, 10(51):eadq4324; doi: 10.1126/sciadv.adq4324.

58. Wang P, Wen X, Li H, Lang P, Li S, Lei Y, et al. Deciphering driver regulators of cell fate decisions from single-cell transcriptomics data with CEFCON. *Nat Commun*. 2023, 14(1):8459; doi: 10.1038/s41467-023-44103-3.

59. Savage SR, Yi X, Lei JT, Wen B, Zhao H, Liao Y, et al. Pan-cancer proteogenomics expands the landscape of therapeutic targets. *Cell*. 2024, 187(16):4389–4407.e15; doi: 10.1016/j.cell.2024.05.039.

60. Feng Y, Wang J, He R, Zhou L, Li Y. A retrieval-augmented knowledge mining method with deep thinking LLMs for biomedical research and clinical support. *GigaScience*. 2025, 14:giaf109; doi: 10.1093/gigascience/giaf109.

61. Feng Y, Zhou L, Ma C, Zheng Y, He R, Li Y. Knowledge graph–based thought: a knowledge graph–enhanced LLM framework for pan-cancer question answering. *GigaScience*. 2025, 14:giae082; doi: 10.1093/gigascience/giae082.

62. Dong Y, Zhou S, Xing L, Chen Y, Ren Z, Dong Y, et al. Deep learning methods may not outperform other machine learning methods on analyzing genomic studies. *Front Genet*. 2022, 13:992070; doi: 10.3389/fgene.2022.992070.

63. Sundararajan M, Taly A, Yan Q. Axiomatic Attribution for Deep Networks. *Proceedings of the 34th International Conference on Machine Learning (PMLR)*. 2017; https://proceedings.mlr.press/v70/sundararajan17a.html.

64. Liao Y, Savage SR, Dou Y, Shi Z, Yi X, Jiang W, et al. A proteogenomics data-driven knowledge base of human cancer. *Cell Syst*. 2023, 14(9):777–787.e5; doi: 10.1016/j.cels.2023.07.007.

65. Ji Y, Dutta P, Davuluri R. Deep multi-omics integration by learning correlation-maximizing representation identifies prognostically stratified cancer subtypes. *Bioinforma Adv*. 2023, 3(1):vbad075; doi: 10.1093/bioadv/vbad075.

66. Pastrello C, Kotlyar M, Jurisica I. Informed Use of Protein–Protein Interaction Data: A Focus on the Integrated Interactions Database (IID). *Methods Mol Biol*. 2020, ; doi: 10.1007/978-1-4939-9873-9_10.

67. Gong J, Shao D, Xu K, Lu Z, Lu ZJ, Yang YT, et al. RISE: a database of RNA interactome from sequencing experiments. *Nucleic Acids Res*. 2018, 46(D1):D194-D201; doi: 10.1093/nar/gkx864.

68. Zheng Y, Luo H, Teng X, Hao X, Yan X, Tang Y, et al. NPInter v5.0: ncRNA interaction database in a new era. *Nucleic Acids Res*. 2023, 51(D1):D232-D239; doi: 10.1093/nar/gkac1002.

69. Kang J, Tang Q, He J, Li L, Yang N, Yu S, et al. RNAInter v4.0: RNA interactome repository with redefined confidence scoring system and improved accessibility. *Nucleic Acids Res*. 2022, 50(D1):D326-D332; doi: 10.1093/nar/gkab997.

70. Chang L, Zhou G, Soufan O, Xia J. miRNet 2.0: network-based visual analytics for miRNA functional analysis and systems biology. *Nucleic Acids Res*. 2020, 48(W1):W244–W251; doi: 10.1093/nar/gkaa467.

71. Lin T-Y, Goyal P, Girshick R, He K, Dollar P. Focal Loss for Dense Object Detection. *IEEE Trans Pattern Anal Mach Intell*. 2020, 42(2):318 – 327; doi: 10.1109/TPAMI.2018.2858826.

72. Akiba T, Sano S, Yanase T, Ohta T, Koyama M. Optuna: A Next-generation Hyperparameter Optimization Framework. *Proc 25th ACM SIGKDD Int Conf Knowl Discov Data Min*. 2019; doi: 10.1145/3292500.3330701.

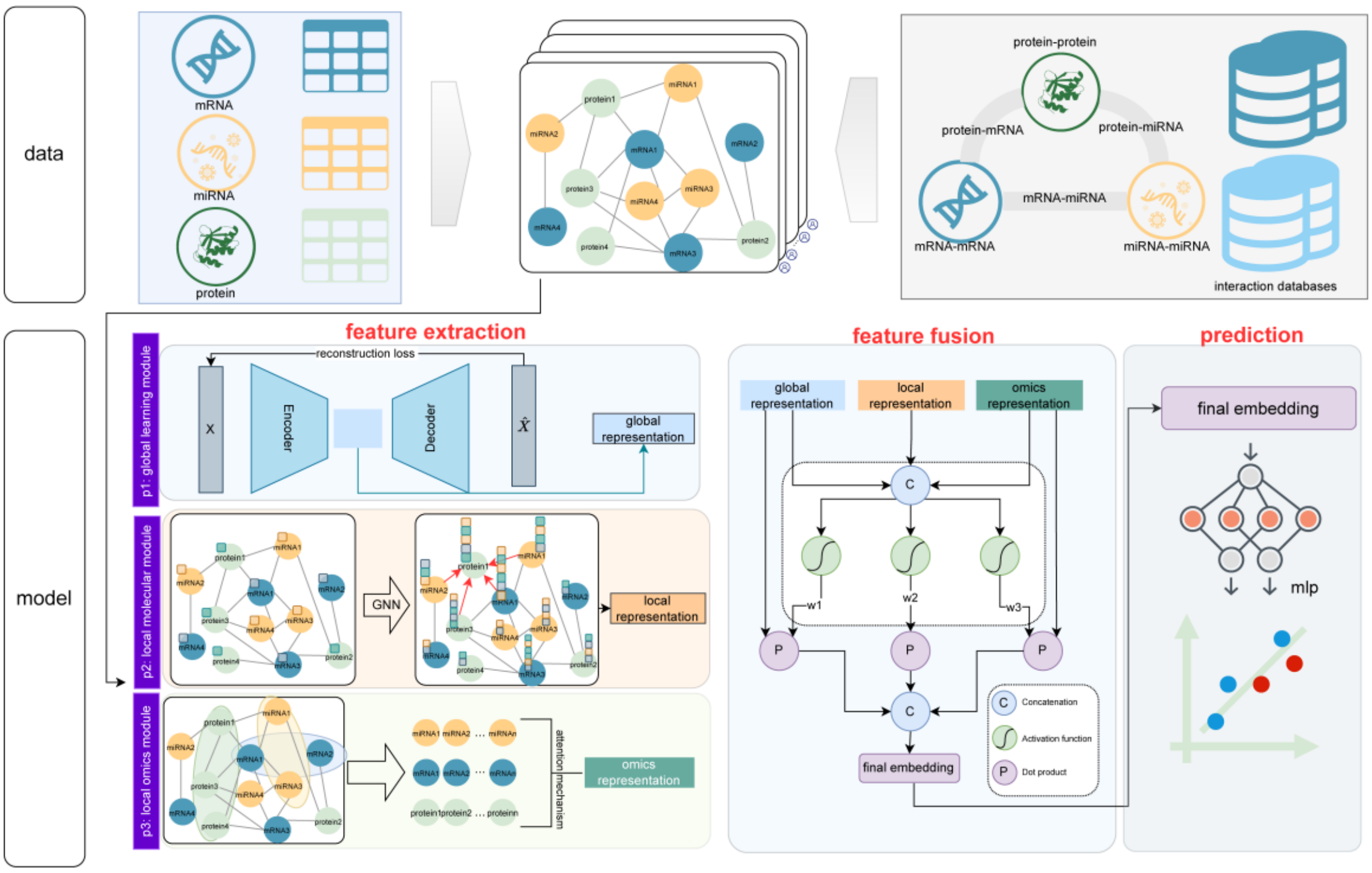

Fig 1. Overview of the GONet framework. GONet integrates multi omics profiles (mRNA, miRNA, protein) with biomolecular interaction networks for cancer classification. **Data collection and graph construction**. Multi-omics datasets and curated molecular interaction networks are used to construct sample-specific molecular interaction graphs. **Model architecture.** Multi omics matrices serve as inputs to the global learning module (AE-based), while the sample-specific molecular interaction graphs are processed by the local learning module (DGI-based). In parallel, the local omics module captures inter-omics dependencies using attention mechanism. The resulting representations are fused via feature fusion module (GFM-based) and passed to a prediction module for the downstream classification tasks.

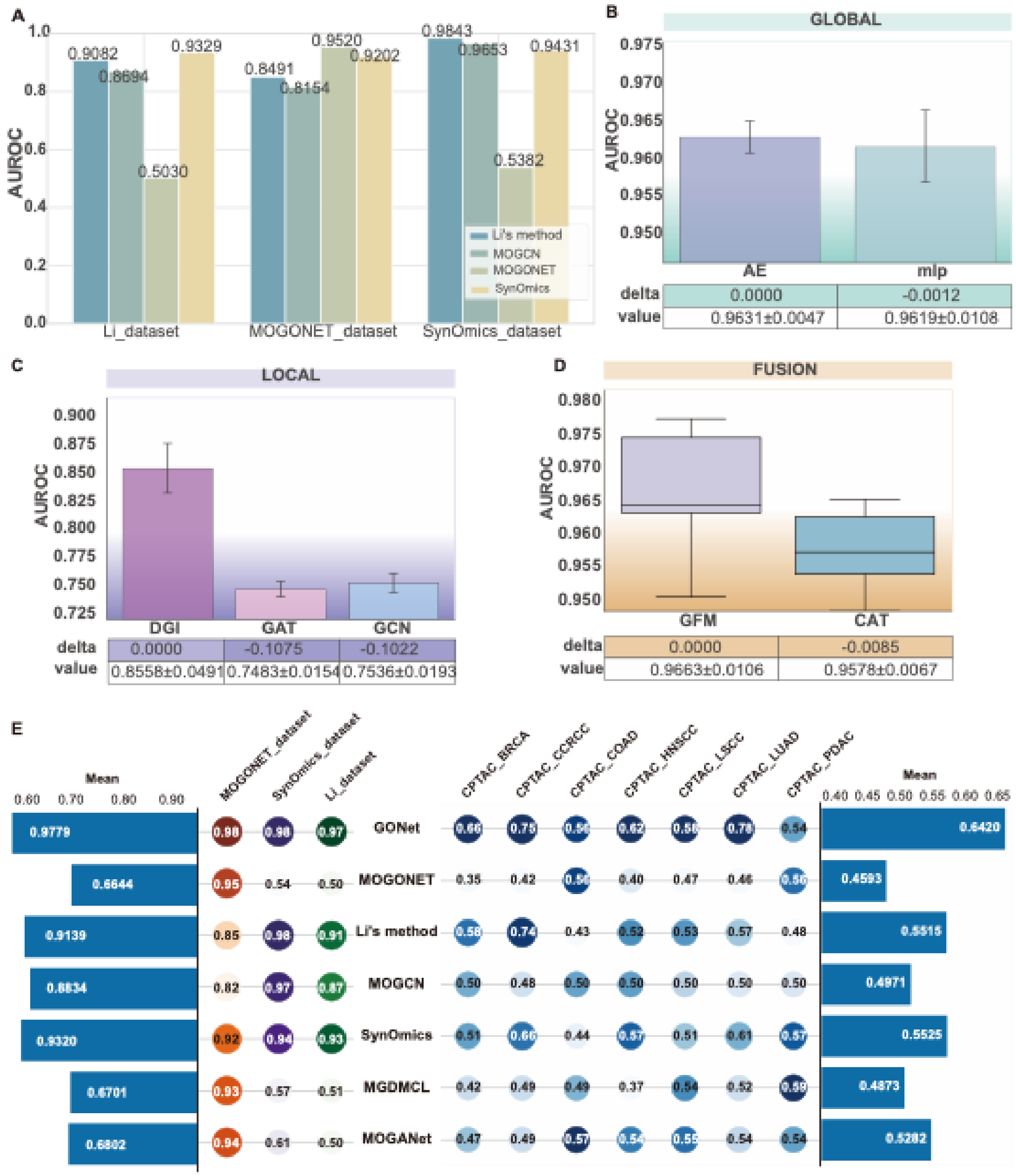

Fig 2. Module-level comparison and performance evaluation of GONet against existing GNN-based models. **A)** Comparative predictive performance of representative GNN-based methods across multiple public multi-omics cancer datasets. **B–D,** Module-specific evaluation of key architectural components in GONet on the BRCA subtype classification task using paired mRNA and miRNA data. **B)** *Global learning module.* Performance comparison of AE in GONet with a MLP baseline. **C)** *Local learning module.* Performance comparison of DGI with GCN and GAT baselines. **D)** *Feature fusion module.* Comparison between the adaptive GFM used in GONet and CAT. **E)** Generalization performance of GONet compared with representative GNN-based methods on original benchmark datasets (left) and CPTAC datasets (right).

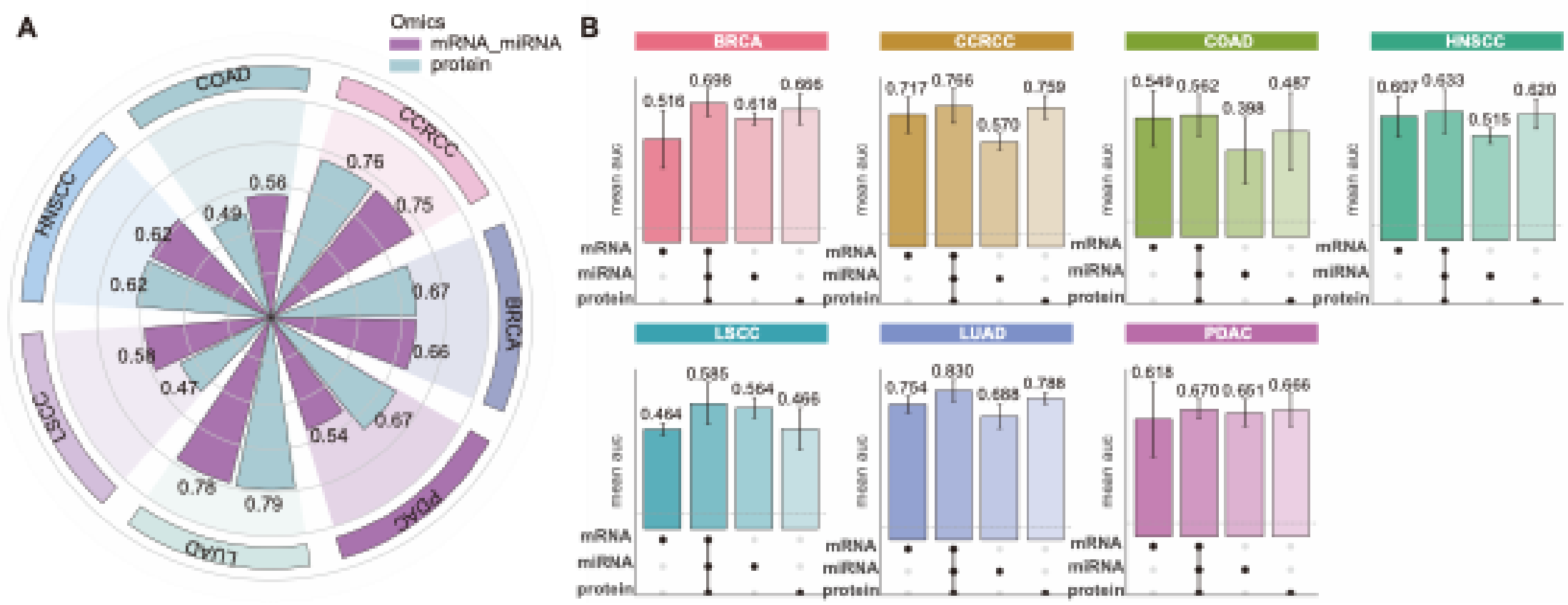

Fig 3. Evaluation of proteomics integration in GONet. A) Comparison of transcriptomics-based (mRNA-miRNA) and proteomics-based inputs for cancer stage classification across CPTAC cohort. **B)** Performance of single-, and tri-omics integration (mRNA, miRNA, protein) for cancer stage classification across CPTAC datasets.

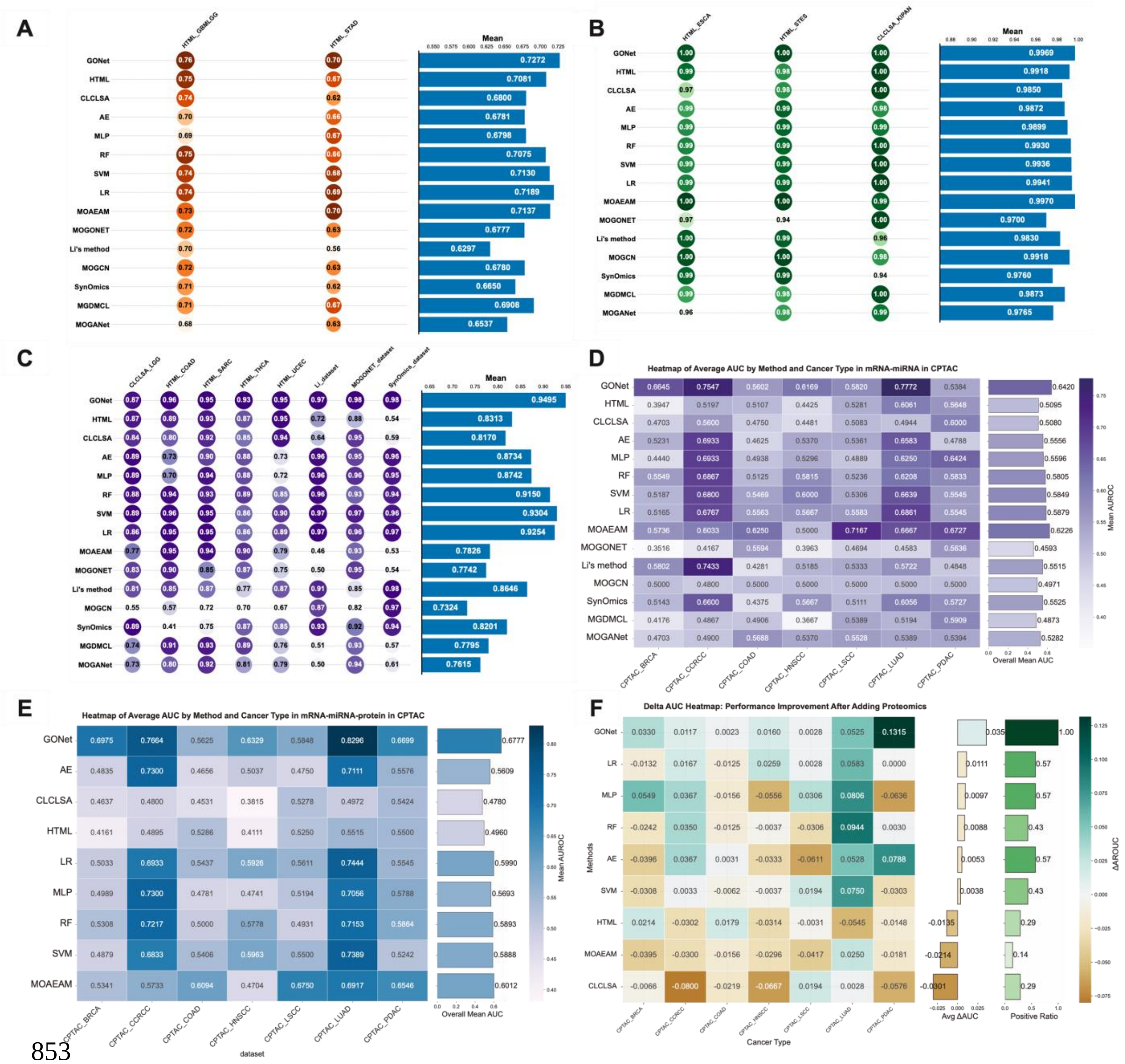

Fig 4. Systematic benchmarking of GONet performance across multi-omics cancer classification tasks. A-C) Performance comparison for bi-omics integration (mRNA-miRNA) between GONet and 14 competing methods across 13 datasets collected from previous studies grouped by task difficulty. **D)** Heatmap depicting AUROC values for bi-omics integration (mRNA-miRNA) between GONet and the 14 competing methods across seven CPTAC cancers. **E)** Heatmap showing AUROC values for tri-omics integration (mRNA, miRNA, and proteomics) between GONet and the 8 non-GNN methods across seven CPTAC cancers. **F)** Heatmap illustrating the absolute AUROC improvements (ΔAUROC) for each method after integrating proteomics data.

Description of Fig. 2: Module-specific evaluation of GONet and comparison with existing G
Table 1: Module-specific evaluation of key architectural components in GONet on the BRCA sul
Table 2: AUROC performance of GONet and representative GNN-based methods on the original

Description of Fig 3. Evaluation of proteomics integration in GONet.
Table 3: AUROC comparison of transcriptomics-based (mRNA–miRNA) and proteomics-based
Table 4: AUROC performance of single-, and tri-omics integration (mRNA, miRNA, and proteir

Description of Fig 4. Systematic benchmarking of GONet performance across multi-omics c
Table 5: AUROC performance of GONet and representative non-GNN-based methods using mR
Table 6: AUROC performance of GONet and representative non-GNN-based methods using mR
Table 7: Absolute AUROC improvements (ΔAUROC) for each method after integrating proteom

NN-based methods.

ɔtype classification task using paired mRNA and miRNA data (related to Fig. 2B–D).

datasets and CPTAC cancer cohorts for tumor subtype and stage classification (related to Fig. 2A

inputs for cancer stage classification across CPTAC cohorts (related to Fig. 3A).

ı) across CPTAC datasets (related to Fig. 3B).

ancer classification tasks

NA–miRNA data on the original datasets and CPTAC cancer cohorts for tumor subtype and stage

NA–protein–miRNA data on the original datasets and CPTAC cancer cohorts for tumor subtype a

ıics data (related to Fig. 4F).

\ and 2E).

: classification (related to Fig. 4A–D).
ınd stage classification (related to Fig. 4E).

Table 1: Module-specific evaluation of key architectural components in GONet on the BRCA su

Module	Method	AUROC
Global	AE	0.9631 ± 0.0047
	MLP	0.9619 ± 0.0108
Local	DGI	0.8558 ± 0.0491
	GCN	0.7536 ± 0.0193
	GAT	0.7483 ± 0.0154
Omics	Omics-only	0.8077 ± 0.0389
Fusion	GRU	0.9663 ± 0.0106
	CAT	0.9578 ± 0.0067

ubtype classification task using paired mRNA and miRNA data (related to Fig. 2B–D).

Table 2: AUROC performance of GONet and representative GNN-based methods on th

model	GONet	Li's method	MGDMCL	MOGANet
CPTAC_BRCA	0.6645	0.5802	0.4176	0.4703
CPTAC_CCRCC	0.7547	0.7433	0.4867	0.49
CPTAC_COAD	0.5602	0.4281	0.4906	0.5688
CPTAC_HNSCC	0.6169	0.5185	0.3667	0.537
CPTAC_LSCC	0.582	0.5333	0.5389	0.5528
CPTAC_LUAD	0.7772	0.5722	0.5194	0.5389
CPTAC_PDAC	0.5384	0.4848	0.5909	0.5394
Li_dataset	0.9663	0.9082	0.5069	0.4959
MOGONET_dataset	0.9824	0.8491	0.9329	0.9386
SynOmics_datatset	0.9849	0.9843	0.5704	0.6061

ıe original datasets and CPTAC cancer cohorts for tumor subtype and stage classification (

MOGCN	MOGONET	SynOmics
0.5	0.3516	0.5143
0.48	0.4167	0.66
0.5	0.5594	0.4375
0.5	0.3963	0.5667
0.5	0.4694	0.5111
0.5	0.4583	0.6056
0.5	0.5636	0.5727
0.8694	0.503	0.9329
0.8154	0.952	0.9202
0.9653	0.5382	0.9431

(related to Fig. 2A and 2E).

Table 3: AUROC comparison of transcriptomics-based (mRNA–miRNA) and proteomi

Cancer	mRNA_miRNA	protein	pct
BRCA	0.6645 ± 0.0432	0.6662 ± 0.0764	0.26%
CCRCC	0.7547 ± 0.067	0.7586 ± 0.0642	0.51%
COAD	0.5602 ± 0.0861	0.4875 ± 0.1801	-14.90%
HNSCC	0.6169 ± 0.1101	0.6196 ± 0.072	0.43%
LSCC	0.582 ± 0.0535	0.4657 ± 0.0963	-24.96%
LUAD	0.7772 ± 0.0464	0.7877 ± 0.03	1.33%
PDAC	0.5384 ± 0.1803	0.6664 ± 0.0847	19.21%

ics-based inputs for cancer stage classification across CPTAC cohorts (related to Fig. 3A).

Table 4: AUROC performance of single-, and tri-omics integration (mRNA, miRNA, and protein

Cancer	mRNA	mRNA_protein_miRNA	miRNA	protein
**BRCA**	0.5165 ± 0.1462	0.6975 ± 0.0683	0.6185 ± 0.0311	0.6662 ± 0.0764
**CCRCC**	0.7172 ± 0.0965	0.7664 ± 0.0947	0.5703 ± 0.0465	0.7586 ± 0.0642
**COAD**	0.5492 ± 0.1289	0.5625 ± 0.0931	0.3977 ± 0.1545	0.4875 ± 0.1801
**HNSCC**	0.6071 ± 0.1007	0.6329 ± 0.1064	0.5147 ± 0.0375	0.6196 ± 0.072
**LSCC**	0.4644 ± 0.0292	0.5848 ± 0.0981	0.564 ± 0.0484	0.4657 ± 0.0963
**LUAD**	0.7543 ± 0.0461	0.8296 ± 0.058	0.6883 ± 0.0684	0.7877 ± 0.03
**PDAC**	0.618 ± 0.201	0.6699 ± 0.0505	0.6512 ± 0.0712	0.6664 ± 0.0847

) across CPTAC datasets (related to Fig. 3B).

Table 5: AUROC performance of GONet and representative non-GNN-based methods using mR

model	GONet	AE	CLCLSA	HTML	LR	MLP
CLCLSA_KIPAN	0.9977	0.9837	0.9999	0.9996	0.9997	0.9896
CLCLSA_LGG	0.8731	0.8874	0.8406	0.8666	0.8608	0.8909
CPTAC_BRCA	0.6645	0.5231	0.4703	0.3947	0.5165	0.444
CPTAC_CCRCC	0.7547	0.6933	0.56	0.5197	0.6767	0.6933
CPTAC_COAD	0.5602	0.4625	0.475	0.5107	0.5562	0.4938
CPTAC_HNSCC	0.6169	0.537	0.4481	0.4425	0.5667	0.5296
CPTAC_LSCC	0.582	0.5361	0.5083	0.5281	0.5583	0.4889
CPTAC_LUAD	0.7772	0.6583	0.4944	0.6061	0.6861	0.625
CPTAC_PDAC	0.5384	0.4788	0.6	0.5648	0.5545	0.6424
HTML_COAD	0.9583	0.7309	0.8049	0.895	0.9529	0.7022
HTML_ESCA	0.9966	0.986	0.9731	0.9938	0.9906	0.9889
HTML_GBMLGG	0.7559	0.7001	0.7357	0.7506	0.7437	0.6874
HTML_SARC	0.9487	0.9011	0.9188	0.9306	0.9477	0.9377
HTML_STAD	0.6984	0.6561	0.6244	0.6656	0.6941	0.6723
HTML_STES	0.9965	0.9918	0.9819	0.9821	0.9918	0.9912
HTML_THCA	0.9318	0.875	0.8504	0.8715	0.8572	0.8772
HTML_UCEC	0.9505	0.7272	0.9373	0.9486	0.8883	0.7193
Li_BRCA	0.9663	0.9573	0.6384	0.7221	0.9701	0.9579
MOGONET_BRCA	0.9824	0.9472	0.9537	0.8803	0.9554	0.957
SynOmics_BRCA	0.9849	0.9611	0.5917	0.5357	0.9708	0.9514

NA–miRNA data on the original datasets and CPTAC cancer cohorts for tumor subtype and stage c

MOAEAM	RF	SVM	MGDMCL	MOGANet	Li's method	MOGCN
0.9948	0.9978	1	0.9982	0.9904	0.9607	0.9819
0.7678	0.8757	0.8922	0.7397	0.7304	0.8083	0.5493
0.5736	0.5549	0.5187	0.4176	0.4703	0.5802	0.5
0.6033	0.6867	0.68	0.4867	0.49	0.7433	0.48
0.625	0.5125	0.5469	0.4906	0.5688	0.4281	0.5
0.5	0.5815	0.6	0.3667	0.537	0.5185	0.5
0.7167	0.5236	0.5306	0.5389	0.5528	0.5333	0.5
0.6667	0.6208	0.6639	0.5194	0.5389	0.5722	0.5
0.6727	0.5833	0.5545	0.5909	0.5394	0.4848	0.5
0.9451	0.9417	0.9559	0.9059	0.804	0.8531	0.5719
0.9988	0.9901	0.9895	0.9854	0.9597	0.9959	0.9971
0.7275	0.7512	0.7431	0.7144	0.6767	0.6992	0.7217
0.9415	0.9265	0.9475	0.9319	0.9167	0.8715	0.7218
0.7	0.6638	0.6829	0.6673	0.6307	0.5601	0.6343
0.9972	0.9912	0.9912	0.9784	0.9796	0.9924	0.9965
0.8966	0.8909	0.8584	0.889	0.8085	0.7677	0.6975
0.7908	0.8528	0.901	0.759	0.792	0.8746	0.6686
0.457	0.9586	0.9659	0.5069	0.4959	0.9082	0.8694
0.9305	0.9319	0.9663	0.9329	0.9386	0.8491	0.8154
0.5319	0.9417	0.9556	0.5704	0.6061	0.9843	0.9653

classification (related to Fig. 4A–D).

MOGONET	SynOmics
0.9988	0.9385
0.825	0.8856
0.3516	0.5143
0.4167	0.66
0.5594	0.4375
0.3963	0.5667
0.4694	0.5111
0.4583	0.6056
0.5636	0.5727
0.9044	0.413
0.9696	0.9947
0.7223	0.7132
0.8515	0.7466
0.6331	0.6168
0.9415	0.9947
0.8673	0.8658
0.752	0.854
0.503	0.9329
0.952	0.9202
0.5382	0.9431

Table 6: AUROC performance of GONet and representative non-GNN-based methods using mRNA

model	GONet	AE	CLCLSA	HTML	LR	MLP
CPTAC_BRCA	0.6975	0.48351648	0.46373626	0.41611722	0.5032967	0.4989011
CPTAC_CCRCC	0.76640625	0.73	0.48	0.48948148	0.69333333	0.73
CPTAC_COAD	0.5625	0.465625	0.453125	0.52857143	0.54375	0.478125
CPTAC_HNSCC	0.63288889	0.5037037	0.38148148	0.41111111	0.59259259	0.47407407
CPTAC_LSCC	0.58477509	0.475	0.52777778	0.525	0.56111111	0.51944444
CPTAC_LUAD	0.82962963	0.71111111	0.49722222	0.55151515	0.74444444	0.70555556
CPTAC_PDAC	0.66989619	0.55757576	0.54242424	0.55	0.55454545	0.57878788

A–protein–miRNA data on the original datasets and CPTAC cancer cohorts for tumor subtyp

MOAEAM	RF	SVM
0.5341	0.53076923	0.48791209
0.5733	0.72166667	0.68333333
0.6094	0.5	0.540625
0.4704	0.57777778	0.5962963
0.675	0.49305556	0.55
0.6917	0.71527778	0.73888889
0.6546	0.58636364	0.52424242

pe and stage classification (related to Fig. 4E).

Table 7: Absolute AUROC improvements (ΔAUROC) for each method after integrating pro

model	GONet	AE	CLCLSA	HTML	LR	MLP
CPTAC_BR(	0.033	-0.0395835	-0.0065637	0.02141722	-0.0132033	0.0549011
CPTAC_CCI	0.01170625	0.0367	-0.08	-0.0302185	0.01663333	0.0367
CPTAC_CO/	0.0023	0.003125	-0.021875	0.01787143	-0.01245	-0.015675
CPTAC_HN!	0.01598889	-0.0332963	-0.0666185	-0.0313889	0.02589259	-0.0555259
CPTAC_LSC	0.00277509	-0.0611	0.01947778	-0.0031	0.00281111	0.03054444
CPTAC_LU/	0.05242963	0.05281111	0.00282222	-0.0545849	0.05834444	0.08055556
CPTAC_PD/	0.13149619	0.07877576	-0.0575758	-0.0148	4.545E-05	-0.0636121

teomics data (related to Fig. 4F).

MOAEAM	RF	SVM
-0.0395	-0.0241308	-0.0307879
-0.03	0.03496667	0.00333333
-0.0156	-0.0125	-0.006275
-0.0296	-0.0037222	-0.0037037
-0.0417	-0.0305444	0.0194
0.025	0.09447778	0.07498889
-0.0181	0.00306364	-0.0302576

Description of Supplementary Figure S1: Module ablation analyses of GONet for cancer stag
Supplementary Table S1: Ablation analysis of individual GONet modules for cancer classification
Supplementary Table S2: Quantitative summary of module-wise ablation analyses of GONet across

Description of Supplementary Figure S2: Interpretability analysis of GONet predictions usin
Supplementary Table S3: Integrated Gradients (IG) scores for the global and local modules within

Description of Supplementary Figure S6: AUROC performance gains from proteomics integr
Supplementary Table S4: AUROC performance gains from proteomics integration in GONet across
Supplementary Table S5: AUROC performance gains from proteomics integration in GONet across
Supplementary Table S6: AUROC performance gains from proteomics integration in GONet across

Description of Supplementary Figure S7: Ablation analysis of proteomics data in GONet
Supplementary Table S7: Ablation analysis of protein abundance and protein-related interaction inf

;e classification.

performance (related to Fig. S1).

; seven CPTAC cancer cohorts for cancer stage classification (related to Fig. S1).

g Integrated Gradients (IG) in an independent Fu-iCCA cohort.

GONet (related to Fig. S2).

:ation in GONet

s single-mRNA baselines (related to Fig. S6).

s single-miRNA baselines (related to Fig. S6).

s mRNA–miRNA bi-omics baselines (related to Fig. S6).

:ormation in GONet (related to Fig. S7).

Supplementary Table S1: Ablation analysis of individual GONet modules for cancer c

	noAE	GCN	GAT
**BRCA**	0.667 ± 0.0374	0.5545 ± 0.1806	0.6055 ± 0.1055
**CCRCC**	0.6656 ± 0.093	0.5484 ± 0.1287	0.675 ± 0.0477
**COAD**	0.5242±0.0446	0.4898 ± 0.0737	0.482 ± 0.0769
**HNSCC**	0.6178±0.0765	0.5902 ± 0.0967	0.5902 ± 0.1099
**LSCC**	0.5779±0.0382	0.4547 ± 0.0879	0.4893 ± 0.0753
**LUAD**	0.684 ± 0.0651	0.6204 ± 0.2266	0.7457 ± 0.0854
**PDAC**	0.5543±0.1929	0.6484 ± 0.0626	0.5419 ± 0.1764

Supplementary Table S2: Quantitative summary of module-wise ablation analyses of G

Model	Mean AUROC	Relative drop	Interpretation
Full GONet	0.6777	0	Whole GONet
noAE	0.613	10.55%	remove global representations
GCN	0.5581	21.43%	replace DGI with GCN
GAT	0.5899	14.87%	replace DGI with GAT
noOmics	0.6367	6.43%	remove omics representations
cat	0.6155	10.09%	replace gated-fusion with concatenation

lassification performance (related to Fig. S1).

no-Omics	cat	all
0.644 ± 0.0966	0.6405 ± 0.0383	**0.6975 ± 0.0683**
0.7445 ± 0.0448	0.6328 ± 0.149	**0.7664 ± 0.0947**
0.5422 ± 0.1117	0.5625 ± 0.134	**0.5625 ± 0.0931**
0.6169 ± 0.0433	0.6302 ± 0.0619	**0.6329 ± 0.1064**
0.5696 ± 0.1137	0.528 ± 0.1058	**0.5848 ± 0.0981**
0.8154 ± 0.0391	0.784 ± 0.0405	**0.8296 ± 0.058**
0.5246 ± 0.1785	0.5308 ± 0.1288	**0.6699 ± 0.0505**

GONet across seven CPTAC cancer cohorts for cancer stage classification (related to Fig. S1

l).

Supplementary Table S3: Integrated Gradients (IG) scores for the global and local modules withi

Global_Features	Type	Gene_symble	Scores	Local_Features
Q02338	prot	BDH1	0.8823	septin9
fam3c	mRNA	FAM3C	0.8708	unc5b
slc6a12	mRNA	SLC6A12	0.8519	heph
P46940	prot	IQGAP1	0.844	cnn1
kif23	mRNA	KIF23	0.8351	arrdc4
kctd11	mRNA	KCTD11	0.8325	slc25a37
cfh	mRNA	CFH	0.828	unc5c
P61158	prot	ACTR3	0.8261	hoxb4
pparg	mRNA	PPARG	0.8258	slc25a15
xpnpep2	mRNA	XPNPEP2	0.8243	mapk7
atl1	mRNA	ATL1	0.8198	eef2k
znf263	mRNA	ZNF263	0.8198	lrrfip1
ctd2207o23	mRNA	None	0.8146	tgm2
tnfsf12	mRNA	TNFSF12	0.8093	fap
P36980	prot	CFHR2	0.8066	inf2
blm	mRNA	BLM	0.8036	rap2b
c6	mRNA	C6	0.801	cfhr3
ophn1	mRNA	OPHN1	0.8002	O14925
ddx60l	mRNA	DDX60L	0.7971	Q86UX2
cdr1	mRNA	CDR1	0.7968	mgat3
hjurp	mRNA	HJURP	0.7967	samd9
epsti1	mRNA	EPSTI1	0.7948	plekhg1
casc5	mRNA	KNL1	0.7931	rasa3
ugt1a8	mRNA	UGT1A8	0.7916	tbck
P43155	prot	CRAT	0.7887	znf521
eci1	mRNA	ECI1	0.7874	amot
coq2	mRNA	COQ2	0.7864	wnt2
P07942	prot	LAMB1	0.7861	f10
med15	mRNA	MED15	0.7861	c5orf54
Q8N3C0	prot	ASCC3	0.7858	cfhr5
c12orf68	mRNA	CCDC184	0.7835	edn1
ttc38	mRNA	TTC38	0.7832	ptk7
lipg	mRNA	LIPG	0.7823	arhgef10
ccl18	mRNA	CCL18	0.7822	map6
P10109	prot	FDX1	0.7804	uroc1
Q9BZL4	prot	PPP1R12C	0.7802	ercc6l
dnm3	mRNA	DNM3	0.7799	bhlhe40
nhsl2	mRNA	NHSL2	0.7796	znf423
capg	mRNA	CAPG	0.7796	ccdc104
lzts1	mRNA	LZTS1	0.7795	dnm3

rps6ka2	mRNA	RPS6KA2	0.7767	acsm5
P09601	prot	HMOX1	0.7767	mia

ı GONet (related to Fig. S2).

Type	Gene_symble	Scores
mRNA	SEPTIN9	0.97335
mRNA	UNC5B	0.97105
mRNA	HEPH	0.96619
mRNA	CNN1	0.96572
mRNA	ARRDC4	0.96387
mRNA	SLC25A37	0.96245
mRNA	UNC5C	0.9602
mRNA	HOXB4	0.95849
mRNA	SLC25A15	0.95728
mRNA	MAPK7	0.95286
mRNA	EEF2K	0.95282
mRNA	LRRFIP1	0.95278
mRNA	TGM2	0.95158
mRNA	FAP	0.95135
mRNA	INF2	0.95087
mRNA	RAP2B	0.95046
mRNA	CFHR3	0.94947
prot	TIMM23	0.94808
prot	ITIH5	0.9469
mRNA	MGAT3	0.94684
mRNA	SAMD9	0.94587
mRNA	PLEKHG1	0.94418
mRNA	RASA3	0.9435
mRNA	TBCK	0.94214
mRNA	ZNF521	0.9419
mRNA	AMOT	0.93586
mRNA	WNT2	0.93193
mRNA	F10	0.93132
mRNA	FAM200C	0.93117
mRNA	CFHR5	0.93079
mRNA	EDN1	0.93011
mRNA	PTK7	0.92944
mRNA	ARHGEF10	0.92907
mRNA	MAP6	0.92782
mRNA	UROC1	0.92602
mRNA	ERCC6L	0.9228
mRNA	BHLHE40	0.9226
mRNA	ZNF423	0.92257
mRNA	CFAP36	0.92249
mRNA	DNM3	0.92151

mRNA	ACSM5	0.9205
mRNA	MIA	0.92042

Supplementary Table S4: AUROC performance gains from proteomics integration in GONet acro

cancer	mRNA	mRNA_protein
BRCA	0.5165 ± 0.1462	0.649 ± 0.0185
CCRCC	0.7172 ± 0.0965	0.7477 ± 0.0735
COAD	0.5492 ± 0.1289	0.5531 ± 0.0933
HNSCC	0.6071 ± 0.1007	0.6871 ± 0.0747
LSCC	0.4644 ± 0.0292	0.5031 ± 0.0588
LUAD	0.7543 ± 0.0461	0.7796 ± 0.1185
PDAC	0.618 ± 0.201	0.6284 ± 0.1265

Supplementary Table S5: AUROC performance gains from proteomics integration in GONet acro

cancer	miRNA	protein_miRNA
BRCA	0.6185 ± 0.0311	0.629 ± 0.068
CCRCC	0.5703 ± 0.0465	0.6859 ± 0.0573
COAD	0.3977 ± 0.1545	0.4969 ± 0.156
HNSCC	0.5147 ± 0.0375	0.6542 ± 0.0444
LSCC	0.564 ± 0.0484	0.5467 ± 0.09
LUAD	0.6883 ± 0.0684	0.75 ± 0.0754
PDAC	0.6512 ± 0.0712	0.6097 ± 0.0676

Supplementary Table S6: AUROC performance gains from proteomics integration in GONet acro

cancer	mRNA_miRNA	mRNA_protein_miRNA
BRCA	0.6645 ± 0.0432	0.6975 ± 0.0683
CCRCC	0.7547 ± 0.067	0.7664 ± 0.0947
COAD	0.5602 ± 0.0861	0.5625 ± 0.0931
HNSCC	0.6169 ± 0.1101	0.6329 ± 0.1064
LSCC	0.582 ± 0.0535	0.5848 ± 0.0981
LUAD	0.7772 ± 0.0464	0.8296 ± 0.058
PDAC	0.5384 ± 0.1803	0.6699 ± 0.0505

ss single-mRNA baselines (related to Fig. S6).

ss single-miRNA baselines (related to Fig. S6).

ss mRNA–miRNA bi-omics baselines (related to Fig. S6).

Supplementary Table S7: Ablation analysis of protein abundance a

cancer	mRNA_miRNA	Only abundance	Only interaction
**BRCA**	0.6645 ± 0.0432	0.6485 ± 0.0802	0.6225 ± 0.0609
**CCRCC**	0.7547 ± 0.067	0.6547 ± 0.0638	0.6625 ± 0.0473
**COAD**	0.5602 ± 0.0861	0.5391 ± 0.0456	0.4461 ± 0.1214
**HNSCC**	0.6169 ± 0.1101	0.6196 ± 0.0623	0.632 ± 0.0359
**LSCC**	0.582 ± 0.0535	0.5315 ± 0.0313	0.4865 ± 0.0592
**LUAD**	0.7772 ± 0.0464	0.7074 ± 0.0512	0.5074 ± 0.2231
**PDAC**	0.5384 ± 0.1803	0.5266 ± 0.1533	0.5536 ± 0.1354

nd protein-related interaction information in GONet (related to Fig. S7).

mRNA_protein_miRNA<br>**Both abundance and interaction**
0.6975 ± 0.0683
0.7664 ± 0.0947
0.5625 ± 0.0931
0.6329 ± 0.1064
0.5848 ± 0.0981
0.8296 ± 0.058
0.6699 ± 0.0505

Fig 1. | Overview of the GONet framework. GONet integrates multi omics profiles (mRNA, miRNA, protein) with biomolecular interaction networks for cancer

Click here to access/download;Figure;Fig1.tif

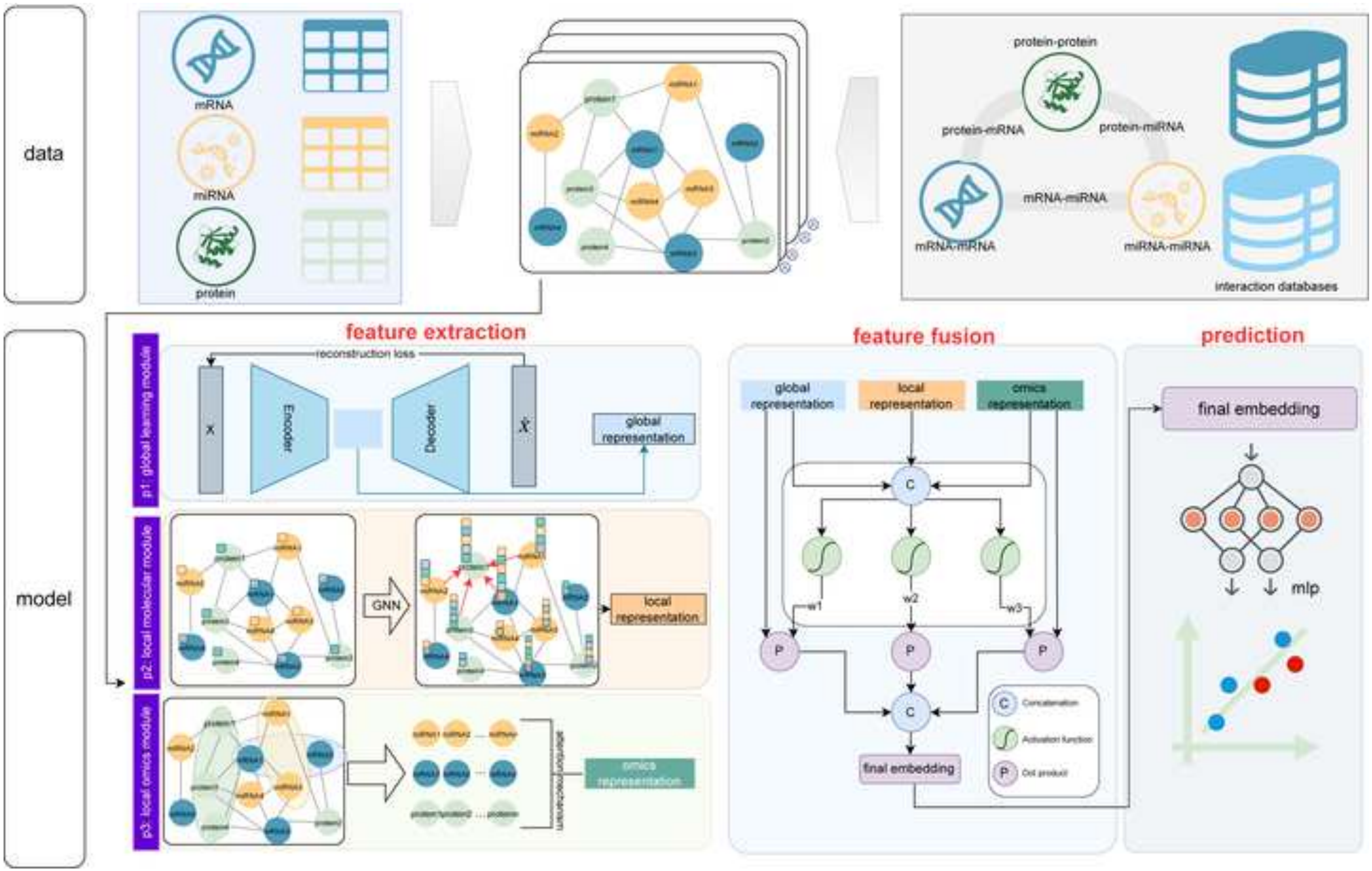

Fig 2. Module-level comparison and performance evaluation of GONet against existing GNN-based models.

Click here to access/download;Figure;R2-tiff.tif

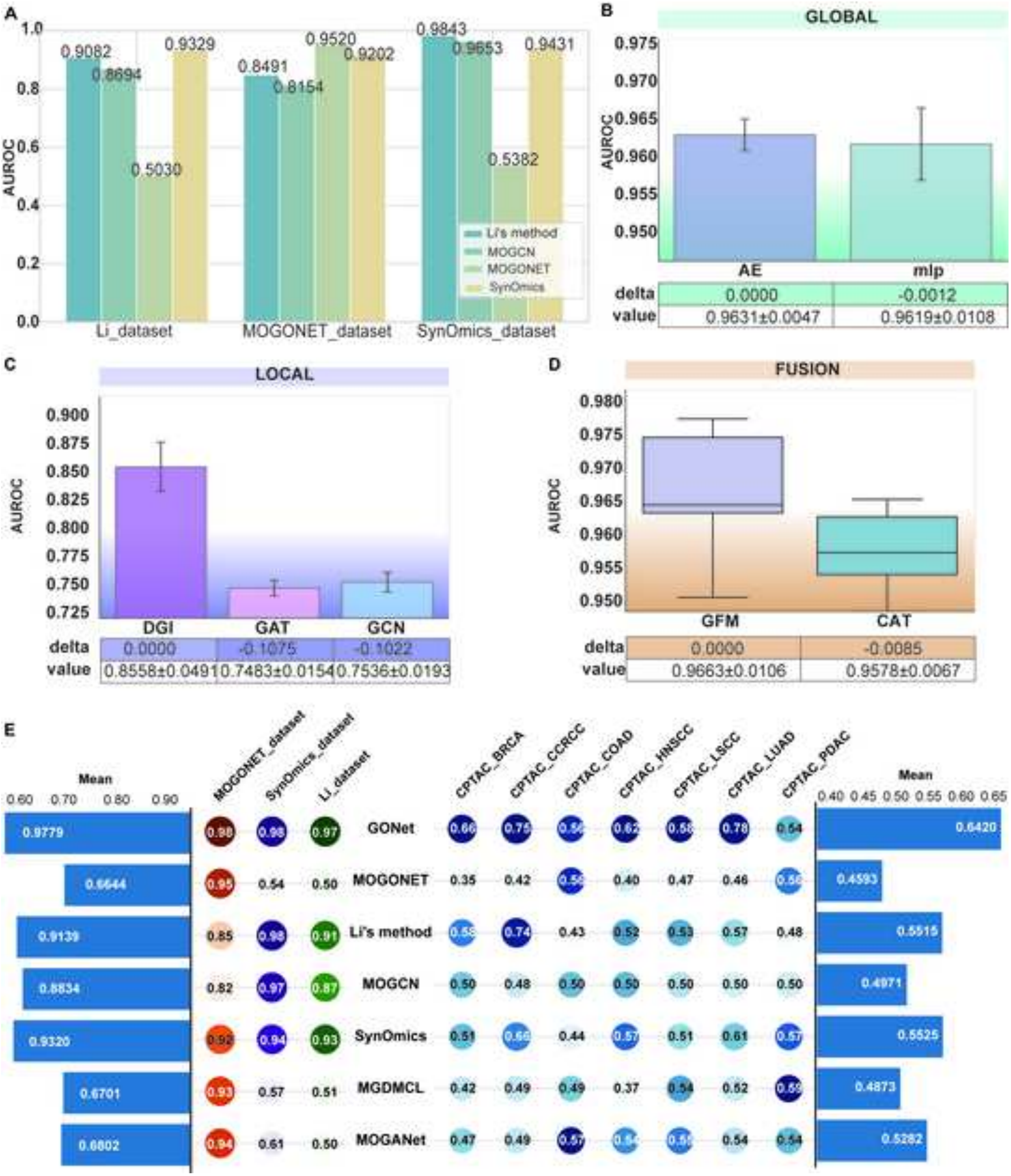

Fig 3. Evaluation of proteomics integration in GONet. Click here to access/download;Figure;R3-tiff.tif

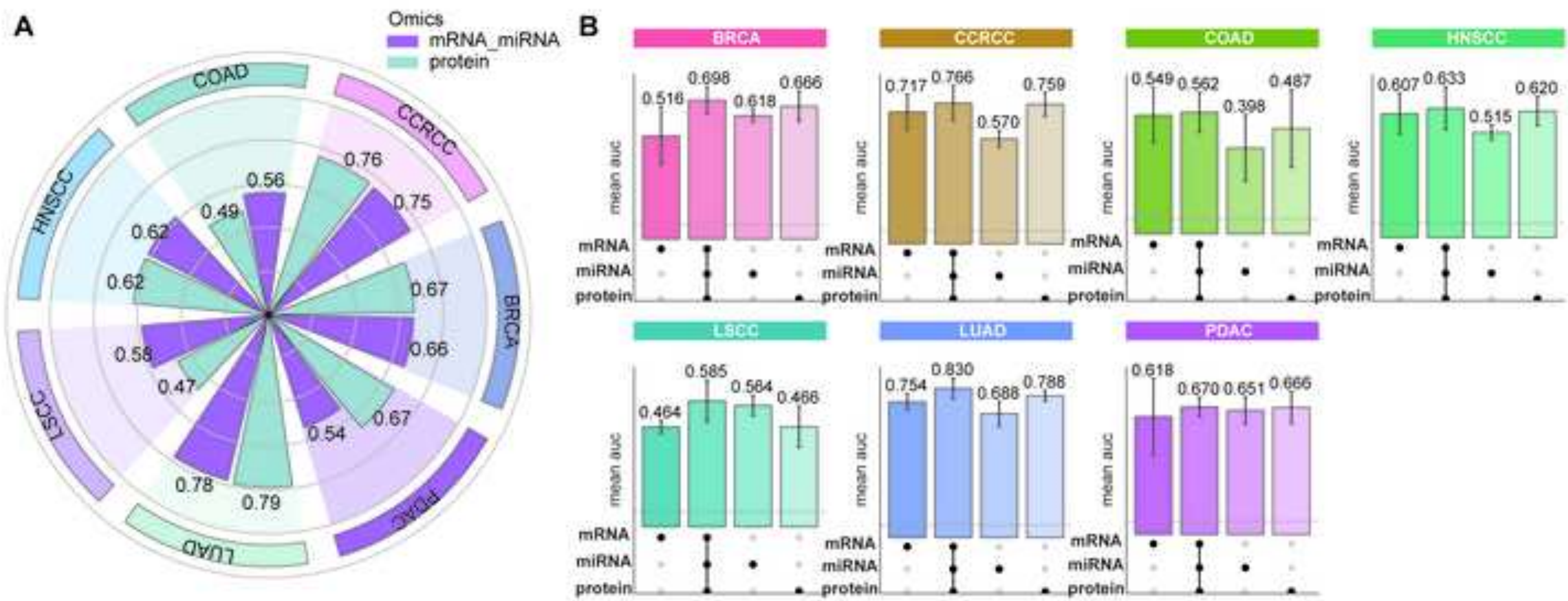

Fig 4. Systematic benchmarking of GONet performance across multi-omics cancer classification tasks.

Click here to access/download;Figure;Fig4.png

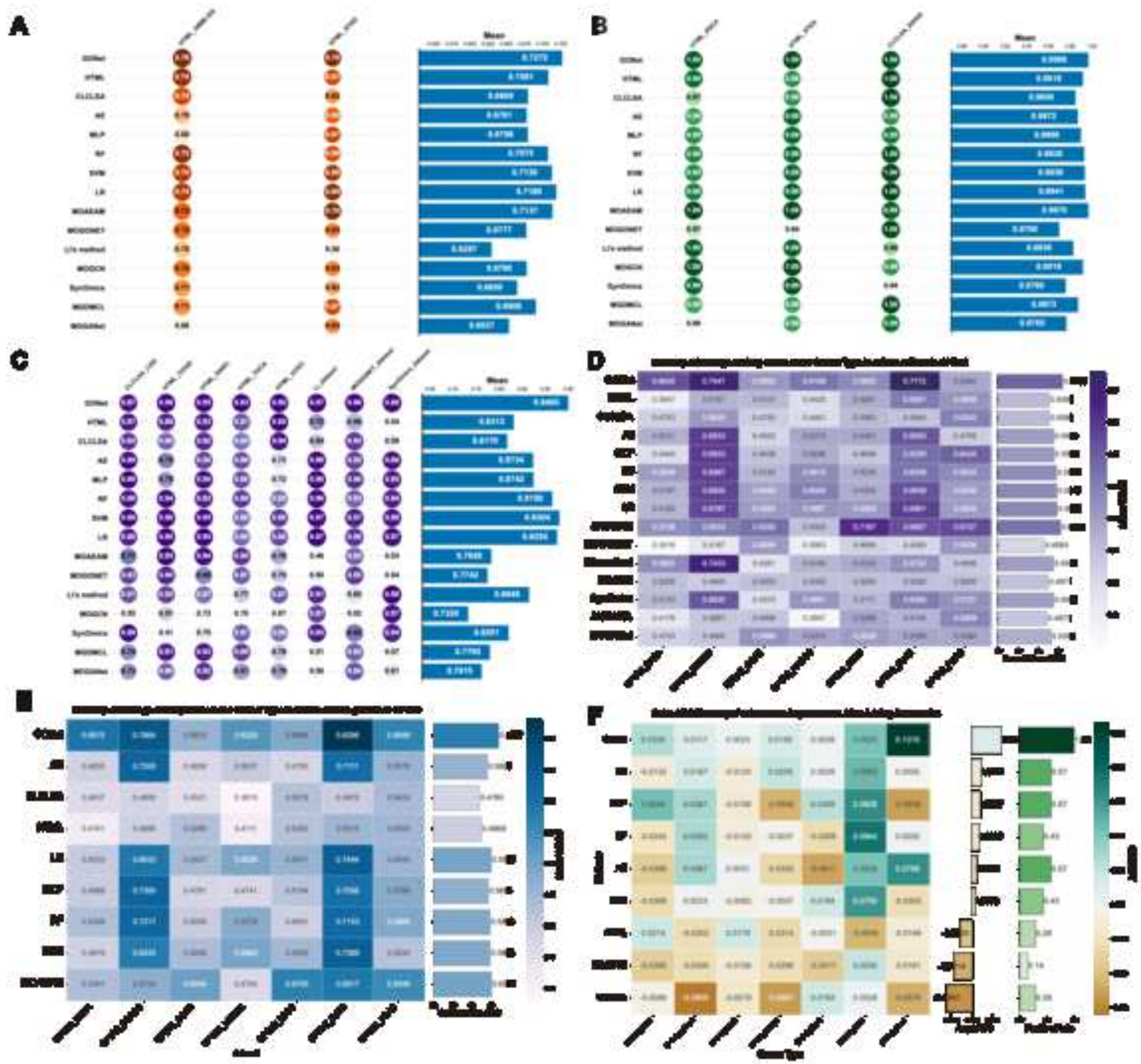

Click here to access/download
Supplementary Material
supplementary-GIGA-D-26-00172.docx

cover letter

Click here to access/download
Supplementary Material
Cover letter for Giga Science.GIGA-D-26-00172.docx

response letter

Click here to access/download
Supplementary Material
Resubmission letter.GIGA-D-26-00172.docx
