# Supplementary material for: GONet: a molecular interaction-guided graph learning framework for multi-omics cancer classification
Source: Gigascience. 2026 Oct 22;15:giag097. doi: 10.1093/gigascience/giag097 (PMC13741247; doi:10.1093/gigascience/giag097)
Supplement: Supplementary file 10 [file giag097_supplemental_file.docx]

Supplementary materials for

**GONet: A Molecular Interaction-Guided Graph Learning Framework for**

**Multi-Omics Cancer Classification**

Yiyang Chen et al.

Corresponding author: Siqi Liu, siqiliu@genomics.cn; Guixue Hou, houguixue@bgi.com; Yong Bai, Yong.bai@hotmail.cn;

**This PDF file includes:**

Figures S1 to S6

Tables S1 to S7

Supplementary Discussions: particular model interpretation

# Contents

# 1 Supplementary Figures

[Supplementary Figure S1 | Module ablation analyses of GONet for cancer stage classification.	3](#_Toc238863504)

[Supplementary Figure S2. Interpretability analysis of GONet predictions using Integrated Gradients (IG) in an independent Fu-iCCA cohort.	4](#_Toc238863505)

[Supplementary Figure S3. Illustration of GONet hyperparameters and training strategy.	5](#_Toc238863506)

[Supplementary Figure S4. Illustration of data preprocessing.	5](#_Toc238863507)

[Supplementary Figure S5. Average ranks of all methods across multiple benchmarking scenarios.	6](#_Toc238863508)

[Supplementary Figure S6| Performance gains from proteomics integration in GONet across single- and bi-omics baselines.	7](#_Toc238863509)

# 2 Supplementary Tables

[Table S1 Characteristics of public multi-omics datasets used for model training and evaluation.	7](#_Toc238862585)

[Table S2 Overview of curated molecular interaction networks used for graph construction	8](#_Toc238862586)

[Table S3 Ablation analysis of individual GONet modules for cancer classification performance.	8](#_Toc238862587)

[Table S4 Quantitative summary of module-wise ablation analyses of GONet across seven CPTAC cancer cohorts for cancer stage classification	8](#_Toc238862588)

[Table S5 Detailed hyperparameter Space for the GONet model	9](#_Toc238862589)

[Table S6 Detailed hyperparameter configurations for the GONet model	9](#_Toc238862590)

[Table S7 the IG scores of global module and local module within the GONet	10](#_Toc238862591)

# Supplementary Figures

Supplementary Figure S1 | Module ablation analyses of GONet for cancer stage classification.

To systematically quantify the contribution of each architectural component in GONet, module-wise ablation analyses were conducted on the cancer stage classification task using paired mRNA, miRNA, and proteomics data from seven cancer types in the CPTAC cohort. *A) Ablation of the global sample module:* Performance of the full GONet model was compared with a variant without the AE-based global sample learning (noAE). *B) Ablation of local molecular module:* Deep Graph Infomax (DGI) was replaced with graph convolutional network (GCN) and graph attention network (GAT), while keeping other GONet components unchanged. *C) Ablation of omics module:* The full GONet model was compared with a variant lacking the self-attention-based omics modeling (noOmics). *D) Ablation of feature fusion strategy:* The adaptive gated fusion mechanism in GONet was replaced with naive feature concatenation (cat) for integrating representations across different modules. These module-wise ablation experiments collectively quantify the individual contribution of each component and validate the overall architectural design of GONet.

Supplementary Figure S2. ****Interpretability analysis of GONet predictions using Integrated Gradients (IG) in an independent Fu-iCCA cohort.****

**(A) Receiver operating characteristic curve demonstrating the subtype classification performance of GONet in the independent Fu-iCCA cohort. GONet achieved robust classification performance with an AUROC of 0.98. (B-C) Distribution of Integrated Gradients (IG) scores for the top 100 features identified from the global and local modules, stratified by molecular modality (transcriptomic and proteomic features). The results demonstrate that highly ranked predictive features were derived from both mRNA and protein modalities. (D) Sankey diagram illustrating the functional associations of the top-ranked mRNA and protein features identified by GONet. The diagram depicts the relationships among molecular features, enriched biological functions, and corresponding Fu-iCCA molecular subtypes, highlighting the biological processes and signaling pathways associated with the identified predictive features. (E) Box plots showing the subtype-specific distribution patterns of the prioritized molecular features. The consistent differential expression patterns across Fu-iCCA subtypes further support the biological relevance of the features identified by GONet.**

Supplementary Figure S3. Illustration of GONet hyperparameters and training strategy.

Schematic diagram depicts the key hyperparameters and training procedures employed in GONet for multi-omics cancer classification tasks. (Methods for full optimization and training details).

**Supplementary Figure S4. Illustration of data preprocessing.**

**Supplementary Figure S5. Average ranks of all methods across multiple benchmarking scenarios, with lower ranks indicating superior overall performance.**

Supplementary Figure S6| Performance gains from proteomics integration in GONet across single- and bi-omics baselines.

The incremental impact of proteomics integration was assessed by measuring the change in AUROC (ΔAUROC) when proteomics data were added to baseline models. Analyses were conducted using paired data from seven cancer types in the CPTAC datasets. A) Single-omics mRNA baseline: ΔAUROC observed after adding proteomics. B) Single-omics miRNA baseline: ΔAUROC observed after introducing proteomics. C) Bi-omics (mRNA-miRNA) baseline: ΔAUROC observed after integrating proteomics. Box plots display distributions of ΔAUROC values, with mean values annotated. These analyses highlight the quantitative benefit of proteomics integration for improving multi-omics cancer classification in GONet.

# Supplementary Tables

Table S1 Characteristics of public multi-omics datasets used for model training and evaluation.

| Data Source | Classification Task | | Samples | Features before processing (mRNA, protein, miRNA) | Features after processing (mRNA, protein, miRNA) |
| --- | --- | --- | --- | --- | --- |
| **Li’s dataset** | BRCA subtype | 4 class:175, 80, 529, 197 | 981 | 17944, -, 100 | 700, -, 100 |
| **MOGONET dataset** | BRCA subtype | 5 class: 115, 131, 46, 436, 147 | 875 | 20106, -, 503 | 1000, -, 503 |
| **SynOmics dataset** | BRCA subtype | 2class:72, 240 | 312 | / | 1000, -, 200 |
| **HTML dataset** | COADREAD | 2 class: 254, 78 | 332 | 20531, -, 420 | 2000, -, 420 |
|  | ESCA subtype | 2class: 94, 89 | 183 | 20531, -, 520 | 2000, -, 520 |
|  | GBMLGG subtype | 3class: 193, 191, 127 | 511 | 20531, -, 548 | 2000, -, 548 |
|  | SARC subtype | 3class: 103, 58,96 | 257 | 20531, -, 508 | 2000, -, 508 |
|  | STAD subtype | 2class: 205, 166 | 371 | 20531, -, 507 | 2000, -, 507 |
|  | STES subtype | 2class: 94, 89 | 183 | 20531, -, 511 | 2000, -, 511 |
|  | THCA subtype | 2class: 356, 144 | 500 | 20531, -, 517 | 2000, -, 517 |
|  | UCEC subtype | 3class: 311, 98, 21 | 430 | 20531, -, 554 | 2000, - 554 |
| **CLCLSA dataset** | LGG subtype | 2 class: 246, 264 | 510 | / | 2000, -, 548 |
|  | KIPAN subtype | 3 class: 66, 318, 274 | 658 | / | 2000, -, 445 |
| **CPTAC dataset** | BRCA stage | 2 class: 72, 33 | 105 | 20374, 11977, 1747 | 1625, 918, 455 |
|  | CCRCC stage | 2 class: 48, 31 | 79 | 20374, 11260, 1747 | 1198, 1000, 484 |
|  | COAD stage | 2 class: 39, 40 | 79 | 20374, 9151, 1747 | 1359, 278, 436 |
|  | HNSCC stage | 2 class: 29, 46 | 75 | 20374, 11561, 1747 | 1150, 714, 496 |
|  | LSCC stage | 2 class: 38, 44 | 82 | 20374, 12760, 1747 | 1063, 907, 490 |
|  | LUAD stage | 2 class: 58, 30 | 88 | 20374, 12433, 1747 | 936, 475, 480 |
|  | PDAC stage | 2 class: 55, 27 | 82 | 20374, 7659, 1747 | 1300, 311, 503 |

Table S2 Overview of curated molecular interaction networks used for graph construction

| Databases | Interaction Type | Interaction Count | Total | Download |
| --- | --- | --- | --- | --- |
| BioGrid | Protein-Protein | 799,220 protein-protein | 799,220 | https://downloads.thebiogrid.org/Download/BioGRID/Release-Archive/BIOGRID-4.4.209 |
| String | Protein-Protein | 248,328 protein-protein | 248,328 | https://string-db.org/cgi/download?sessionId=bb2LTAA8FGKv/9606.protein.links.v11.5/txt.gz |
| IID | Protein-Protein | 1,209,534 protein-protein | 1,209,534 | https://iid.ophid.utoronto.ca/static/download/human.uniprot2tissues.txt.gz |
| RISE | RNA-RNA | 36248 mRNA-mRNA  33715 mRNA-miRNA  34 miRNA-miRNA | 69997 | http://rise.zhanglab.net/ |
| miRNet | miRNA-related interaction | 769799 mRNA-miRNA 1893 miRNA-protein | 771,692 | https://www.mirnet.ca/miRNet/home.xhtml |
| RNAInter | RNA-related interaction | 54689 mRNA-mRNA  2911554 mRNA-miRNA  12711409 mRNA-protein  1630 miRNA-miRNA  886248 miRNA-protein | 16,565,530 | http://www.rnainter.org/raidMedia/download/Download_data_RR.tar.gz |
| NPInter | RNA-related interaction | 502002 mRNA-miRNA  2 mRNA-protein  30 miRNA-miRNA  6817 miRNA-protein | 508,851 | http://bigdata.ibp.ac.cn/npinter5/download/file/interaction_NPInterv5.txt.gz |

Table S3 Ablation analysis of individual GONet modules for cancer classification performance.

|  | noAE | GCN | GAT | no-Omics | cat | all |
| --- | --- | --- | --- | --- | --- | --- |
| BRCA | 0.667 ± 0.0374 | 0.5545 ± 0.1806 | 0.6055 ± 0.1055 | 0.644 ± 0.0966 | 0.6405 ± 0.0383 | **0.6975 ± 0.0683** |
| CCRCC | 0.6656 ± 0.093 | 0.5484 ± 0.1287 | 0.675 ± 0.0477 | 0.7445 ± 0.0448 | 0.6328 ± 0.149 | **0.7664 ± 0.0947** |
| COAD | 0.5242±0.0446 | 0.4898 ± 0.0737 | 0.482 ± 0.0769 | 0.5422 ± 0.1117 | 0.5625 ± 0.134 | **0.5625 ± 0.0931** |
| HNSCC | 0.6178±0.0765 | 0.5902 ± 0.0967 | 0.5902 ± 0.1099 | 0.6169 ± 0.0433 | 0.6302 ± 0.0619 | **0.6329 ± 0.1064** |
| LSCC | 0.5779±0.0382 | 0.4547 ± 0.0879 | 0.4893 ± 0.0753 | 0.5696 ± 0.1137 | 0.528 ± 0.1058 | **0.5848 ± 0.0981** |
| LUAD | 0.684 ± 0.0651 | 0.6204 ± 0.2266 | 0.7457 ± 0.0854 | 0.8154 ± 0.0391 | 0.784 ± 0.0405 | **0.8296 ± 0.058** |
| PDAC | 0.5543±0.1929 | 0.6484 ± 0.0626 | 0.5419 ± 0.1764 | 0.5246 ± 0.1785 | 0.5308 ± 0.1288 | **0.6699 ± 0.0505** |

Table S4 Quantitative summary of module-wise ablation analyses of GONet across seven CPTAC cancer cohorts for cancer stage classification

| **Model** | **Mean AUROC** | **Relative drop** | **Interpretation** |
| --- | --- | --- | --- |
| Full GONet | 0.677657143 | 0 | Whole GONet |
| noAE | 0.612971429 | 10.55% | remove global representation |
| GCN | 0.558057143 | 21.43% | replace DGI with GCN |
| GAT | 0.589942857 | 14.87% | replace DGI with GAT |
| noOmics | 0.636742857 | 6.43% | remove omics representation |
| cat | 0.615542857 | 10.09% | replace gated-fusion with concatenation |

**Table S5-S6: Detailed hyperparameter configurations for the GONet model.** We employed a Bayesian optimization method Optuna to determine the optimal hyperparameters for GONet in each dataset, and subsequently report the best hyperparameters.

Table S5 Detailed hyperparameter Space for the GONet model

| hyperparameters search space | |
| --- | --- |
| AE layer numer | [2,3,4,5] |
| GNN layer number | [2,3,4,5] |
| learning rate | (1e-5,1e-3) |
| classification loss weights | (1,5) |
| reconstruction loss weights | (1,5) |
| DGI loss weights | (1,5) |
| contrast loss weights | (0.001,1) |

Table S6 Detailed hyperparameter configurations for the GONet model

|  | AE layer numer | GNN layer_numer | learning rate | cls_w | ae_w | gnn_w | cont_w |
| --- | --- | --- | --- | --- | --- | --- | --- |
| *Li_dataset* | 2 | 2 | 0.000085 | 4.9887 | 2.2869 | 1.3673 | 0.1536 |
| *BRCA* | 4 | 3 | 0.00003 | 4.1944 | 1.1645 | 2.2224 | 0.0193 |
| *COAD* | 2 | 5 | 0.000111 | 2.1581 | 2.5938 | 2.9011 | 0.4741 |
| *HNSCC* | 3 | 2 | 0.000011 | 4.1768 | 1.3136 | 2.4109 | 0.0237 |
| *CCRCC* | 4 | 3 | 0.00001 | 3.3478 | 1.5069 | 2.6903 | 0.0222 |
| *PDAC* | 2 | 2 | 0.001 | 4.0000 | 1.5000 | 2.0000 | 0.2000 |
| *LUAD* | 2 | 2 | 0.000038 | 2.3652 | 2.5623 | 2.9532 | 0.0076 |
| *LSCC* | 4 | 5 | 0.000011 | 2.7022 | 1.7538 | 1.5855 | 0.1181 |
| *iCCA_subtype* | 4 | 2 | 0.000327 | 2.3753 | 1.9516 | 1.0054 | 0.0779 |
| *MOGONET_dataset* | 2 | 2 | 0.00096 | 4.0919 | 1.0511 | 1.9888 | 0.2078 |
| *CLCLSA_KIPAN* | 3 | 2 | 0.00002 | 3.2629 | 1.7086 | 2.9400 | 0.1093 |
| *CLCLSA_LGG* | 4 | 4 | 0.000018 | 3.5508 | 1.9247 | 2.2129 | 0.0058 |
| *SynOmics_dataset* | 2 | 2 | 0.001 | 4.0000 | 1.5000 | 2.0000 | 0.2000 |
| *HTML_COAD* | 2 | 2 | 0.001 | 4.0000 | 1.5000 | 2.0000 | 0.2000 |
| *HTML_ESCA* | 4 | 2 | 0.000152 | 2.0208 | 1.3324 | 1.5353 | 0.0414 |
| *HTML_GBMLGG* | 2 | 2 | 0.001 | 4.0000 | 1.5000 | 2.0000 | 0.2000 |
| *HTML_SARC* | 2 | 2 | 0.001 | 4.0000 | 1.5000 | 2.0000 | 0.2000 |
| *HTML_STAD* | 2 | 2 | 0.001 | 4.0000 | 1.5000 | 2.0000 | 0.2000 |
| *HTML_STES* | 2 | 2 | 0.001 | 4.0000 | 1.5000 | 2.0000 | 0.2000 |
| *HTML_THCA* | 2 | 2 | 0.001 | 4.0000 | 1.5000 | 2.0000 | 0.2000 |
| *HTML_UCEC* | 2 | 2 | 0.001 | 4.0000 | 1.5000 | 2.0000 | 0.2000 |

Table S7 the IG scores of global module and local module within the GONet

| Global_Features | Type | Gene_symble | Scores |  | Local_Features | Type | Gene_symble | Scores |
| --- | --- | --- | --- | --- | --- | --- | --- | --- |
| Q02338 | prot | BDH1 | 0.8823 |  | septin9 | mRNA | SEPTIN9 | 0.97335 |
| fam3c | mRNA | FAM3C | 0.8708 |  | unc5b | mRNA | UNC5B | 0.97105 |
| slc6a12 | mRNA | SLC6A12 | 0.8519 |  | heph | mRNA | HEPH | 0.96619 |
| P46940 | prot | IQGAP1 | 0.8440 |  | cnn1 | mRNA | CNN1 | 0.96572 |
| kif23 | mRNA | KIF23 | 0.8351 |  | arrdc4 | mRNA | ARRDC4 | 0.96387 |
| kctd11 | mRNA | KCTD11 | 0.8325 |  | slc25a37 | mRNA | SLC25A37 | 0.96245 |
| cfh | mRNA | CFH | 0.8280 |  | unc5c | mRNA | UNC5C | 0.96020 |
| P61158 | prot | ACTR3 | 0.8261 |  | hoxb4 | mRNA | HOXB4 | 0.95849 |
| pparg | mRNA | PPARG | 0.8258 |  | slc25a15 | mRNA | SLC25A15 | 0.95728 |
| xpnpep2 | mRNA | XPNPEP2 | 0.8243 |  | mapk7 | mRNA | MAPK7 | 0.95286 |
| atl1 | mRNA | ATL1 | 0.8198 |  | eef2k | mRNA | EEF2K | 0.95282 |
| znf263 | mRNA | ZNF263 | 0.8198 |  | lrrfip1 | mRNA | LRRFIP1 | 0.95278 |
| ctd2207o23 | mRNA | None | 0.8146 |  | tgm2 | mRNA | TGM2 | 0.95158 |
| tnfsf12 | mRNA | TNFSF12 | 0.8093 |  | fap | mRNA | FAP | 0.95135 |
| P36980 | prot | CFHR2 | 0.8066 |  | inf2 | mRNA | INF2 | 0.95087 |
| blm | mRNA | BLM | 0.8036 |  | rap2b | mRNA | RAP2B | 0.95046 |
| c6 | mRNA | C6 | 0.8010 |  | cfhr3 | mRNA | CFHR3 | 0.94947 |
| ophn1 | mRNA | OPHN1 | 0.8002 |  | O14925 | prot | TIMM23 | 0.94808 |
| ddx60l | mRNA | DDX60L | 0.7971 |  | Q86UX2 | prot | ITIH5 | 0.94690 |
| cdr1 | mRNA | CDR1 | 0.7968 |  | mgat3 | mRNA | MGAT3 | 0.94684 |
| hjurp | mRNA | HJURP | 0.7967 |  | samd9 | mRNA | SAMD9 | 0.94587 |
| epsti1 | mRNA | EPSTI1 | 0.7948 |  | plekhg1 | mRNA | PLEKHG1 | 0.94418 |
| casc5 | mRNA | KNL1 | 0.7931 |  | rasa3 | mRNA | RASA3 | 0.94350 |
| ugt1a8 | mRNA | UGT1A8 | 0.7916 |  | tbck | mRNA | TBCK | 0.94214 |
| P43155 | prot | CRAT | 0.7887 |  | znf521 | mRNA | ZNF521 | 0.94190 |
| eci1 | mRNA | ECI1 | 0.7874 |  | amot | mRNA | AMOT | 0.93586 |
| coq2 | mRNA | COQ2 | 0.7864 |  | wnt2 | mRNA | WNT2 | 0.93193 |
| P07942 | prot | LAMB1 | 0.7861 |  | f10 | mRNA | F10 | 0.93132 |
| med15 | mRNA | MED15 | 0.7861 |  | c5orf54 | mRNA | FAM200C | 0.93117 |
| Q8N3C0 | prot | ASCC3 | 0.7858 |  | cfhr5 | mRNA | CFHR5 | 0.93079 |
| c12orf68 | mRNA | CCDC184 | 0.7835 |  | edn1 | mRNA | EDN1 | 0.93011 |
| ttc38 | mRNA | TTC38 | 0.7832 |  | ptk7 | mRNA | PTK7 | 0.92944 |
| lipg | mRNA | LIPG | 0.7823 |  | arhgef10 | mRNA | ARHGEF10 | 0.92907 |
| ccl18 | mRNA | CCL18 | 0.7822 |  | map6 | mRNA | MAP6 | 0.92782 |
| P10109 | prot | FDX1 | 0.7804 |  | uroc1 | mRNA | UROC1 | 0.92602 |
| Q9BZL4 | prot | PPP1R12C | 0.7802 |  | ercc6l | mRNA | ERCC6L | 0.92280 |
| dnm3 | mRNA | DNM3 | 0.7799 |  | bhlhe40 | mRNA | BHLHE40 | 0.92260 |
| nhsl2 | mRNA | NHSL2 | 0.7796 |  | znf423 | mRNA | ZNF423 | 0.92257 |
| capg | mRNA | CAPG | 0.7796 |  | ccdc104 | mRNA | CFAP36 | 0.92249 |
| lzts1 | mRNA | LZTS1 | 0.7795 |  | dnm3 | mRNA | DNM3 | 0.92151 |
| rps6ka2 | mRNA | RPS6KA2 | 0.7767 |  | acsm5 | mRNA | ACSM5 | 0.92050 |
| P09601 | prot | HMOX1 | 0.7767 |  | mia | mRNA | MIA | 0.92042 |
